# Synthesis and Anti-Liver Fibrosis Research of Aspartic Acid Derivatives

**DOI:** 10.3390/molecules29194774

**Published:** 2024-10-09

**Authors:** Miao Lv, Simin Guo, Hexian Yang, Yongjian Wang, Yiming Li, Yang Li, Hong Yi, Hongwei He, Zhuorong Li

**Affiliations:** Institute of Medicinal Biotechnology, Chinese Academy of Medical Sciences and Peking Union Medical College, Beijing 100050, China; miaolv0217@gmail.com (M.L.); guosimin1130@163.com (S.G.); yhx192329@163.com (H.Y.); wyjde1998@163.com (Y.W.); lym_1103@126.com (Y.L.); liy99711@163.com (Y.L.); yih0119@126.com (H.Y.)

**Keywords:** liver fibrosis, aspartic acid, IKKβ-NF-κB signaling pathway

## Abstract

Liver fibrosis plays an important role in the progression of liver disease, but there is a severe shortage of direct and efficacious pharmaceutical clinical interventions. Literature research indicates that aspartic acid exhibits hepatoprotective properties. In this paper, 32 target compounds were designed and synthesized utilizing aspartic acid as the lead compound, of which 22 were new compounds not reported in the literature. These compounds were screened for their inhibitory effects on the COL1A1 promoter to assess in vitro anti-liver fibrosis activity and summarized structure–activity relationships. Four compounds exhibited superior potency with inhibition rates ranging from 66.72% to 97.44%, substantially higher than EGCG (36.46 ± 4.64%) and L-Asp (11.33 ± 0.35%). In an LPS-induced inflammation model of LX-2 cells, both **41** and **8a** could inhibit the activation of LX-2 cells, reducing the expression of COL1A1, fibronectin, and α-SMA. Upon further investigation, **41** and **8a** ameliorated liver fibrosis by inhibiting the IKKβ-NF-κB signaling pathway to alleviate inflammatory response. Overall, the study evaluated the anti-liver fibrosis effects of aspartic acid derivatives, identified the potency of **41**, and conducted a preliminary exploration of mechanisms, laying the foundation for the discovery of novel anti-liver fibrosis agents.

## 1. Introduction

Liver diseases, including cirrhosis, viral hepatitis, and liver cancer, are responsible for over 2 million deaths annually, accounting for 4% of global mortality [1]. A key pathological response to liver injury, liver fibrosis is marked by excessive extracellular matrix (ECM) deposition, which disrupts liver parenchyma structure and can progress to cirrhosis and hepatocellular carcinoma (HCC) if untreated [2,3]. This dynamic process is potentially reversible in its early stages through the removal of causative factors; however, once fibrosis advances to cirrhosis, reversal is no longer possible, highlighting the critical need for effective management strategies [4,5,6,7]. Despite the potential for early intervention, current clinical treatments often fall short due to their slow action and limited efficacy [7].

Over 100 anti-fibrotic therapies have entered clinical trials, with some targeted drugs showing therapeutic potential, including the FXR receptor agonist obeticholic acid for primary biliary cholangitis (PBC) and the PPAR agonist Lanifibranor, both of which have shown significant efficacy in reducing fibrosis in NASH patients [8,9]. Additionally, traditional Chinese medicine (TCM) formulations and natural products, such as Fuzheng Huayu decoction and silymarin, have been used in the treatment of liver fibrosis [10,11]. However, Rezdiffra, an oral selective agonist of the thyroid hormone receptors, remains the only FDA-approved chemical drug for treating liver fibrosis in NASH, emphasizing the urgent need for novel and effective anti-fibrotic therapies [12].

Liver fibrosis primarily results from the activation of hepatic stellate cells (HSCs), extracellular matrix (ECM) deposition, and inflammation [13]. The NFκB signaling pathway is closely linked to HSC activation, driving the transcription of genes associated with inflammation and cellular stress, such as IL-6 [14]. Central to NFκB activation is the IκB kinase (IKK) complex, which consists of two serine–threonine kinases (IKKα and IKKβ) and a regulatory subunit, NEMO (also known as IKKγ). Among these, IKKβ serves as the principal catalytic subunit responsible for NFκB activation [15,16,17]. Inhibition of the IKKβ/NFκB signaling pathway has been shown to contribute to the regression of liver fibrosis and the improvement of metabolic-associated fatty liver disease (MAFLD) [18,19,20].

Aspartic acid (Asp), a non-essential amino acid with the chemical structure 2-amino-4-carboxybutyric acid, is involved in various metabolic pathways, including mitochondrial transport, the urea cycle, and nucleotide synthesis [21]. Studies have shown that L-Asp can provide hepatoprotection against acute liver injury by suppressing inflammatory responses and regulating inflammasome activity both in vitro and in vivo [22,23]. This suggests a potential role for Asp in the therapeutic management of hepatic disorders. However, its high polarity, low lipophilicity, poor cell membrane permeability, and limited hepatocyte uptake significantly restrict its therapeutic efficacy. Structural modifications of aspartic acid, such as prodrug design through medicinal chemistry, are anticipated to address these limitations.

Liver fibrosis is a common stage in the progression of all chronic liver diseases, characterized by a complex mechanism and limited treatment options. The discovery of effective drugs for liver fibrosis remains both challenging and critical. In this study, L-aspartic acid, a compound with limited anti-fibrotic activity, was used as a lead structure for further modification and optimization, resulting in derivatives with improved activity and physicochemical properties. The anti-fibrotic effects of these compounds were evaluated at both cellular and molecular levels, and their structure–activity relationships were preliminarily explored to identify the active scaffolds. Additionally, the effects of the active compounds 41 and 8a on the IKKβ-NF-κB signaling pathway were investigated, providing insights into their pharmacodynamic mechanisms. This research establishes a foundation for the development of novel anti-liver fibrosis therapies.

## 2. Results and Discussion

### 2.1. The Modification Strategies of L-Asp

**Figure 1:**
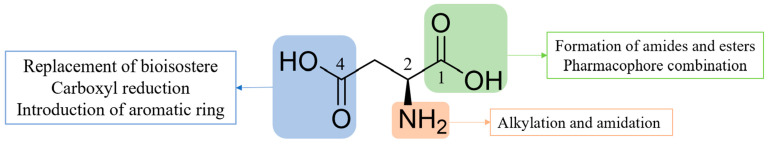
The structural modification scheme of L-Asp.

The chemical structure of L-aspartic acid is (*S*)-2-amino-4-carboxybutyric acid, which contains two carboxyl groups and one amino group. The structural modification of L-Asp targeted the three functional groups (as shown in Figure 1) to dissect the importance of each component individually. The 1-carboxyl group underwent modifications through forming amides and esters, as well as a combination of pharmacophores from biologically active compounds, to enhance activity. Alkylation and acylation were made at the 2-amino group to explore the charge and hydrogen bonding effects. The 4-carboxyl group was modified in bio-isostere substitution, carboxyl reduction, and introduction of an aromatic ring to explore the impacts of acidity, charge, and polarity.

### 2.2. The Synthesis of Target Compounds

In total, 32 derivatives were synthesized, including 22 new compounds. Based on structural disparities, four distinct synthetic routes were delineated for all compounds.

**Scheme 1:**
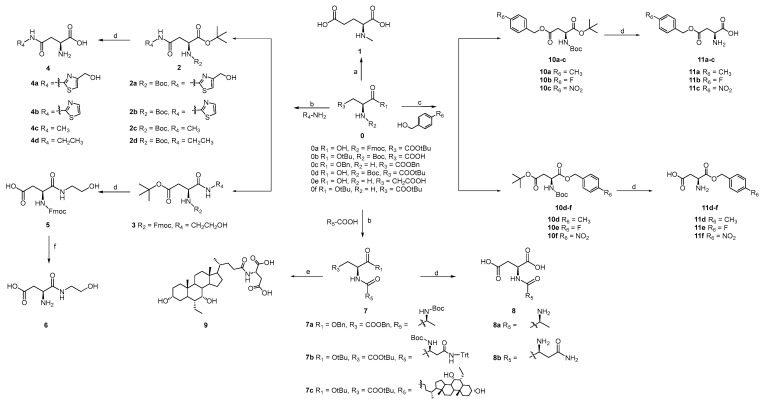
Synthetic route for compounds **1–11**. Reagents and Conditions: (a) HCHO, HOAc, NaBH_4_, MeOH, rt, 3 h; (b) HATU, DIEA, DMF, rt, 3 h; (c) DCC, DMAP, DCM, rt, 8 h; (d) 70% TFA/DCM, rt, 12 h; (e) Pd/C, NH_4_COOH, MeOH, rt, 2 h; (f) 50% Et_2_NH/DMF, rt, 2 h.

The synthetic route for the first series of derivatives is depicted in Scheme 1. The amino group of the amino acid completes the methylation of the amine with formaldehyde and sodium borohydride to obtain compound **1**. L-Asp with different protective groups led to esterification or a condensation reaction with compounds having hydroxyl, amino, and carboxyl as end groups, to afford compounds **2**, **3**, **7**, and **10**. Then the corresponding protective groups were removed by strong acid, strong base, and catalytic hydrogenation to obtain compounds **4–6**, **8–9,** and **11**, respectively.

**Scheme 2:**
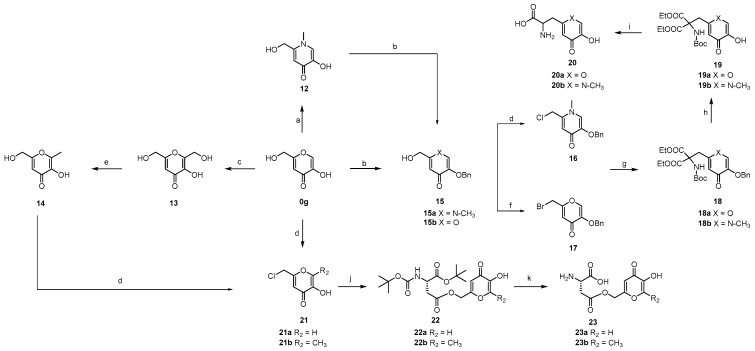
Synthetic route for compounds **12–23**. Reagents and Conditions: (a) CH_3_NH_2_, H_2_O, 90 °C, 10 h; (b) BnBr, NaOH, MeOH, 95 °C, 12 h; (c) HCHO, 1N NaOH, 12 h; (d) SOCl_2_, DCM, rt, 2 h; (e) Zn, HCl, H_2_O, 1 h; (f) PBr_3_, DCM, rt, 3 h; (g) diethyl 2-((tert-butoxy-carbonyl)amino)malonate, NaH, THF, 50 °C, 2 h; (h) Pd/C, NH_4_COOH, MeOH, rt, 2 h; (i) 6 N HCl, 90 °C, 6 h; (j) Boc-Asp-OtBu, Et_3_N, DMF, 80 °C, 12 h; (k) 70% TFA/DCM, rt, 12 h.

The second series of derivatives encompassed compounds **20** and **23**, which were obtained by replacing the 4-carboxyl group with the bio-isostere (3-hydroxypyranone). The synthetic route is shown in Scheme 2. Compounds **12**, **13**, **14**, and **21** were synthesized from Kojic acid (**0g**) as the starting material through amination, increasing carbons, and chlorination reactions. The 3-hydroxyl group was protected by a benzyl group to obtain compound **15**. The halogenation products of **15** (compounds **16,** and **17**) reacted with diethyl 2-((tert-butoxy-carbonyl)amino)malonate and NaH to produce compound **18**. Compound **19** was afforded from catalytic hydrogenation and was hydrolyzed and decarboxylated under acidic heating conditions to yield the target compound **20**. Compound **21** reacted with Boc-Asp-OtBu in a triethylamine-catalyzed substitution reaction to produce compound **22**. Finally, the Boc and tBu protecting groups were removed under strong acidic conditions, yielding the target compound **23**.

**Scheme 3:**
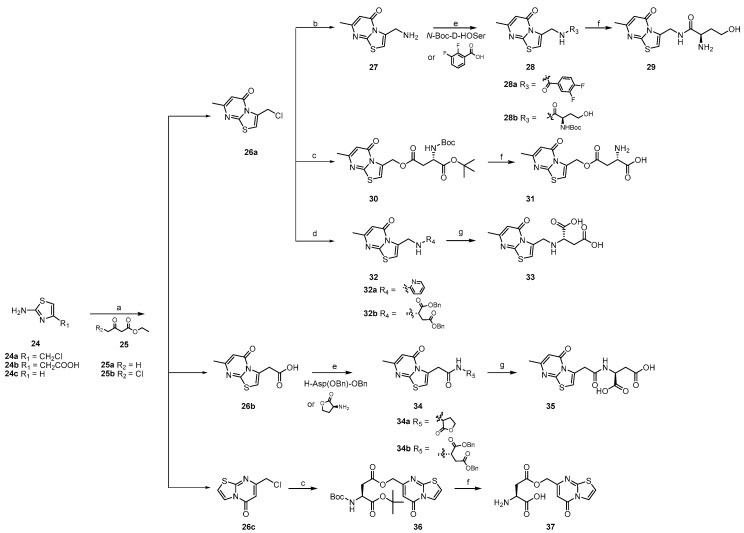
Synthetic route for compounds **24–37**. Reagents and Conditions: (a) PPA, HOAc, 110 °C, 3 h; (b) NH_3_·H_2_O, DMF, 80 °C, 12 h; (c) Boc-Asp-OtBu, Et_3_N, DMF, 80 °C, 12 h; (d) DIEA, DMF, 90 °C, 12 h; (e) HATU, DIEA, DMF, rt, 3 h; (f) 70% TFA/DCM, rt, 12 h; (g) Pd/C, NH_4_COOH, MeOH, rt, 2 h.

Considering the complex mechanism of liver fibrosis, combination therapy has more potential. According to the combination principle, L-aspartic acid was conjugated with [1,3]thiazolo[3,2-a]pyrimidin-5-one, a pivotal component of a discoidin domain receptors (DDR1/2) inhibitor with anti-liver fibrosis activity [24], to generate a third series of derivatives. The synthetic route is as shown in Scheme 3. Here, 2-Amino thiazole derivatives (**24**) were cyclized with ethyl acetoacetate (**25**) under acidic conditions to obtain compound **26** which, with its amination product **27,** led to condensation and substitution reactions with L-aspartic acid derivatives to produce **28**, **30**, **32**, **34**, and **36**. The protecting groups were removed by acidification and catalytic hydrogenation to obtain target compounds **29**, **31**, **33**, **35,** and **37**.

**Scheme 4:**
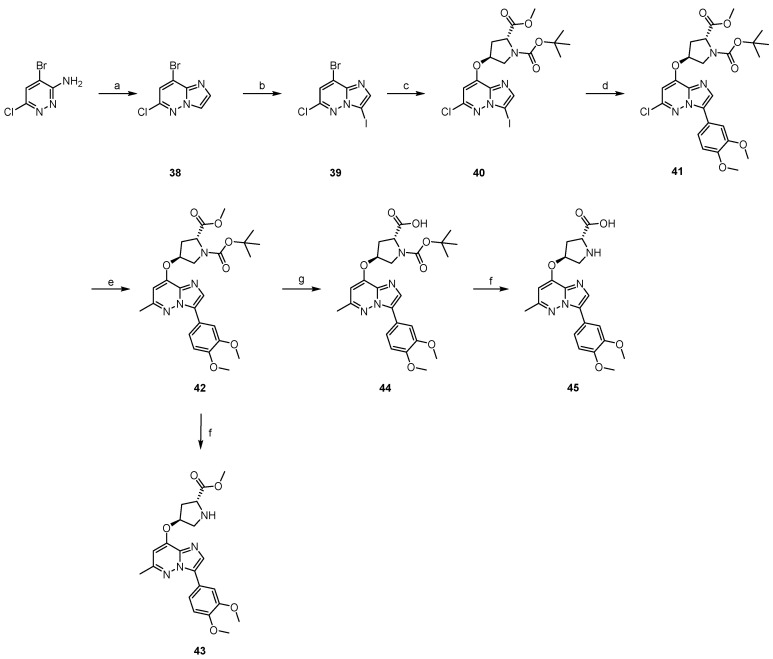
Synthetic route for compounds **38–45**. Reagents and Conditions: (a) ClCH_2_CHO, i-PrOH, 90 °C, 12 h; (b) NIS, HOAc, DMF, 65 °C, 12 h; (c) 1-(tert-butyl) 2-methyl (2*R*,4*S*)-4-hydroxypyrrolidine-1,2-dicarboxylate, K_2_CO_3_, KI, DMF, 90 °C, 36 h; (d) (3,4-dimethoxyphenyl)boronic acid, Pd(dppf)Cl_2_, Na_2_CO_3_, Dioxane, H_2_O, 95 °C, 12 h; (e) Methyl-boronic acid, K_3_PO_4_, S-Phos, Pd(OAc)_2_, HCl, Dioxane, 150 °C, 4 h; (f) 70% TFA/DCM, rt, 12 h; (g) LiOH, THF, H_2_O, rt, 12 h.

The design concept of compounds **41**, **43**, and **45** uses a rigid cyclic structure to fix relatively the stereo distance between the carboxyl, amino, and hydroxyl groups in aspartic acid. In order to facilitate ring formation, the hydroxyl and amino groups were connected by CH_2_. To utilize multiple mechanisms in combating liver fibrosis, we combined the cyclic structure with 3-phenylimidazo[1,2-b]pyridazine, a scaffold known for its efficacy against the HCV virus [25]. The synthetic route for this series of compounds is shown in Scheme 4. Compound **38** was synthesized via cyclization of 4-bromo-6-chloropyridazin-3-amine and chloro-acetaldehyde in isopropanol. Halogenation of compound **38** yielded compound **39** with iodine at the 3-position. Subsequent substitution of the bromine at the 8-position with *N*-Boc-trans-D-Hyp-OMe produced compound **40**. Suzuki coupling of iodine at the 3-position of **40** with (3,4-dimethoxyphenyl)boronic acid led to compound **41**, which was further reacted with methyl-boronic acid to yield compound **42**. The removal of the Boc group and hydrolysis of the methyl ester afforded the final compounds **43–45**.

### 2.3. In Vitro Activity Evaluation and Structure—Activity Relationships (SAR) of Target Compounds

Collagen Type I Alpha 1 (COL1A1) is a key protein upregulated during liver fibrosis at the transcriptional level. To assess preliminary anti-fibrotic effects, the impact on a COL1A1 promoter-based luciferase reporter model in human hepatic stellate LX-2 cells was evaluated [26,27,28]. A total of 32 target compounds, the lead compound L-Asp, and the positive control epigallocatechin gallate (EGCG), known for its anti-hepatic fibrotic activity [29], were tested for inhibitory effects on COL1A1 promoter activity at a concentration of 0.5 mM. As shown in Table 1, compounds **3**, **9**, **41**, and **45** demonstrated superior inhibitory rates of 66.72% to 97.44%, significantly outperforming EGCG (36.46 ± 4.64%) and L-Asp (11.33 ± 0.35%). Compounds **4b**, **4c**, **4d**, **8a**, **23b**, **28a**, **29**, **32a**, and **43** also exhibited inhibitory activity against COL1A1 with rates of 31.18% to 49.34%, comparable to EGCG and representing a 2.8- to 4.4-fold increase compared to L-Asp.

For the 1-carboxyl, the formation of a benzyl ester slightly increased activity, with the best activity when the para-position is F (**11d**, **11e**). The amide formation with ethanolamine (**6**) reduced a negative charge but retained the terminal active hydrogen, which exhibited no inhibitory effect on the COL1A1 promotor.

The 2-amino protected by Fmoc without positive charge contributes to the activity (**3** and **5**). Compound **9** (2-amino linked with bile acid) showed an 8.6-fold increase in activity, which indicated that the bile acid structure is beneficial in hepatic fibrosis activity. The enhancement of activity (2.1–4.4-fold) occurred when other amino acids formed dipeptides with L-Asp (**8a** and **8b**).

The activity of 4-carboxyl attached to aliphatic amine was higher than that attached to aromatic amine and the hydrophilic group on the aromatic ring was unfavorable for the activity (**4a**–**4d**, **37**). The inhibitory rate increased from 23.98% (**5**) to 81.54% (**3**) when 4-carboxyl was attached to tert-butyl by ester bond and remained unchanged or decreased when attached to benzyl (**11a**–**11c**). The activity was maintained when 4-carboxyl was replaced by hydroxy pyran-one or hydroxy pyridine-one via bioisosterism (**20a**, **20b**). In conclusion, the negative charge of the 4-carboxyl was not necessary for activity and this position was suitable for attachment of less polar aliphatic hydrocarbons.

For the derivatives combined by thiazolo [3,2-a]pyrimidin-5-one (an effective skeleton against hepatic fibrosis) and L-Asp, the conjugated site of the L-Asp was particularly important. The 1-carboxyl conjugated derivative was more active (**29**) than the 4-carboxyl conjugated derivative (**31**), and the 2-amino conjugated derivative lost activity (**35**).

When the rigid cyclic structure was attached to 3-phenylimidazo [1,2-b]pyridazine (an active anti-HCV skeleton), it showed a substantial increase in activity (38.23–69.64%). The carboxyl group of **45** became methyl ester, causing a 1.8-fold decrease in activity (**43**). When the amino of the tetrahydropyrrole was protected by Boc and the 6-methyl group of imidazo-pyridazine substituted by chlorine, an exponential increase in inhibitory rate occurred (**41**). Therefore, the connection of a poorly water-soluble saturated heterocycle at the 8-position of imidazo-pyridazine contributed to the activity.

In terms of SAR, the 1-carboxyl group is not essential for anti-hepatic fibrotic activity, but modifications of the carboxyl group bearing active hydrogen may not yield optimal outcomes. Modifications at the 2-amino position are crucial for anti-fibrotic activity, with both acylation and alkylation potentially enhancing inhibitory effects. The 4-carboxyl group is also non-essential for anti-liver fibrosis, and the removal of the negative charge at this position may favor increased potency.

Based on the preliminary structure–activity relationships, the dominant chemical skeleton was achieved: 1-carboxyl attached to the 4-fluorobenzyl by an ester bond, 2-amino attached to the bile acid derivatives by an amide bond, and 4-carboxyl attached to the aliphatic hydrocarbons with low polarity. In addition, a new structural skeleton was discovered: 6-chloro-3-phenylimidazo [1,2-b]pyridazine, with a poorly water-soluble saturated heterocycle linked by an ether bond. These compounds had a high inhibitory rate against the COL1A1 promotor and deserved further investigation of their structure–activity relationships.

### 2.4. Effects of Key Compounds on the Expression of Fibro-Genic Genes

**Figure 2:**
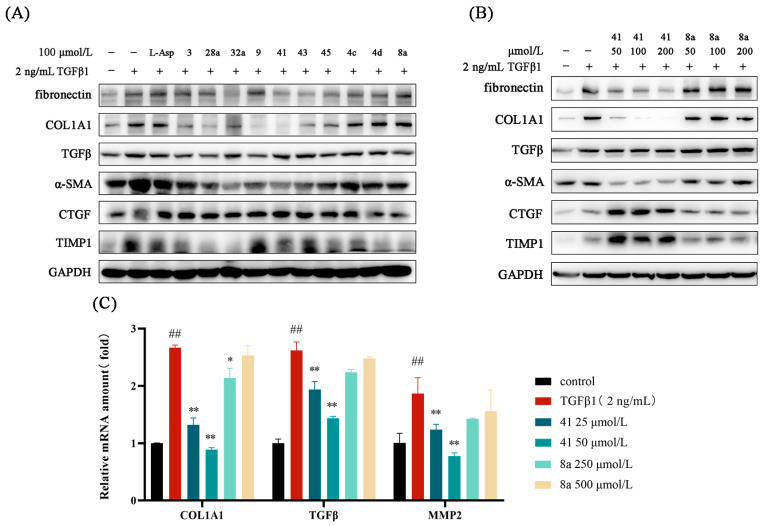
Inhibition of the target compound on fibro-genic genes in LX-2 cells 24 h after TGFβ1 (2 ng/mL) induction. (**A**) Effects of 10 target compounds and L-Asp on the expression of fibro-genic genes after 24 h induction by TGFβ1, with GAPDH as the internal reference. (**B**) Western blot analysis of the effects of different concentrations of compounds **41** and **8a** on TGFβ1-induced fibro-genic proteins. (**C**) The effects of **41** and **8a** on the mRNA levels of COL1A1, TGFβ and MMP2 were analyzed by RT–qPCR. where the values were presented as the mean ± SD and GAPDH served as the internal control, *n* = 3; ^##^ *p* < 0.01 vs. non-treated control, and * *p* < 0.05, ** *p* < 0.01 vs. TGFβ1 group.

To evaluate the anti-fibrotic efficacy in vitro, we assessed the downregulatory effects of ten active compounds (**3**, **4c**, **4d**, **8a**, **9**, **28a**, **32a**, **41**, **43** and **45**) and L-Asp on fibro-genic gene expression (fibronectin, COL1A1, TGFβ, α-SMA, CTGF, and TIMP1) using western blot analysis. LX-2 cells were activated with TGFβ1 (2 ng/mL) and concurrently treated with each compound (100 μmol/L) for 24 h. As illustrated in Figure 2, Compound **41** significantly suppressed the expression of fibro-genic genes, demonstrating a more pronounced inhibitory effect than L-Asp. Specifically, Compound **41** reduced the protein levels of fibronectin, COL1A1, and α-SMA in a dose-dependent manner following TGFβ1 stimulation (Figure 2B) and markedly decreased mRNA expression of COL1A1, TGFβ1, and MMP2 (Figure 2C). Conversely, Compound 8a exhibited no significant inhibitory effect in this cell model.

### 2.5. The Safety Profile of Representative Compounds in LX-2 Cells

The five compounds exhibiting the highest inhibition rates (**3**, **9**, **41**, **45** and **8a**) were identified as key candidates for further investigation. Their cytotoxicity in LX-2 cells was assessed across various concentrations using the SRB assay, and the cytotoxic concentrations (CC_50_) were determined. The selectivity index (SI), calculated as the ratio of CC_50_ to the semi-effective ion (IC_50_), was employed to evaluate the compounds’ effects on the COL1A1 promoter (Table 2). Results indicated that Compound **41** had a lower effective dose with an IC_50_ of 30 μmol/L, whereas Compound **8a** exhibited high in vitro safety, with a CC_50_ of 13 mmol/L. Both Compounds **41** and **8a** demonstrated high SI values and favorable safety profiles in LX-2 cells, highlighting them as the primary focus for subsequent studies.

### 2.6. The Inhibitory Effect of ***41*** and ***8a*** on LPS-Induced Inflammation in LX-2 Cells

Inflammation is crucial in the onset and progression of liver fibrosis, and fibrosis can further exacerbate tissue inflammation [30,31]. Lipopolysaccharide (LPS), a component of the cell wall of Gram-negative bacteria, has been shown to activate hepatic stellate cells (HSCs) and promote the secretion of pro-inflammatory cytokines [32,33]. L-aspartic acid (L-Asp) has been demonstrated to alleviate acute liver inflammation and downregulate inflammatory factors, contributing to hepatoprotection [22,23]. To assess the anti-inflammatory effects of Compounds **41** and **8a**, LX-2 cells were pre-incubated in serum-free medium for 24 h before LPS stimulation, followed by treatment with different concentrations of the compounds in serum-free medium containing 1 μg/mL LPS for another 24 h. Changes in inflammatory cytokine protein levels were analyzed using western blotting.

**Figure 3:**
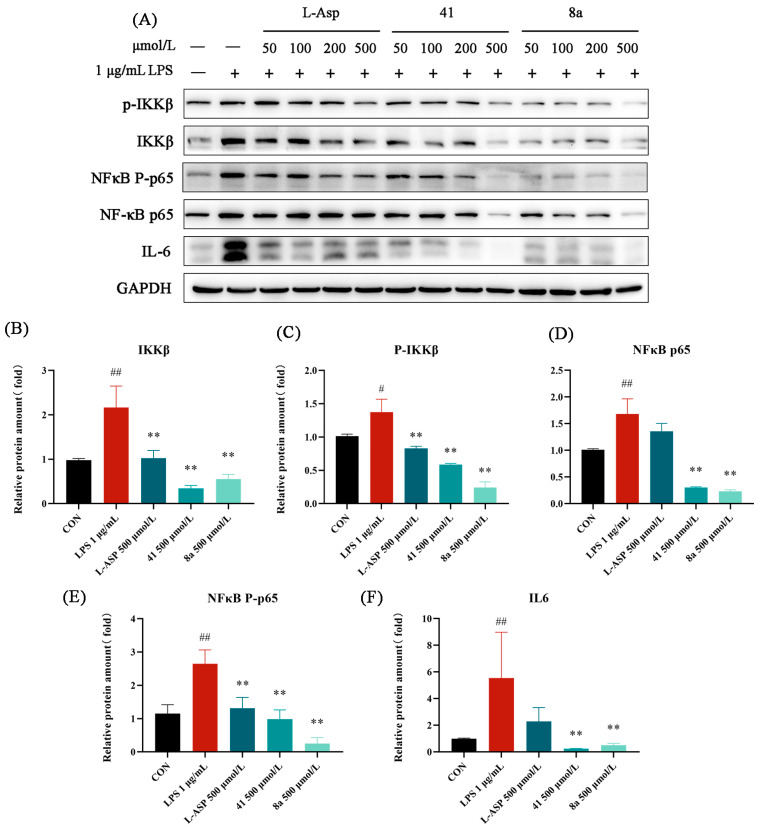
The target compound inhibits LPS-induced inflammation in LX-2 cells. (**A**) Western blot analysis showed that the target compound inhibited the protein expression of inflammatory cytokines in a dose-dependent manner at four doses of 50, 100, 200 and 500 μmol/L. (**B**–**F**) The gray values of protein bands of 500 μM concentration compounds were scanned by Image J software. Where the values were presented as the mean ± SD and GAPDH served as the internal control, *n* = 3; ^#^ *p* < 0.05, ^##^ *p* < 0.01 vs. non-treated control, and, ** *p* < 0.01 vs. LPS group.

The IKKβ-NF-κB signaling pathway has been closely linked to the severity of liver fibrosis [34,35], with IKKβ serving as the primary catalytic subunit responsible for NF-κB activation [15,16,17]. Therefore, this study investigated the NF-κB pathway by measuring IKKβ kinase activity. Results indicated that LPS significantly increased both the baseline and phosphorylation levels of IKKβ (Figure 3A). To evaluate the effects of the target compounds on LPS-induced inflammatory cytokines, four concentrations (50, 100, 200 and 500 μmol/L) were tested, and the results were analyzed by grayscale scanning of western blot bands using ImageJ software (version 1.53t), with normalization to GAPDH protein levels.

As depicted in Figure 3B, the lead compound L-Asp exhibited moderate inhibition of inflammatory factors, while Compounds **41** and **8a** demonstrated superior inhibitory effects. Treatment with Compounds **41** and **8a** led to a dose-dependent reduction in LPS-induced phosphorylation and baseline expression of IKKβ, as well as inhibition of downstream NF-κB phosphorylation and expression levels. Notably, both compounds significantly suppressed IL-6 expression. These findings suggest that Compounds **41** and **8a** effectively inhibit inflammation in LX-2 cells.

### 2.7. The Inhibitory Effect of ***41*** and ***8a*** on LPS-Induced Activation of LX-2 Cells

**Figure 4:**
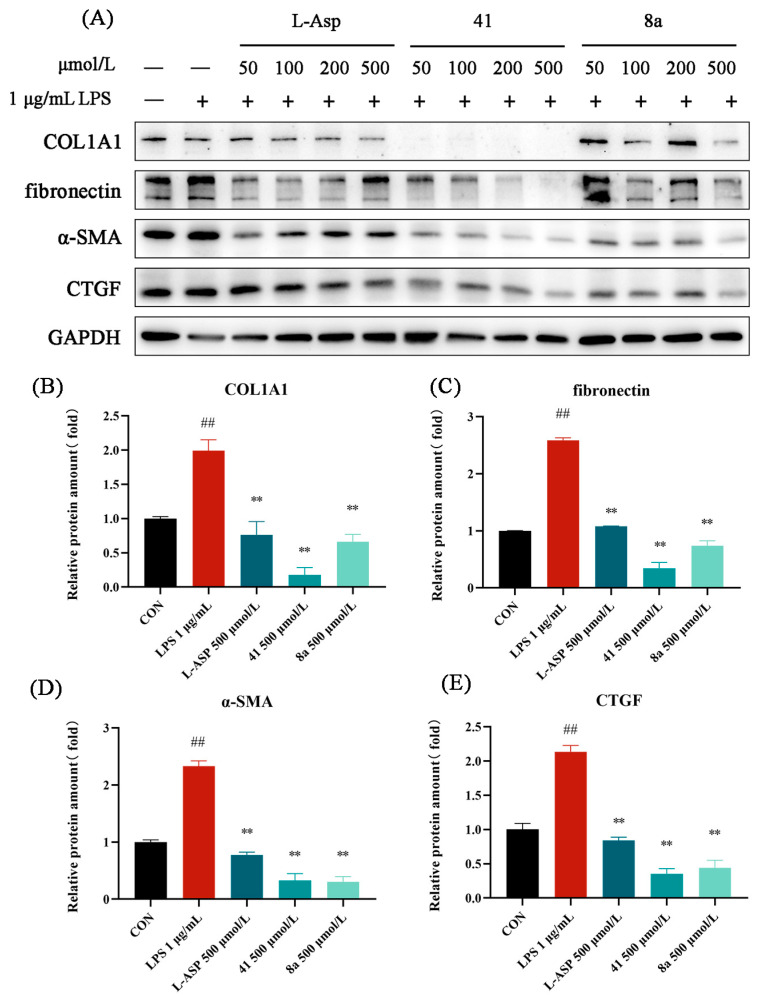
The target compound inhibits the expression of LPS-induced fibro-genic proteins in LX-2 cells. (**A**) Western blot analysis showed that the target compound inhibited inflammation-induced LX-2 cell activation and fibro-genic protein expression at four doses of 50, 100, 200, and 500 μmol/L. (**B**–**E**) The protein bands of 500 μM concentration compounds were scanned using Image J software, and the results were normalized with the protein expression of GAPDH. Where the values were presented as the mean ± SD and GAPDH served as the internal control, *n* = 3; ^##^ *p* < 0.01 vs. non-treated control, and, ** *p* < 0.01 vs. LPS group.

**Figure 5:**
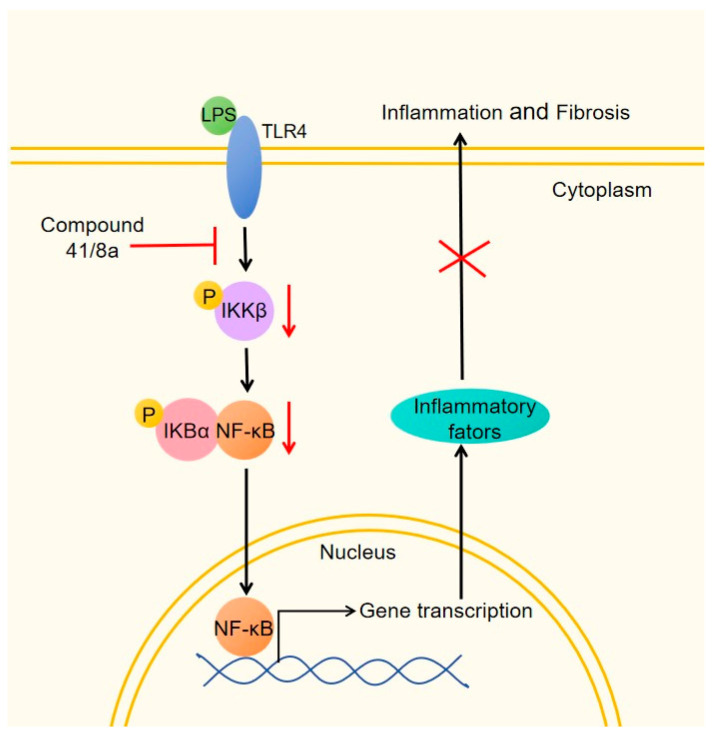
The possible anti-hepatic fibrosis mechanism of **41** and **8a**.

In a model of LPS-induced inflammation of LX-2 cells, we found that the compounds **41** and **8a** were able to inhibit the activation of LX-2 cells triggered by inflammation. Also at doses of 50, 100, 200, and 500 μmol/L, the target compounds reduced protein expression of COL1A1, fibronectin, α-SMA, and CTGF in a dose-dependent manner (Figure 4A), showing active inhibition of LX-2 cell activation and consequent decline of the fibrosis biomarker (Figure 4B–E). In particular, compound **41** exhibited potent inhibition of the fibrosis protein. Taking the results of Figure 3 and Figure 4 together, it seems that compounds **41** and **8a** alleviate inflammation and inhibit liver fibrosis through the IKKβ-NF-κB signaling pathway, as indicated in Figure 5.

## 3. Materials and Methods

### 3.1. Chemistry

All reagents and solvents were obtained commercially and used without further purification. Column chromatography was conducted using a Buchi Pure C-815 Flash system. Semi-preparative HPLC was performed on a Shimadzu Prominence LC-20AT (Kyoto, Japan) equipped with a Shim-pack PREP-ODS column (20 mm × 250 mm, 10 μm). LC−MS analysis was carried out using a Shimadzu LC-MS 2020 (Kyoto, Japan) with an electrospray ionization (ESI) source. Compound purity (>95%) was confirmed by HPLC (Agilent 1200 series, Waldbronn, Germany) using an Eclipse XDB-C18 column (4.6 mm × 150 mm, 5 μm). High-resolution mass spectra (HRMS) were recorded on an LTQ Orbitrap XL instrument (Thermo Scientific, Waltham, MA, USA). ^1^H and ^13^C NMR spectra were acquired on Bruker spectrometers operating at 400 MHz, 500 MHz, and 600 MHz (Force Biosciences). Melting points of solid samples were measured using a Mettler-Toledo MP90 apparatus (Switzerland).

#### 3.1.1. The Synthesis of **1**

Glu (0e) (59 mg, 0.4 mmol), 37% formaldehyde solution (63 μL, 0.6 mmol), and acetic acid (10 μL, 0.4 mmol) were dissolved in methanol (4 mL) and stirred at room temperature for 20 min. Sodium cyanoborohydride (106 mg, 1.6 mmol) was then added gradually until the evolution of gas ceased. The reaction mixture was heated to 40 °C and stirred for 20 h. The reaction was quenched with water (5 mL), and the solution was concentrated under reduced pressure to afford the crude product. The residue was purified by semi-preparative HPLC to yield the desired compound **1**.

*Methyl-L-glutamic acid* (**1**). White solid; yield: 45%; m.p.: 156.1–157.5 °C; ^1^H NMR (400 MHz, Deuterium Oxide) δ 3.67–3.61 (m, 1H), 2.72 (s, 3H), 2.51–2.55 (m, 2H), 2.06–2.22 (m, 2H); ^13^C NMR (101 MHz, Deuterium Oxide) δ 176.82, 172.86, 62.61, 31.79, 29.75, 24.35; HRMS: calculated for C_6_H_11_NO_4_ [M + H]^+^: 162.07608, found 162.07603.

#### 3.1.2. General Procedure for the Synthesis of **2a**, **3** and **7c**

The corresponding acids (1 mmol), amines (1.1 mmol) and HATU (1.2 mmol) were dissolved in DMF (15 mL). DIEA (3 mmol) was added and then stirred at room temperature for 3 h. The product was extracted with ethyl acetate and washed with water (10 mL × 2) and brine (10 mL × 2). The organic layer was dried over sodium sulfate and separated by silica gel column chromatography to obtain compounds **2a**, **3** and **7c**.

*Tert-butyl N^2^-(tert-butoxycarbonyl)-N^4^-(4-(hydroxymethyl)thiazol-2-yl)-L-asparaginate* (**2a**). Colorless oil; yield: 81%; ^1^H NMR (600 MHz, DMSO-*d*_6_) δ 7.21 (d, *J* = 8.4 Hz, 1H), 6.98 (s, 2H), 6.52 (s, 1H), 4.87 (s, 2H), 4.26 (q, *J* = 7.6 Hz, 1H), 2.78 (dd, *J* = 16.2, 6.2 Hz, 1H), 2.66 (dd, *J* = 16.3, 7.6 Hz, 1H), 1.40 (d, *J* = 6.0 Hz, 18H). MS (ESI) calculated for C_17_H_27_N_3_O_6_S [M + H]^+^, 402; found, 402.

*Tert-butyl (S)-3-((((9H-fluoren-9-yl)methoxy)carbonyl)amino)-4-((2-hydroxyethyl)amino)-4-oxobutanoate* (**3**). White solid; yield: 92%; m.p.: 93.2–95.6 °C; ^1^H NMR (400 MHz, Chloroform-*d*) δ 7.76 (d, *J* = 7.5 Hz, 2H), 7.57 (d, *J* = 7.4 Hz, 2H), 7.40 (t, *J* = 7.5 Hz, 2H), 7.35–7.29 (m, 2H), 6.82 (s, 1H), 5.95 (d, *J* = 7.9 Hz, 1H), 4.50 (s, 1H), 4.44 (d, *J* = 6.7 Hz, 2H), 4.21 (t, *J* = 6.7 Hz, 1H), 3.68 (t, *J* = 4.9 Hz, 2H), 3.47–3.38 (m, 2H), 2.91 (dd, *J* = 16.8, 4.2 Hz, 1H), 2.63 (dd, *J* = 16.8, 6.0 Hz, 1H), 2.34 (s, 1H), 1.44 (s, 9H); ^13^C NMR (101 MHz, Chloroform-*d*) δ 171.59, 171.27, 156.27, 143.74, 141.43, 127.91, 127.22, 125.10, 120.17, 82.20, 67.28, 61.75, 51.48, 47.24, 42.63, 37.77, 28.14; HRMS: calculated for C_25_H_30_N_2_O_6_ [M + H]^+^: 455.21766, found 455.21698.

*Dibenzyl ((4R)-4-((3R,6R,7R,10S,13R)-6-ethyl-3,7-dihydroxy-10,13-dimethylhexadecahydro-1H-cyclopenta[a]phenanthren-17-yl)pentanoyl)-L-aspartate* (**7c**). Colorless oil; yield: 67%; ^1^H NMR (500 MHz, DMSO-d_6_) δ 8.38 (d, J = 7.5 Hz, 1H), 7.36 (s, 10H), 5.10 (s, 4H), 4.73 (q, J = 6.7 Hz, 1H), 4.35–4.26 (m, 1H), 4.10–4.03 (m, 1H), 3.52 (s, 1H), 3.18 (dd, J = 18.9, 4.8 Hz, 2H), 2.94–2.76 (m, 2H), 2.15–2.01 (m, 2H), 1.93–1.67 (m, 8H), 1.46–1.18 (m, 14H), 0.90–0.84 (m, 10H), 0.61 (s, 3H). MS (ESI) calculated for C_44_H_61_NO_7_ [M + H]^+^, 716; found, 716.

#### 3.1.3. General Procedure for the Synthesis of **4a**–**4d**, **5** and **8a**–**8b**

The compounds **2a**–**2d**, **3** and **7a**, **7b** (0.4 mmol) were dissolved in the solution of 70%TFA/DCM (10 mL) and stirred at room temperature overnight. The solution was concentrated under reduced pressure to obtain the crude product. The residue was purified by semi-preparative HPLC to obtain the desired compounds **4a**–**4d**, **5** and **8a**, **8b**.

*N^4^-(4-(hydroxymethyl)thiazol-2-yl)-L-asparagine* (**4a**). Yellow solid; yield: 70%; m.p.: 257.8–259.4 °C; ^1^H NMR (600 MHz, Deuterium Oxide) δ 6.94 (s, 1H), 5.13 (s, 2H), 4.22–4.18 (m, 1H), 3.17–3.10 (m, 2H); ^13^C NMR (151 MHz, Deuterium Oxide) δ 172.16, 170.98, 170.71, 133.53, 108.18, 58.63, 50.24, 34.27; HRMS: calculated for C_8_H_11_N_3_O_4_S [M + H]^+^: 246.05430, found 246.05415.

*N^4^-(thiazol-2-yl)-L-asparagine* (**4b**). White solid; yield: 48%; m.p.: 268.9–271.2 °C; ^1^H NMR (600 MHz, Deuterium Oxide) δ 7.61 (d, *J* = 4.3 Hz, 1H), 7.42 (d, *J* = 4.3 Hz, 1H), 4.51 (t, *J* = 5.4 Hz, 1H), 3.45–3.35 (m, 2H); ^13^C NMR (151 MHz, Deuterium Oxide) δ 170.22, 169.12, 160.59, 127.50, 115.54, 48.58, 34.87; HRMS: calculated for C_7_H_9_N_3_O_3_S [M + H]^+^: 216.04374, found 216.04353.

*N^4^-methyl-L-asparagine* (**4c**). White solid; yield: 57%; m.p.: 243.2–246.7 °C; ^1^H NMR (400 MHz, Deuterium Oxide) δ 4.00 (dd, *J* = 7.6, 4.5 Hz, 1H), 2.93–2.75 (m, 2H), 2.73 (s, 3H); ^13^C NMR (101 MHz, Deuterium Oxide) δ 173.25, 172.09, 51.56, 35.10, 25.86; HRMS: calculated for C_5_H_10_N_2_O_3_ [M + H]^+^: 147.07642, found 147.07645.

*N^4^-ethyl-L-asparagine* (**4d**). White solid; yield: 61%; m.p.: 263.1–265.8 °C; ^1^H NMR (400 MHz, Deuterium Oxide) δ 4.00 (dd, *J* = 7.6, 4.5 Hz, 1H), 3.20 (q, *J* = 7.3 Hz, 2H), 2.91–2.73 (m, 2H), 1.10 (t, *J* = 7.3 Hz, 3H); ^13^C NMR (101 MHz, Deuterium Oxide) δ 173.21, 171.24, 51.58, 35.17, 34.52, 13.43; HRMS: calculated for C_6_H_12_N_2_O_3_ [M + H]^+^: 161.09207, found 161.09212.

*(S)-3-((((9H-fluoren-9-yl)methoxy)carbonyl)amino)-4-((2-hydroxyethyl)amino)-4-oxobutanoic acid* (**5**). White solid; yield: 75%; m.p.: 113.6–115.1 °C; ^1^H NMR (400 MHz, DMSO-*d*_6_) δ 12.32 (s, 1H), 7.89 (d, *J* = 7.5 Hz, 2H), 7.80 (t, *J* = 5.5 Hz, 1H), 7.72 (d, *J* = 7.4 Hz, 2H), 7.62 (d, *J* = 8.2 Hz, 1H),7.44–7.40 (m, 2H), 7.33 (t, *J* = 7.4 Hz, 2H), 4.38–4.17 (m, 5H), 3.44–3.39 (m, 4H), 3.17–3.08 (m, 2H); ^13^C NMR (151 MHz, DMSO-*d*_6_) δ 171.77, 170.74, 155.78, 143.80, 140.69, 127.64, 127.09, 125.29, 120.09, 65.75, 59.65, 51.44, 46.63, 41.60, 36.53; HRMS: calculated for C_21_H_22_N_2_O_6_ [M + H]^+^: 399.15506, found 399.15445.

*L-alanyl-L-aspartic acid* (**8a**). White solid; yield: 52%; m.p.: 182.4–184.9 °C; ^1^H NMR (400 MHz, Deuterium Oxide) δ 4.81 (d, *J* = 6.4 Hz, 1H), 4.11 (q, *J* = 7.1 Hz, 1H), 2.99 (d, *J* = 6.1 Hz, 2H), 1.55 (d, *J* = 7.1 Hz, 3H); ^13^C NMR (101 MHz, Deuterium Oxide) δ 174.33, 173.72, 170.70, 49.27, 49.00, 35.36, 16.45; HRMS: calculated for C_7_H_12_N_2_O_5_ [M + H]^+^: 205.08190, found 205.08182.

*L-asparaginyl-L-aspartic acid* (**8b**). White solid; yield: 49%; m.p.: 195.3–198.6 °C; ^1^H NMR (400 MHz, Deuterium Oxide) δ 4.81 (d, *J* = 6.0 Hz, 1H), 4.42–4.35 (m, 1H), 3.08–2.88 (m, 4H); ^13^C NMR (101 MHz, Deuterium Oxide) δ 174.33, 173.54, 172.83, 168.62, 49.75, 49.34, 35.25, 34.91; HRMS: calculated for C_8_H_13_N_3_O_6_ [M + H]^+^: 248.08771, found 248.08792.

#### 3.1.4. The Synthesis of **6**

The compound **5** (340 mg, 0.85 mmol) was dissolved in the solution of Et_2_NH: DMF = 1:1 and stirred at room temperature for 2 h. The solution was concentrated under reduced pressure to obtain the crude product. The residue was purified by semi-preparative HPLC to obtain the desired compound **6**.

*(S)-3-amino-4-((2-hydroxyethyl)amino)-4-oxobutanoic acid* (**6**). Colorless oil; yield: 66%; ^1^H NMR (400 MHz, Deuterium Oxide) δ 4.32 (dd, *J* = 7.7, 5.2 Hz, 1H), 3.69–3.66 (m, 2H), 3.42–3.38 (m, 2H), 3.07–2.91 (m, 2H); ^13^C NMR (101 MHz, Deuterium Oxide) δ 173.50, 168.93, 59.77, 49.97, 41.74, 35.41; HRMS: calculated for C_6_H_12_N_2_O_4_ [M + H]^+^: 177.08698, found 177.08693.

#### 3.1.5. The Synthesis of **9**

The compound **7c** (118 mg, 0.16 mmol) was dissolved in methanol (5 mL). The 10% Pd/C (47 mg) and ammonium formate (21 mg, 0.32 mmol) were added to the solution and stirred at room temperature for 2 h. The reaction was filtered, and the filtrate was concentrated under reduced pressure to obtain the crude product. The residue was purified by semi-preparative HPLC to obtain the desired compound **9**.

*((4R)-4-((3R,6R,7R,10S,13R)-6-ethyl-3,7-dihydroxy-10,13-dimethylhexadecahydro-1H-cyclopenta[a]phenanthren-17-yl)pentanoyl)-L-aspartic acid* (**9**). White solid; yield: 78%; m.p.: 108.2–110.6 °C; ^1^H NMR (400 MHz, DMSO-d_6_) δ 8.11 (d, J = 7.7 Hz, 1H), 4.49 (q, J = 6.5 Hz, 2H), 3.95 (s, 3H), 3.49 (s, 1H), 3.12 (s, 1H), 2.72–2.53 (m, 2H), 2.12–1.73 (m, 7H), 1.43–0.82 (m, 27H), 0.60 (s, 3H); ^13^C NMR (101 MHz, DMSO-d_6_) δ 172.59, 172.52, 171.69, 70.62, 68.41, 55.62, 50.14, 48.54, 45.35, 42.03, 41.28, 36.05, 35.55, 35.23, 34.98, 33.57, 32.67, 32.07, 31.48, 30.46, 27.87, 23.12, 22.19, 20.43, 18.37, 11.74; HRMS: calculated for C_30_H_49_NO_7_ [M + H]^+^: 536.35818, found 536.35809.

#### 3.1.6. General Procedure for the Synthesis of **11a**–**11f**

The corresponding acids (1 mmol), alcohol (1.5 mmol), DCC (1.5 mmol) and DMAP (0.2 mmol) were dissolved in DCM (15 mL) and stirred at room temperature overnight. The product was diluted with DCM and washed with water (10 mL × 2) and brine (10 mL × 2). The organic layer was dried over sodium sulfate and separated by silica gel column chromatography to obtain compounds **10a**–**10f**. Then, these compounds were dissolved in the solution of 70%TFA/DCM (10 mL) and stirred at room temperature overnight. The solution was concentrated under reduced pressure to obtain the crude product. The residue was purified by semi-preparative HPLC to obtain the desired compounds **11a**–**11f**.

*(S)-2-amino-4-((4-methylbenzyl)oxy)-4-oxobutanoic acid* (**11a**). White solid; yield: 69%; m.p.: 228.1–229.6 °C; ^1^H NMR (400 MHz, Methanol-*d*_4_) δ 7.16–7.04 (m, 4H), 5.04 (s, 2H), 4.20 (t, *J* = 5.5 Hz, 1H), 2.97–2.92 (m, 2H), 2.21 (s, 3H); ^13^C NMR (101 MHz, Methanol-*d*_4_) δ 171.14, 170.47, 139.55, 133.84, 130.20, 129.61, 68.32, 50.33, 35.09, 21.20; HRMS: calculated for C_12_H_15_NO_4_ [M + H]^+^: 238.10738, found 238.10728.

*(S)-2-amino-4-((4-fluorobenzyl)oxy)-4-oxobutanoic acid* (**11b**). White solid; yield: 73%; m.p.: 227.6–230.2 °C; ^1^H NMR (600 MHz, Methanol-*d*_4_) δ 7.41 (dd, *J* = 8.6, 5.4 Hz, 2H), 7.09 (t, *J* = 8.8 Hz, 2H), 5.19 (s, 2H), 4.32 (dd, *J* = 6.3, 4.9 Hz, 1H), 3.11–3.02 (m, 2H); ^13^C NMR (151 MHz, Methanol-*d*_4_) δ 171.04, 170.41, 165.01, 133.03, 131.74, 131.68, 116.39, 116.25, 67.57, 50.32, 35.08; HRMS: calculated for C_11_H_12_FNO_4_ [M + H]^+^: 242.08231, found 242.08250.

*(S)-2-amino-4-((4-nitrobenzyl)oxy)-4-oxobutanoic acid* (**11c**). White solid; yield: 66%; m.p.: 207.2–209.5 °C; ^1^H NMR (400 MHz, Methanol-*d*_4_) δ 8.18 (d, *J* = 8.5 Hz, 2H), 7.56 (d, *J* = 8.5 Hz, 2H), 5.28 (s, 2H), 4.29 (t, *J* = 5.6 Hz, 1H), 3.10–3.05 (m, 2H); ^13^C NMR (101 MHz, Methanol-*d*_4_) δ 170.91, 170.36, 149.18, 144.35, 129.78, 124.65, 66.81, 50.26, 35.03; HRMS: calculated for C_11_H_12_N_2_O_6_ [M + H]^+^: 269.07681, found 269.07654.

*(S)-3-amino-4-((4-methylbenzyl)oxy)-4-oxobutanoic acid* (**11d**). White solid; yield: 57%; m.p.: 205.1–207.3 °C; ^1^H NMR (400 MHz, Methanol-*d*_4_) δ 7.29–7.14 (m, 4H), 5.29–5.13 (m, 2H), 4.38 (t, *J* = 5.0 Hz, 1H), 3.14–2.96 (m, 2H), 2.29 (s, 3H); ^13^C NMR (101 MHz, Methanol-*d*_4_) δ 173.28, 169.54, 140.06, 132.59, 130.25, 129.57, 69.64, 50.27, 34.53, 21.21; HRMS: calculated for C_12_H_15_NO_4_ [M + H]^+^: 238.10738, found 238.10738.

*(S)-3-amino-4-((4-fluorobenzyl)oxy)-4-oxobutanoic acid* (**11e**). White solid; yield: 53%; m.p.: 197.6–199.5 °C; ^1^H NMR (400 MHz, Methanol-*d*_4_) δ 7.37 (dd, *J* = 8.3, 5.6 Hz, 2H), 7.04 (t, *J* = 8.7 Hz, 2H), 5.20 (q, *J* = 12.1 Hz, 2H), 4.30 (t, *J* = 5.1 Hz, 1H), 3.03–2.87 (m, 2H); ^13^C NMR (101 MHz, Methanol-*d*_4_) δ 172.50, 169.31, 165.55, 163.11, 131.98, 131.90, 116.51, 116.29, 68.61, 50.54, 34.65; HRMS: calculated for C_11_H_12_FNO_4_ [M + H]^+^: 242.08231, found 242.08250.

*(S)-3-amino-4-((4-nitrobenzyl)oxy)-4-oxobutanoic acid* (**11f**). White solid; yield: 61%; m.p.: 190.6–192.1 °C; ^1^H NMR (400 MHz, Methanol-*d*_4_) δ 8.18 (d, *J* = 8.5 Hz, 2H), 7.58 (d, *J* = 8.5 Hz, 2H), 5.35 (q, *J* = 13.2 Hz, 2H), 4.38 (t, *J* = 5.0 Hz, 1H), 3.00 (qd, *J* = 18.2, 5.1 Hz, 2H); ^13^C NMR (101 MHz, Methanol-*d*_4_) δ 172.53, 169.28, 149.31, 143.66, 130.00, 124.67, 67.79, 50.53, 34.69; HRMS: calculated for C_11_H_12_N_2_O_6_ [M + H]^+^: 269.07681, found 269.07683.

#### 3.1.7. The Synthesis of **14**

Kojic acid (0 g, 5 g, 35 mmol) and 1N NaOH (40 mL) were dissolved in water (10 mL) and stirred at 0 °C. 37% formaldehyde solution (63 μL, 0.6 mmol) was slowly added, and the reaction was stirred at room temperature overnight. The pH value of the reaction was adjusted to 4 with HCl. The solution was concentrated under reduced pressure and ice added. The white solid appeared in the bottle and was filtered to obtain compound **13**. The solid was lightly dissolved in water (8 mL) and heated to 50 °C. The zinc (4.25 g) was added to the reaction, and then concentrated hydrochloric acid was added drop by drop. The reaction was stirred at 50 °C for 1 h. The reaction was filtered and the filtrate was extracted with ethyl acetate and washed with water (100 mL × 2) and brine (100 mL × 2). The organic layer was dried over sodium sulfate and separated by silica gel column chromatography to obtain compound **14**.

*3-hydroxy-6-(hydroxymethyl)-2-methyl-4H-pyran-4-one* (**14**). White solid; yield: 70%; ^1^H NMR (500 MHz, DMSO-*d*_6_) δ 8.66 (s, 1H), 6.27 (s, 1H), 5.62 (t, *J* = 6.0 Hz, 1H), 4.27 (d, *J* = 6.1 Hz, 2H), 2.23 (s, 3H). MS (ESI) calculated for C_7_H_8_O_4_ [M + H]^+^, 157; found, 157.

#### 3.1.8. The Synthesis of **15a**

Kojic acid (0 g, 2.8 g, 20 mmol) was dissolved in hot water (10 mL) at 50 °C. A 33% methylamine solution (5 mL, 60 mmol) was added, and the reaction was stirred at 90 °C for 10 h. The solution was concentrated under reduced pressure to obtain compound **12**, which could be used for the next step without purification. Then, **12** was dissolved in methanol (10 mL). Sodium hydroxide (0.88 g, 22 mmol) aqueous solution (4 mL) was added and the reaction was stirred at 80 °C for 40 min. Benzyl bromide (2.7 mL, 22 mmol) was added drop by drop and the reaction was stirred at 95 °C for 12 h. The solution was concentrated under reduced pressure and separated by silica gel column chromatography to obtain compound **15a**.

*5-(benzyloxy)-2-(hydroxymethyl)-1-methylpyridin-4(1H)-one* (**15a**). Brown oil; yield: 24%; ^1^H NMR (600 MHz, DMSO-*d*_6_) δ 7.58 (s, 1H), 7.44–7.34 (m, 5H), 6.25 (s, 1H), 5.54 (t, *J* = 5.6 Hz, 1H), 5.01 (s, 2H), 4.39 (d, *J* = 5.5 Hz, 2H), 3.60 (s, 3H). MS (ESI) calculated for C_14_H_15_NO_3_ [M + H]^+^, 246; found, 246.

#### 3.1.9. The Synthesis of **15b**

Kojic acid (0 g, 2.84 g, 20 mmol) was dissolved in methanol (30 mL). Sodium hydroxide (0.88 g, 22 mmol) aqueous solution (4 mL) was added and the reaction was stirred at 80 °C for 40 min. Benzyl bromide (2.7 mL, 22 mmol) was added drop by drop and the reaction was stirred at 95 °C for 12 h. The reaction was concentrated under reduced pressure. The product was extracted with DCM and washed with water (100 mL × 2) and brine (100 mL × 2). The organic layer was dried over sodium sulfate and separated by silica gel column chromatography to obtain compound **15b**.

*5-(benzyloxy)-2-(hydroxymethyl)-4H-pyran-4-one* (**15b**). White solid; yield: 28%; ^1^H NMR (400 MHz, Chloroform-*d*) δ 7.52 (s, 1H), 7.41–7.30 (m, 5H), 6.51 (s, 1H), 5.08 (s, 2H), 4.46 (s, 2H). MS (ESI) calculated for C_13_H_12_O_4_ [M + H]^+^, 233; found, 233.

#### 3.1.10. The Synthesis of **17**

**15b** (464 mg, 2 mmol) was dissolved in dry DCM and the solution was stirred under nitrogen at −40 °C. Phosphorus tribromide (0.57 mL, 6 mmol) was added drop by drop, and the mixture was heated to room temperature for 3 h. The reaction was concentrated under reduced pressure. The product was extracted with DCM and washed with water (10 mL × 2) and brine (10 mL × 2). The organic layer was dried over sodium sulfate and separated by silica gel column chromatography to obtain compound **17**.

*5-(benzyloxy)-2-(bromomethyl)-4H-pyran-4-one* (**17**). White solid; yield: 45%; ^1^H NMR (500 MHz, Chloroform-*d*) δ 7.56 (s, 1H), 7.41–7.32 (m, 5H), 6.47 (s, 1H), 5.08 (s, 2H), 4.14 (s, 2H). MS (ESI) calculated for C_13_H_11_BrO_3_ [M + H]^+^, 295; found, 295.

#### 3.1.11. The Synthesis of **18a**

NaH containing 60% mineral oil (12 mg, 0.22 mmol) and dry THF (5 mL) were placed in a three-necked flask under nitrogen. The diethyl 2-((tert-butoxy-carbonyl)amino)malonate (67 mg, 0.24 mmol) in THF (2 mL) was added drop by drop and the mixture was stirred at room temperature for 30 min. **17** (59 mg, 0.2 mmol) in THF (2 mL) was added drop by drop and the mixture was heated to 50 °C for 1.5 h. The reaction was quenched by the addition of water (2 mL) and was concentrated under reduced pressure. The product was extracted with EA and washed with water (10 mL × 2) and brine (10 mL × 2). The organic layer was dried over sodium sulfate and separated by silica gel column chromatography to obtain compound **18a**.

*Diethyl 2-((5-(benzyloxy)-4-oxo-4H-pyran-2-yl)methyl)-2-((tert-butoxycarbonyl)amino)malonate* (**18a**). Colorless oil; yield: 65%; ^1^H NMR (500 MHz, Chloroform-*d*) δ 7.37 (q, *J* = 8.2, 7.8 Hz, 5H), 6.20 (s, 1H), 5.89 (s, 1H), 5.04 (s, 2H), 4.32–4.22 (m, 4H), 3.57 (s, 2H), 1.43 (s, 9H), 1.26 (d, *J* = 6.8 Hz, 6H). MS (ESI) calculated for C_25_H_31_NO_9_ [M + H]^+^, 490; found, 490.

#### 3.1.12. The Synthesis of **19b**

The compound **18b** (100 mg, 0.2 mmol) was dissolved in methanol (5 mL). The 10% Pd/C (59 mg) and ammonium formate (26 mg, 0.4 mmol) were added and stirred at room temperature for 2 h. The reaction was filtered and the filtrate was concentrated under reduced pressure to obtain the crude product. The residue was purified by semi-preparative HPLC to obtain the desired compound **19b**.

*Diethyl 2-((tert-butoxycarbonyl)amino)-2-((5-hydroxy-1-methyl-4-oxo-1,4-dihydropyridin-2-yl)methyl)malonate* (**19b**). Yellow oil; yield: 21%; ^1^H NMR (600 MHz, Methanol-*d*_4_) δ 7.49 (s, 1H), 6.29 (d, *J* = 32.4 Hz, 1H), 4.35–4.22 (m, 4H), 3.82–3.68 (m, 5H), 1.47 (s, 9H), 1.31–1.27 (m, 6H). MS (ESI) calculated for C_19_H_28_N_2_O_8_ [M + H]^+^, 413; found, 413.

#### 3.1.13. General Procedure for the Synthesis of **20a** and **20b**

**19a**, **19b** (0.06 mmol) were dissolved in 6 N HCl (5 mL) and the mixture was stirred at 100 °C for 6 h. The reaction was concentrated under reduced pressure to obtain the crude product. The residue was purified by semi-preparative HPLC to obtain the desired compounds **20a**, **20b**.

*2-amino-3-(5-hydroxy-4-oxo-4H-pyran-2-yl)propanoic acid* (**20a**). Brown solid; yield: 44%; m.p.: 217.1–219.8 °C; ^1^H NMR (600 MHz, Deuterium Oxide) δ 8.08 (s, 1H), 6.54 (s, 1H), 4.50 (t, *J* = 6.4 Hz, 1H), 3.39–3.30 (m, 2H); ^13^C NMR (151 MHz, Deuterium Oxide) δ 176.28, 170.28, 163.40, 144.81, 142.45, 114.31, 50.69, 33.52; HRMS: calculated for C_8_H_9_NO_5_ [M + H]^+^: 200.05535, found 200.05508.

*2-amino-3-(5-hydroxy-1-methyl-4-oxo-1,4-dihydropyridin-2-yl)propanoic acid* (**20b**). Yellow oil; yield: 83%; ^1^H NMR (600 MHz, Deuterium Oxide) δ 8.16 (s, 1H), 7.25 (s, 1H), 4.30–3.96 (m, 3H), 3.67–3.49 (m 2H), 3.37 (s, 1H); ^13^C NMR (151 MHz, Deuterium Oxide) δ 160.20, 145.34, 143.88, 133.44, 114.95, 52.04, 48.90, 43.81, 32.51; HRMS: calculated for C_9_H_12_N_2_O_4_ [M + H]^+^: 213.08698, found 213.08670.

#### 3.1.14. General Procedure for the Synthesis of **22a** and **22b**

Kojic acid and **14** (4.9 mmol) were dissolved in dry DCM (10 mL) and SOCl_2_ (19.5 mmol) was added drop by drop. The mixture was stirred at room temperature for 2 h. The reaction was quenched by the addition of methanol (1 mL) and was concentrated under reduced pressure to obtain compounds **21a**, **21b**, which could be used for the next step without purification. Compounds **21a**, **21b** (1 mmol) and Boc-Asp-OtBu (1.5 mmol) were dissolved in DMF (15 mL). Triethylamine (3 mmol) was added and the reaction was stirred at 80 °C for 12 h. The reaction was extracted with ethyl acetate and washed with water (30 mL × 2) and brine (30 mL × 2). The organic layer was dried over sodium sulfate and separated by silica gel column chromatography to obtain compounds **22a**, **22b**.

*1-(tert-butyl)-4-((5-hydroxy-4-oxo-4H-pyran-2-yl)methyl) (tert-butoxycarbonyl)-L-aspartate* (**22a**). Colorless oil; yield: 32%; ^1^H NMR (600 MHz, DMSO-*d*_6_) δ 9.23 (s, 1H), 8.09 (s, 1H), 7.26 (d, *J* = 8.4 Hz, 1H), 6.50 (s, 1H), 4.99 (d, *J* = 2.4 Hz, 2H), 4.25 (q, *J* = 7.6 Hz, 1H), 2.88–2.72 (m, 2H), 1.40 (d, *J* = 5.2 Hz, 18H). MS (ESI) calculated for C_19_H_27_NO_9_ [M + H]^+^, 414; found, 414.

*1-(tert-butyl)-4-((5-hydroxy-6-methyl-4-oxo-4H-pyran-2-yl)methyl) (tert-butoxycarbonyl)-L-aspartate* (**22b**). Colorless oil; yield: 72%; ^1^H NMR (600 MHz, DMSO-*d*_6_) δ 8.88 (s, 1H), 7.25 (d, *J* = 8.3 Hz, 1H), 6.43 (s, 1H), 4.97 (d, *J* = 2.7 Hz, 2H), 4.25 (q, *J* = 7.5 Hz, 1H), 2.89– 2.72 (m, 2H), 2.26 (s, 3H), 1.39 (d, *J* = 4.0 Hz, 18H). MS (ESI) calculated for C_20_H_29_NO_9_ [M + H]^+^, 428; found, 428.

#### 3.1.15. General Procedure for the Synthesis of **23a** and **23b**

The compounds **22a**, **22b** (0.4 mmol) were dissolved in the solution of 70%TFA/DCM (10 mL) and stirred at room temperature overnight. The solution was concentrated under reduced pressure to obtain the crude product. The residue was purified by the semi-preparative HPLC to obtain the desired compounds **23a**, **23b**.

*(S)-2-amino-4-((5-hydroxy-4-oxo-4H-pyran-2-yl)methoxy)-4-oxobutanoic acid* (**23a**). Brown solid; yield: 89%; m.p.: 156.3–158.7 °C; ^1^H NMR (600 MHz, Deuterium Oxide) δ 8.12 (s, 1H), 6.65 (s, 1H), 5.15 (d, *J* = 2.4 Hz, 2H), 4.45 (t, *J* = 5.5 Hz, 1H), 3.32–3.21 (m, 2H); ^13^C NMR (151 MHz, Deuterium Oxide) δ 176.38, 170.67, 170.40, 162.68, 144.99, 142.30, 112.77, 62.51, 49.17, 33.73; HRMS: calculated for C_10_H_11_NO_7_ [M + H]^+^: 258.06083, found 258.06079.

*(S)-2-amino-4-((5-hydroxy-6-methyl-4-oxo-4H-pyran-2-yl)methoxy)-4-oxobutanoic acid* (**23b**). Yellow oil; yield: 56%; ^1^H NMR (600 MHz, Deuterium Oxide) δ 6.50 (s, 1H), 5.06 (s, 2H), 4.47–4.42 (m, 1H), 3.28–3.10 (m, 2H), 2.32 (s, 3H); ^13^C NMR (151 MHz, Deuterium Oxide) δ 175.16, 170.37, 170.28, 161.34, 153.92, 141.30, 111.75, 62.52, 48.97, 33.62, 13.89; HRMS: calculated for C_11_H_13_NO_7_ [M + H]^+^: 272.07648, found 272.07639.

#### 3.1.16. General Procedure for the Synthesis of **26a**–**26c**

2-Aminothiazole with different substituents (**24a**–**24c**) (4 mmol) and ethyl acetoacetate (**25a**) or ethyl 4-chloroacetoacetate (**25b**) (20 mmol) were dissolved in acetic acid (10 mL). Polyphosphoric acid (44 mmol) was added, and the reaction was heated at 110 °C for 3 h. The reaction was extracted with ethyl acetate and washed with water (30 mL × 2) and brine (30 mL × 2). The organic layer was dried over sodium sulfate and separated by silica gel column chromatography to obtain compounds **26a**–**26c**.

*3-(chloromethyl)-7-methyl-5H-thiazolo [3,2-a]pyrimidin-5-one* (**26a**). Yellowish solid; yield: 48%; ^1^H NMR (400 MHz, DMSO-*d*_6_) δ 7.58 (s, 1H), 6.12–6.10 (m, 1H), 5.23–5.22 (m, 2H), 2.25–2.24 (m, 3H). MS (ESI) calculated for C_8_H_7_ClNO_2_S [M + H]^+^, 215; found, 215.

*2-(7-methyl-5-oxo-5H-thiazolo [3,2-a]pyrimidin-3-yl)acetic acid* (**26b**). Colorless oil; yield: 70%; ^1^H NMR (400 MHz, DMSO-*d*_6_) δ 7.20 (s, 1H), 6.02 (d, *J* = 0.7 Hz, 1H), 4.07 (s, 2H), 2.26–2.22 (m, 3H). MS (ESI) calculated for C_9_H_8_N_2_O_3_S [M + H]^+^, 225; found, 225.

*7-(chloromethyl)-5H-thiazolo [3,2-a]pyrimidin-5-one* (**26c**). White solid; yield: 63%; ^1^H NMR (400 MHz, Chloroform-*d*) δ 8.00 (d, *J* = 4.9 Hz, 1H), 7.04 (d, *J* = 4.9 Hz, 1H), 6.48 (s, 1H), 4.45 (d, *J* = 0.5 Hz, 2H). MS (ESI) calculated for C_7_H_5_ClN_2_OS [M + H]^+^, 201; found, 201.

#### 3.1.17. The Synthesis of **27**

**26a** (38 mg, 0.18 mmol) was dissolved in DMF (5 mL). Ammonium hydroxide (97 μL, 0.72 mmol) was added, and the mixture was stirred at 80 °C overnight. The pH value of the reaction was adjusted to 9 with NaHCO_3_. The product was extracted with ethyl acetate and washed with water (10 mL × 2) and brine (10 mL × 2). The organic layer was dried over sodium sulfate and separated by silica gel column chromatography to obtain compound **27**.

*3-(aminomethyl)-7-methyl-5H-thiazolo [3,2-a]pyrimidin-5-one* (**27**). Colorless oil; yield: 80%; ^1^H NMR (400 MHz, DMSO-*d*_6_) δ 7.20 (s, 1H), 6.11–6.09 (m, 1H), 4.07 (s, 2H), 2.27–2.25 (m, 3H). MS (ESI) calculated for C_8_H_9_N_3_OS [M + H]^+^, 196; found, 196.

#### 3.1.18. General Procedure for the Synthesis of **28a**, **28b** and **34a**, **34b**

The corresponding acids (1 mmol), amines (1.1 mmol) and HATU (1.2 mmol) were dissolved in DMF (15 mL). DIEA (3 mmol) was added and then stirred at room temperature for 3 h. The product was extracted with ethyl acetate and washed with water (10 mL × 2) and brine (10 mL × 2). The organic layer was dried over sodium sulfate and separated by silica gel column chromatography to obtain compounds **28a**, **28b** and **34a**, **34b**.

*2,3-difluoro-N-((7-methyl-5-oxo-5H-thiazolo [3,2-a]pyrimidin-3-yl)methyl)benzamide* (**28a**). White solid; yield: 44%; m.p.: 218.0–220.2 °C; ^1^H NMR (400 MHz, DMSO-*d*_6_) δ 8.98 (t, *J* = 5.5 Hz, 1H), 7.98–7.90 (m, 1H), 7.80–7.74 (m, 1H), 7.57 (dt, *J* = 10.5, 8.4 Hz, 1H), 7.15 (s, 1H), 6.09 (s, 1H), 4.97–4.91 (m, 2H), 2.24 (s, 3H); ^13^C NMR (101 MHz, DMSO-*d*_6_) δ 164.14, 163.03, 160.33, 150.32, 135.77, 131.41, 124.90, 117.76, 117.59, 116.95, 116.76, 108.23, 104.57, 23.11; HRMS: calculated for C_15_H_11_F_2_N_3_O_2_S [M + H]^+^: 336.06128, found 336.06085.

*Tert-butyl (R)-(4-hydroxy-1-(((7-methyl-5-oxo-5H-thiazolo [3,2-a]pyrimidin-3-yl)methyl)amino)-1-oxobutan-2-yl)carbamate* (**28b**). Yellow oil; yield: 84%; ^1^H NMR (400 MHz, Chloroform-*d*) δ 7.29 (s, 1H), 6.93 (s, 1H), 6.14 (s, 1H), 5.53 (d, *J* = 8.3 Hz, 1H), 4.77 (d, *J* = 5.7 Hz, 2H), 4.35–4.25 (m, 1H), 3.68 (d, *J* = 10.8 Hz, 2H), 2.35 (s, 3H), 2.00–1.69 (m, 2H), 1.25 (s, 9H). MS (ESI) calculated for C_17_H_24_N_4_O_5_S [M + H]^+^, 397; found, 397.

*(S)-2-(7-methyl-5-oxo-5H-thiazolo [3,2-a]pyrimidin-3-yl)-N-(2-oxotetrahydrofuran-3-yl)acetamide* (**34a**). Colorless oil; yield: 46%; ^1^H NMR (400 MHz, DMSO-*d*_6_) δ 8.39 (d, *J* = 7.8 Hz, 1H), 7.21 (s, 1H), 6.01 (s, 1H), 4.50 (q, *J* = 9.3 Hz, 1H), 4.32 (t, *J* = 8.1 Hz, 1H), 4.24–4.16 (m, 1H), 4.12–3.98 (m, 2H), 2.44–2.33 (m, 1H), 2.24 (s, 3H), 2.19–2.08 (m, 1H); ^13^C NMR (101 MHz, DMSO-*d*_6_) δ 175.70, 168.72, 164.34, 163.24, 160.40, 133.25, 110.96, 104.91, 65.78, 48.58, 37.70, 28.63, 23.53; HRMS: calculated for C_13_H_13_N_3_O_4_S [M + H]^+^: 308.06995, found 308.06970.

*Dibenzyl (2-(7-methyl-5-oxo-5H-thiazolo [3,2-a]pyrimidin-3-yl)acetyl)-L-aspartate* (**34b**). White solid; yield: 87%; ^1^H NMR (400 MHz, Chloroform-*d*) δ 7.33 (ddt, *J* = 12.7, 6.2, 3.0 Hz, 10H), 6.76 (s, 1H), 5.99 (s, 1H), 5.09 (dd, *J* = 6.4, 3.7 Hz, 4H), 4.91–4.85 (m, 1H), 4.13–4.07 (m, 2H), 3.04–2.93 (m, 2H), 2.33 (s, 3H). MS (ESI) calculated for C_27_H_25_N_3_O_6_S [M + H]^+^, 520; found, 520.

#### 3.1.19. The Synthesis of **29**

The compound **28b** (39 mg, 0.1 mmol) was dissolved in the solution of 70%TFA/DCM (10 mL) and stirred at room temperature overnight. The solution was concentrated under reduced pressure to obtain the crude product. The residue was purified by semi-preparative HPLC to obtain the desired compound **29**.

*(R)-2-amino-4-hydroxy-N-((7-methyl-5-oxo-5H-thiazolo [3,2-a]pyrimidin-3-yl)methyl)butanamide* (**29**). Colorless oil; yield: 54%; ^1^H NMR (500 MHz, Methanol-*d*_4_) δ 7.24 (s, 1H), 6.20 (s, 1H), 4.94 (d, *J* = 7.2 Hz, 2H), 4.09 (t, *J* = 6.2 Hz, 1H), 3.76 (d, *J* = 4.4 Hz, 2H), 2.38 (s, 3H), 2.13–1.98 (m, 2H); ^13^C NMR (101 MHz, Methanol-*d*_4_) δ 170.05, 166.06, 165.53, 162.89, 136.37, 111.71, 105.81, 59.26, 53.49, 40.13, 34.31, 23.47; HRMS: calculated for C_12_H_16_N_4_O_3_S [M + H]^+^; 297.10159, found 297.10067.

#### 3.1.20. General Procedure for the Synthesis of **31** and **37**

Compounds **26a** or **26c** (0.13 mmol) and Boc-Asp-OtBu (0.2 mmol) were dissolved in DMF (5 mL). Triethylamine (0.38 mmol) was added and the reaction was stirred at 80 °C for 12 h. The reaction was extracted with ethyl acetate and washed with water (30 mL × 2) and brine (30 mL × 2). The organic layer was dried over sodium sulfate and concentrated under reduced pressure to obtain compounds **30** and **36**, which could be used for the next step without purification. Compounds **30** and **36** (0.1 mmol) were dissolved in the solution of 70%TFA/DCM (10 mL) and stirred at room temperature overnight. The solution was concentrated under reduced pressure to obtain the crude product. The residue was purified by semi-preparative HPLC to obtain the desired compounds **31** and **37**.

*(S)-2-amino-4-((7-methyl-5-oxo-5H-thiazolo [3,2-a]pyrimidin-3-yl)methoxy)-4-oxobutanoic acid* (**31**). Colorless oil; yield: 64%; ^1^H NMR (400 MHz, Deuterium Oxide) δ 7.45 (s, 1H), 6.25 (s, 1H), 5.68–5.58 (m, 2H), 4.24 (t, *J* = 5.6 Hz, 1H), 3.16–3.13 (m, 2H), 2.36 (s, 3H); ^13^C NMR (151 MHz, Methanol-*d*_4_) δ 170.40, 170.26, 165.77, 165.76, 162.73, 134.37, 113.64, 105.92, 62.28, 50.41, 35.20, 23.52; HRMS: calculated for C_12_H_13_N_3_O_5_S [M + H]^+^: 312.06487, found 312.06436.

*(S)-2-amino-4-oxo-4-((5-oxo-5H-thiazolo [3,2-a]pyrimidin-7-yl)methoxy)butanoic acid* (**37**). Colorless oil; yield: 77%; ^1^H NMR (400 MHz, Methanol-*d*_4_) δ 8.04 (d, *J* = 4.9 Hz, 1H), 7.46 (d, *J* = 4.9 Hz, 1H), 6.34 (s, 1H), 5.16–5.13 (m, 2H), 4.37 (dd, *J* = 6.3, 4.9 Hz, 1H), 3.24–3.13 (m, 2H); ^13^C NMR (101 MHz, Methanol-*d*_4_) δ 169.23, 168.94, 163.88, 161.23, 159.12, 121.42, 113.19, 100.93, 65.10, 48.87, 33.59; HRMS: calculated for C_11_H_11_N_3_O_5_S [M + H]^+^: 298.04922, found 298.04898.

#### 3.1.21. The Synthesis of **32a**

**26a** (43 mg, 0.2 mmol) and 2-aminopyridine (28 mg, 0.3 mmol) were dissolved in DMF (5 mL). DIEA (77 mg, 0.6 mmol) was added and the reaction was stirred at 90 °C for 12 h. The reaction was extracted with ethyl acetate and washed with water (30 mL × 2) and brine (30 mL × 2). The organic layer was dried over sodium sulfate and separated by silica gel column chromatography to obtain compound **32a**.

*7-methyl-3-((pyridin-2-ylamino)methyl)-5H-thiazolo [3,2-a]pyrimidin-5-one* (**32a**). Colorless oil; yield: 53%; ^1^H NMR (400 MHz, Methanol-*d*_4_) δ 7.98–7.91 (m, 2H), 7.17 (dt, *J* = 8.6, 1.2 Hz, 1H), 6.99–6.93 (m, 2H), 6.16 (d, *J* = 0.7 Hz, 1H), 5.90 (d, *J* = 1.4 Hz, 2H), 2.35 (s, 3H); ^13^C NMR (151 MHz, Methanol-*d*_4_) δ 166.05, 165.79, 162.88, 156.23, 144.10, 139.94, 131.43, 116.78, 114.94, 112.60, 105.84, 53.84, 23.58; HRMS: calculated for C_13_H_12_N_4_OS [M + H]^+^: 273.08046, found 273.08002.

#### 3.1.22. General Procedure for the Synthesis of **33** and **35**

**26a** (43 mg, 0.2 mmol) and H-Asp(OBn)-OBn (84 mg, 0.24 mmol) were dissolved in DMF (5 mL). DIEA (77 mg, 0.6 mmol) was added and the reaction was stirred at 90 °C for 12 h to obtain **32b**, without further purification. The compounds **32b** and **34b** (0.2 mmol) were dissolved in methanol (5 mL). The 10% Pd/C (59 mg) and ammonium formate (0.4 mmol) were added to the solution and stirred at room temperature for 2 h. The reaction was filtered, and the filtrate was concentrated under reduced pressure to obtain the crude product. The residue was purified by semi-preparative HPLC to obtain the desired compounds **33** and **35**.

*((7-methyl-5-oxo-5H-thiazolo [3,2-a]pyrimidin-3-yl)methyl)-L-aspartic acid* (**33**). Colorless oil; yield: 50%; ^1^H NMR (400 MHz, DMSO-*d*_6_) δ 7.08 (s, 1H), 5.96 (s, 1H), 4.14 (d, *J* = 15.5 Hz, 1H), 3.84 (d, *J* = 15.6 Hz, 1H), 2.98 (dd, *J* = 9.5, 3.4 Hz, 1H), 2.30–2.20 (m, 2H), 2.13 (s, 3H); ^13^C NMR (101 MHz, Deuterium Oxide) δ 181.10, 179.63, 165.20, 164.55, 162.86, 136.96, 111.46, 104.78, 59.92, 53.79, 42.45, 22.43. HRMS: calculated for C_12_H_13_N_3_O_5_S [M + H]^+^: 312.06487, found 312.06491.

*(2-(7-methyl-5-oxo-5H-thiazolo [3,2-a]pyrimidin-3-yl)acetyl)-L-aspartic acid* (**35**). Yellowish solid; yield: 48%; m.p.: 253.1–256.8 °C; ^1^H NMR (400 MHz, Deuterium Oxide) δ 7.17 (s, 1H), 6.16 (s, 1H), 4.39 (dd, *J* = 8.3, 4.5 Hz, 1H), 4.19 (q, *J* = 16.7 Hz, 2H), 2.67–2.49 (m, 2H), 2.33 (s, 3H); ^13^C NMR (101 MHz, Deuterium Oxide) δ 178.87, 178.57, 170.89, 167.36, 164.86, 162.91, 132.25, 112.37, 104.58, 53.18, 39.72, 37.80, 22.45; HRMS: calculated for C_13_H_13_N_3_O_6_S [M + H]^+^: 340.05978, found 340.05933.

#### 3.1.23. The Synthesis of **38**

4-bromo-6-chloropyridazin-3-amine (832 mg, 4 mmol) was slightly dissolved in isopropyl alcohol (10 mL). 40% chloro-acetaldehyde solution (1.88 mL, 9.6 mmol) was added and the reaction was heated to 90 °C overnight. The reaction was extracted with ethyl acetate and washed with water (30 mL × 2) and brine (30 mL × 2). The organic layer was dried over sodium sulfate and separated by silica gel column chromatography to obtain compound **38**.

*8-bromo-6-chloroimidazo [1,2-b]pyridazine* (**38**). Brown oil; yield: 65%; ^1^H NMR (500 MHz, Chloroform-*d*) δ 8.05 (d, *J* = 8.1 Hz, 1H), 7.89 (d, *J* = 3.9 Hz, 1H), 7.33 (s, 1H). MS (ESI) calculated for C_6_H_3_BrClN_3_ [M + H]^+^, 232; found, 232.

#### 3.1.24. The Synthesis of **39**

**38** (590 mg, 3.15 mmol) was dissolved in DMF (10 mL). NIS (781 mg, 3.47 mmol) and acetic acid (207 μL, 1.5 mmol) were added and the reaction was heated to 65 °C overnight. The reaction was extracted with ethyl acetate and washed with water (30 mL × 2) and brine (30 mL × 2). The organic layer was dried over sodium sulfate and separated by silica gel column chromatography to obtain compound **39**.

*8-bromo-6-chloro-3-iodoimidazo [1,2-b]pyridazine* (**39**). Yellow solid; yield: 56%; ^1^H NMR (500 MHz, Chloroform-*d*) δ 7.74–7.66 (m, 1H), 7.14–7.01 (m, 1H). MS (ESI) calculated for C_6_H_2_BrClIN_3_ [M + H]^+^, 358; found, 358.

#### 3.1.25. The Synthesis of **40**

Compound **39** (628 mg, 1.76 mmol), 1-(tert-butyl) 2-methyl (*2R*,*4S*)-4-hydroxypyrrolidine-1,2-dicarboxylate (736 mg, 3 mmol), K_2_CO_3_ (1.1 g, 8 mmol) and KI (332 mg, 2 mmol) were dissolved in DMF (10 mL), and the reaction was heated to 90 °C for 36 h. The reaction was extracted with ethyl acetate and washed with water (30 mL × 2) and brine (30 mL × 2). The organic layer was dried over sodium sulfate and separated by silica gel column chromatography to obtain compound **40**.

*1-(tert-butyl) 2-methyl (2R,4S)-4-((6-chloro-3-iodoimidazo [1,2-b]pyridazin-8-yl)oxy)pyrrolidine-1,2-dicarboxylate* (**40**). Yellowish solid; yield: 57%; ^1^H NMR (500 MHz, DMSO-*d*_6_) δ 7.80 (s, 1H), 7.11 (s, 1H), 5.54 (s, 1H), 4.41–4.31 (m, 1H), 3.72 (t, *J* = 17.1 Hz, 5H), 2.67–2.65 (m, 1H), 2.38–2.35 (m, 1H), 1.37 (s, 9H). MS (ESI) calculated for C_17_H_20_ClIN_4_O_5_ [M + H]^+^, 523; found, 523.

#### 3.1.26. The Synthesis of **41**

Compound 40 (373 mg, 0.71 mmol), and (3,4-dimethoxyphenyl)boronic acid (160 mg, 0.88 mmol) and Na_2_CO_3_ (110 mg, 1.04 mmol) in the solution of dioxane: water = 4:1 (10 mL) were placed in a three-necked flask under nitrogen. Pd(dppf)Cl_2_ (30 mg, 0.04 mmol) was added and the mixture was stirred at 95 °C for 12 h. The reaction was concentrated under reduced pressure. The product was extracted with EA and washed with water (30 mL × 2) and brine (30 mL × 2). The organic layer was dried over sodium sulfate and separated by silica gel column chromatography to obtain compound **41**.

*1-(tert-butyl)-2-methyl-(2R,4S)-4-((6-chloro-3-(3,4-dimethoxyphenyl)imidazo [1,2-b]pyridazin-8-yl)oxy)pyrrolidine-1,2-dicarboxylate* (**41**). Yellow oil; yield: 50%; ^1^H NMR (500 MHz, DMSO-d_6_) δ 8.25 (s, 1H), 7.69 (s, 1H), 7.01 (s, 1H), 5.54 (s, 1H), 4.37 (q, J = 9.6, 8.3 Hz, 1H), 3.82–3.64 (m, 7H), 2.73–2.62 (m, 1H), 2.41–2.32 (m, 1H), 1.53–1.14 (m, 15H); ^13^C NMR (101 MHz, Methanol-d_4_) δ 174.72, 155.42, 154.20, 150.71, 150.29, 149.13, 134.88, 130.95, 130.90, 121.87, 121.02, 112.77, 111.63, 100.13, 82.15, 79.25, 59.28, 56.50, 56.42, 52.93, 52.90, 37.27, 28.49; HRMS: calculated for C_25_H_29_N_4_O_7_Cl [M + H]^+^: 533.17975, found 533.17944.

#### 3.1.27. The Synthesis of **42**

Compound **41** (177 mg, 0.33 mmol), and methyl-boronic acid (40 mg, 0.66 mmol) and K_3_PO_4_ (282 mg, 1.33 mmol) were dissolved in dry dioxane (10 mL) under nitrogen and the mixture was stirred at room temperature for 10 min. S-Phos (54 mg, 0.13 mmol) and Pd(OAc)_2_ (11 mg, 0.07 mmol) were added and the reaction was heated to 150 °C for 4 h. The reaction was concentrated under reduced pressure. The product was extracted with EA and washed with water (10 mL × 2) and brine (10 mL × 2). The organic layer was dried over sodium sulfate and separated by silica gel column chromatography to obtain compound **42**.

*1-(tert-butyl)-2-methyl-(2R,4S)-4-((3-(3,4-dimethoxyphenyl)-6-methylimidazo [1,2-b]pyridazin-8-yl)oxy)pyrrolidine-1,2-dicarboxylate* (**42**). Yellowish solid; yield: 32%; ^1^H NMR (500 MHz, DMSO-d_6_) δ 7.99 (s, 1H), 7.75 (s, 1H), 7.11 (d, J = 8.9 Hz, 1H), 6.76 (s, 1H), 5.49 (s, 1H), 4.43–4.36 (m, 1H), 3.87–3.71 (m, 12H), 2.71–2.62 (m, 1H), 2.56 (s, 3H), 2.43–2.32 (m, 1H), 1.411.43–1.38 (m, 9H). MS (ESI) calculated for C_26_H_32_N_4_O_7_ [M + H]^+^, 513; found, 513.

#### 3.1.28. The Synthesis of **44**

Compound **42** (108 mg, 0.21 mmol) was dissolved in THF (5 mL). LiOH (151 mg, 6.3 mmol) in water (5 mL) was added, and the reaction was stirred at room temperature overnight. The solution was concentrated under reduced pressure to obtain the crude product. The residue was purified by semi-preparative HPLC to obtain the desired compound **44**.

*(2R,4S)-1-(tert-butoxycarbonyl)-4-((3-(3,4-dimethoxyphenyl)-6-methylimidazo [1,2-b]pyridazin-8-yl)oxy)pyrrolidine-2-carboxylic acid* (**44**). Colorless oil; yield: 70%; ^1^H NMR (500 MHz, Methanol-d_4_) δ 7.80 (s, 1H), 7.73 (s, 1H), 7.62 (d, J = 8.3 Hz, 1H), 7.03 (d, J = 8.5 Hz, 1H), 6.62 (s, 1H), 4.52 (t, J = 7.9 Hz, 1H), 3.89 (d, J = 8.9 Hz, 9H), 2.77 (dd, J = 13.8, 7.5 Hz, 1H), 2.46–2.35 (m, 1H), 1.99 (s, 3H), 1.43 (s, 9H). MS (ESI) calculated for C_25_H_30_N_4_O_7_ [M + H]^+^, 499; found, 499.

#### 3.1.29. General Procedure for the Synthesis of **43** and **45**

The compounds **42** and **44** (0.1 mmol) were dissolved in the solution of 70%TFA/DCM (10 mL) and stirred at room temperature overnight. The solution was concentrated under reduced pressure to obtain the crude product. The residue was purified by the semi-preparative HPLC to obtain the desired compounds **43** and **45**.

*Methyl (2R,4S)-4-((3-(3,4-dimethoxyphenyl)-6-methylimidazo [1,2-b]pyridazin-8-yl)oxy)pyrrolidine-2-carboxylate* (**43**). White solid; yield: 43%; m.p.: 127.2–129.6 °C; ^1^H NMR (600 MHz, Methanol-*d*_4_) δ 8.19 (s, 1H), 7.67 (d, *J* = 7.7 Hz, 2H), 7.13–7.09 (m, 2H), 5.68 (s, 1H), 3.91–3.87 (m, 13H), 2.91 (dd, *J* = 14.8, 7.7 Hz, 1H), 2.68 (s, 3H), 2.67–2.63 (m, 1H); ^13^C NMR (151 MHz, Methanol-*d*_4_) δ 169.74, 158.18, 151.57, 150.62, 150.52, 133.20, 131.66, 123.97, 122.14, 120.49, 112.95, 112.61, 103.80, 79.56, 59.48, 56.66, 56.52, 54.17, 51.99, 35.57, 22.44; HRMS: calculated for C_21_H_24_N_4_O_5_ [M + H]^+^: 413.18195, found 413.18176.

*(2R,4S)-4-((3-(3,4-dimethoxyphenyl)-6-methylimidazo [1,2-b]pyridazin-8-yl)oxy)pyrrolidine-2-carboxylic acid* (**45**). Colorless oil; yield: 57%; ^1^H NMR (600 MHz, DMSO-*d*_6_) δ 8.08 (s, 1H), 7.76–7.71 (m, 2H), 7.09 (d, *J* = 8.5 Hz, 1H), 6.84 (s, 1H), 5.62 (t, *J* = 4.3 Hz, 1H), 4.60 (dd, *J* = 10.5, 7.7 Hz, 1H), 3.85 (s, 3H), 3.82 (s, 3H), 3.59 (d, *J* = 13.5 Hz, 1H), 2.66 (dd, *J* = 14.4, 7.5 Hz, 1H), 2.56 (s, 5H); ^13^C NMR (151 MHz, DMSO-*d*_6_) δ 169.52, 153.60, 150.98, 148.64, 133.21, 129.07, 128.03, 120.99, 119.17, 115.37, 111.90, 110.38, 99.97, 77.33, 58.07, 55.59, 55.57, 50.60, 34.30, 21.92; HRMS: calculated for C_20_H_22_N_4_O_5_ [M + H]^+^: 399.16630, found 399.16580.

### 3.2. Biology

#### 3.2.1. Reagents

The following reagents and antibodies were purchased for the study of the biological activity of the compounds: anti-COL1A1 antibody (ET1609-68) from HUABIO (Hangzhou, China); anti-fibronectin (ab2413), anti-TGFβ1 (ab179695), anti-Timp1 (ab211926) antibodies from Abnova (Boulder, CO, USA) and Abcam (Cambridge, UK); anti-GAPDH antibody (#5174), anti-IKKβ antibody (#2370), anti-P-IKKβ antibody (#2697), anti-NF-κB p65 antibody (#8242), anti-P-NF-κB p65 antibody (#3033) and anti-IL-6 antibody (#12153) from Cell Signaling Technology (Danvers, MA, USA), recombinant Human TGFβ1 Protein (240-B) from R & D Systems (Minneapolis, MN, USA), and LPS (L2880) from Sigma (Olney, MD, USA).

#### 3.2.2. Cell Culture

Human hepatic stellate cell LX-2 was purchased from Chinese National Infrastructure of Cell Line Resource (NICR, 4201HUM-CCTCC00664). The LX-2 cells were cultured in DMEM medium (BasalMedia, L431121, Shanghai, China) supplemented with 10% fetal bovine serum (Gibco, 2440093, Paisley, UK), 1% GlutaMAX additive (Gibco, 350050061, New York, NY, USA) and 1% 100 U/mL penicillin-100 mg/mL streptomycin (Gibco, #15140–122, New York, NY, USA) at 37 °C in a 5% CO_2_ environment. Cells were verified to be free of mycoplasma contamination.

#### 3.2.3. COL1A1 Promoter Inhibition Rate Assay

The LX-2 cells at 90% confluent were transfected with the plasmid of pGL4.17-COL1A1 promoter using Lipofectamine 2000 (Invitrogen, 11668019, Carlsbad, CA, USA) with Opti-MEM serum-free medium (Gibco, 1930104). After culturing for 6 h, the cells were replaced with a complete medium and seeded in 96-well plates for 24 h. The compound to be tested was prepared using a complete medium and added to the 96-well plate. After 24 h, Bright-Glo Luciferase Assay System (Promega, Madison, WI, USA) was used to detect the activity of COL1A1 promoter and the assaying inhibition rate.

#### 3.2.4. Sulfor-Hodamine B (SRB) Assay

The LX-2 cells were seeded in a 96-well plate when reaching 90% confluent. After 24 h, the compound to be tested was prepared using a complete medium and added to the 96-well plate for 24 h. After washing with PBS, the cells were fixed with 10% (*wt*/*vol*) trichloroacetic acid for 1 h, washed with deionized water and dried completely and, after staining with SRB for 20 min, the excess dye was washed with 1% acetic acid (*vol*/*vol*) three times. The dye bound to the protein was dissolved with 10 mM Tris base solution and OD values were determined at 510 nmol/L.

#### 3.2.5. RT–qPCR Assay

The LX-2 cells were seeded in a 6-well plate and cultured in a complete medium at 37 °C in a 5% CO_2_ environment until reaching 90% confluence. After culture without serum medium for 24 h, the cells were treated with 2 ng/mL TGFβ1 or 1 μg/mL LPS and the right concentration of compounds for 24 h. Total RNA was extracted using TRIzol reagent and purified by NucleoSpin RNA Clean-up Kit (Macherey-Nagel, Munich, Germany). Total RNA was reverse-transcribed into cRNAs using a Transcriptor First Strand cDNA Synthesis Kit (Roche, Basel, Switzerland). The relative expression levels of the target gene were determined using a TaqMan probe with an ABI Q3 quantitative real-time PCR system, while GAPDH was utilized as an internal control for mRNA expression. The fold change of target mRNA expression was calculated using Equation 2^−∆∆Ct^.

#### 3.2.6. Western Blot

After the confluence of LX-2 cells reached 90%, the cells were treated with serum-free medium for 24 h. 2 ng/mL TGFβ1 or 1 μg/mL LPS were used in combination with compounds of different concentrations for 24 h. The samples of cells were lysed in RIPA buffer supplemented with protease inhibitor cocktail (Roche) and separated by SDS-PAGE. After the proteins were transferred to the PVDF membrane, 5% skim milk in TBS-T buffer was used to block them at room temperature for 2 h. Specific primary and secondary antibodies were used to detect the target protein. Finally, the blot was visualized using the Tanon 5200 system (Tanon, Shanghai, China) and the protein expression of GAPDH was used as the internal reference.

#### 3.2.7. Statistics

All experiments were repeated at least three times. The experimental data were expressed as mean ± SD and were analyzed by one-way analysis of variance (ANOVA). *p* valve < 0.05 was considered statistically significant.

## 4. Conclusions

The complex pathogenesis of liver fibrosis, combined with the scarcity of effective clinical treatments, underscores the need for novel therapeutic strategies. In this study, L-aspartic acid, a compound with limited anti-liver fibrosis activity, was selected as the lead structure, and its functional groups were systematically modified. A total of 32 target compounds were synthesized, including 22 novel entities not previously reported. Initial screening for in vitro anti-fibrotic activity was conducted using the COL1A1 promoter model, where four compounds (**3**, **9**, **41** and **45**) demonstrated markedly higher inhibitory activity compared to aspartic acid (5.9–8.6-fold) and EGCG (1.8–2.7-fold).

SAR analysis identified the dominant scaffold as follows: the 1-carboxyl group linked to 4-fluorobenzyl via an ester bond, the 2-amino group conjugated to bile acid derivatives through an amide bond, and the 4-carboxyl group attached to aliphatic hydrocarbons. Additionally, a novel scaffold was identified: 6-chloro-3-phenylimidazo[1,2-b]pyridazine, characterized by a poorly water-soluble saturated heterocycle connected via an ether bond. This discovery is highly significant for the identification of novel targets and structures in anti-liver fibrosis drug development. However, it must be acknowledged that compound **41** exhibits certain structural challenges: its molecular weight (MW) exceeds 500, the number of nitrogen or oxygen atoms (NorO) surpasses 10, and the number of rotatable bonds (Rotors) is greater than 7, which compromises its potential as an ideal small-molecule drug. Nevertheless, these limitations also present considerable opportunities for structural optimization. We have confirmed that the core scaffold possesses anti-liver fibrosis activity and have gained valuable insights into structural modifications. Future research can focus on using this core structure as a lead compound for further modifications, providing a broader array of active molecules for the development of anti-liver fibrosis therapeutics.

The effects of the synthesized compounds on liver fibrosis-associated protein expression were assessed by western blot analysis. Compound **41** notably inhibited the expression of fibronectin, COL1A1, and α-SMA in TGFβ1-induced LX-2 cells and demonstrated safety in LX-2 cells with an SI > 10. In an LPS-induced inflammation model using LX-2 cells, compounds **41** and **8a** were able to inhibit LX-2 cell activation, significantly reducing the expression of COL1A1, fibronectin, α-SMA, and CTGF in a dose-dependent manner. Mechanistic studies suggested that compounds **41** and **8a** may attenuate hepatic fibrosis progression by inhibiting the IKKβ-NF-κB signaling pathway, thereby suppressing the inflammatory response.

Compound **41** exhibited enhanced anti-liver fibrosis activity at both cellular and molecular levels, highlighting its potential as a promising candidate for further investigation as an anti-fibrotic lead compound. This study provides a foundational basis for future research and offers valuable insights for the development of novel liver fibrosis therapeutics. Given the pivotal role of the IKKβ-NF-κB signaling pathway in inflammatory responses, compound **41** holds significant promise for drug discovery efforts targeting a range of inflammatory and fibrotic diseases.

## Figures and Tables

**Table 1:** The inhibition of COL1A1 promotor of target compounds.

Compound	Inhibitory Rate (%)	Compound	Inhibitory Rate (%)
**1**	/ ^a^	**11f**	20.62 ± 14.30
**3**	81.54 ± 5.62	**20a**	17.91 ± 10.07
**4a**	14.92 ± 8.45	**20b**	24.02 ± 6.38
**4b**	31.18 ± 14.30	**23a**	27.88 ± 1.16
**4c**	35.54 ± 18.11	**23b**	34.60 ± 1.75
**4d**	33.14 ± 19.03	**28a**	43.91 ± 6.36
**5**	23.98 ± 15.44	**29**	33.20 ± 33.32
**6**	/	**31**	14.63 ± 3.11
**8a**	49.34 ± 13.28	**32a**	47.69 ± 4.81
**8b**	23.82 ± 4.57	**33**	6.39 ± 7.06
**9**	97.44 ± 3.53	**34a**	15.46 ± 16.61
**11a**	11.42 ± 18.53	**35**	/
**11b**	/	**37**	8.46 ± 8.16
**11c**	/	**41**	66.72 ± 1.96
**11d**	19.85 ± 4.99	**43**	38.23 ± 3.28
**11e**	27.93 ± 5.81	**45**	69.64 ± 8.48
L-Asp	11.33 ± 0.35	EGCG	36.46 ± 4.64

^a^ No activity. Inhibitory rate (%) = (Control RFU, Compound RFU)/Control RFU × 100%. The RFU results are presented in Table S1 and Figure S1.

**Table 2:** Inhibition effects on COL1A1 promoter of representative compounds.

Code	IC_50_ (μmol/L) ^a^	CC_50_ (μmol/L) ^b^	SI ^c^
**3**	327.1 ± 17.1	659.7 ± 24.61	2.01
**9**	269.9 ± 47.9	351.0 ± 19.4	1.30
**41**	30.6 ± 0.3	510.5 ± 51.5	16.68
**45**	297.9 ± 51.7	632.2 ± 11.2	2.12
**8a**	1054.4 ± 111.2	13,871.0 ± 968.7	13.16

^a^ Half maximal inhibition concentration to inhibit the activity of COL1A1 promoter by 50%. ^b^ Cytotoxic concentration required to inhibit the growth of LX-2 cells by 50%. SRB data for five compounds are shown in Figure S2. ^c^ Selectivity index (CC_50_/IC_50_).

## Data Availability

The original contributions presented in the study are included in the article and Supplementary Material, further inquiries can be directed to the corresponding authors.

## Supplementary Materials

The following supporting information can be downloaded at: https://www.mdpi.com/article/10.3390/molecules29194774/s1, Figure S1: The RFU of COL1A1 promotor of target compounds; Figure S2: Sulforhodamine B (SRB) assay results of the target compound; Figure S3: Compounds inhibit the expression of inflammation-related proteins; Figure S4: Compounds inhibit the expression of LPS-induced fibrogenic proteins in LX-2 cells; Table S1: The relative fluorescence unit (RFU) of COL1A1 promotor of target compounds. ^1^H-NMR, ^13^C-NMR, and HRMS spectra for the target compounds.

## Abbreviations

ECM, extracellular matrix; NASH, non-alcoholic steato hepatitis; HSC, hepatic stellate cell; IKK, IκB kinase; MAFLD, metabolic-associated fatty liver disease; COL1A1, Collagen Type I Alpha 1; EGCG, epigallocatechin gallate; HCV, Hepatitis C Virus; TGFβ, Transforming Growth Factor Beta; α-SMA, Alpha Smooth Muscle Actin; CTGF, Connective Tissue Growth Factor; TIMP1, Tissue Inhibitor of Metalloproteinase 1; MMP2, Matrix Metalloproteinase 2; GAPDH, Glyceraldehyde 3-Phosphate Dehydrogenase.
