# Supplementary material for: Synthesis and Anti-Liver Fibrosis Research of Aspartic Acid Derivatives
Source: Molecules. 2024 Oct 9;29(19):4774. doi: 10.3390/molecules29194774 (PMC11477965; doi:10.3390/molecules29194774)
Supplement: Supplementary file 1 [file molecules-29-04774-s001.zip › molecules-3194077-supplementary.pdf]

SUPPORTING INFORMATION

Synthesis and anti-liver fibrosis activity research of L-aspartic acid derivatives

Miao Lv [a, ‡], Si-Min Guo [a, ‡], He-Xian Yang [a], Yong-Jian Wang [a], Yi-Ming Li [a], Yang Li [a], Hong Yi [a], Hong-Wei He [a, *], Zhuo-Rong Li [a, *]

[a] *Institute of Medicinal Biotechnology, Chinese Academy of Medical Sciences & Peking Union Medical College, Beijing 100050, China*

[*]Corresponding authors.

[*]Zhuo-Rong Li. Tel: +86-10-63027185; fax: +86-10-63017302. [*]Hong-Wei He. Tel: +86-10- 63010985; fax: +86-10-63017302.

E-mail addresses: lizhuorong@imb.pumc.edu.cn (Zhuo-Rong Li), hehwei@imb.pumc.edu.cn (Hong-Wei He).

[‡]Miao Lv and Simin Guo contributed equally to this work.

Contents of Supporting Information

Supplementary Table S1 1

Supplementary Figure S1 1

Supplementary Figure S2 2

Supplementary Figure S3 2

Supplementary Figure S4 3

^{1}H and ^{13}C NMR and HRMS spectra of all target compounds....... 4-48

HPLC traces for all target compounds... 49-64

Supplementary Table S1. The relative fluorescence unit (RFU) of COL1A1 promotor of target compounds. The values were presented as the mean ± SD, n = 3.

Compound	RFU	Compound	RFU
**1**	143674.9 ± 32109.7	**11f**	99123.6 ± 17860.3
**3**	23056.9 ± 7017.3	**20a**	102508.7 ± 12580.9
**4a**	106237.2 ± 10544.5	**20b**	94881.8 ± 7963.9
**4b**	85934.6 ± 17864.6	**23a**	90057.5 ± 1452.6
**4c**	80485.7 ± 22615.1	**23b**	81660.6 ± 2179.5
**4d**	83497.3 ±23755.3	**28a**	70040.5 ± 7951.7
**5**	94919.5 ± 19273.7	**29**	83406.5 ± 41616.9
**6**	128613.0 ± 57574.1	**31**	106608.6 ± 3888.3
**8a**	63265.3 ± 16582.4	**32a**	65321.0 ± 6005.7
**8b**	95132.4 ± 5710.5	**33**	116893.2 ± 8812.8
**9**	3188.3 ±4407.2	**34a**	105579.0 ± 20740.0
**11a**	110620.0 ± 23148.4	**35**	161478.3 ± 67708.9
**11b**	130725.3 ± 8310.2	**37**	114307.8 ± 10191.8
**11c**	158140.3 ± 50740.1	**41**	41555.6 ± 2445.9
**11d**	100087.5 ± 6229.2	**43**	77132.5 ± 4101.8
**11e**	89998.6 ± 7257.5	**45**	37918.9 ± 10579.6
L-Asp	110724.3 ± 444.6	EGCG	79340.1 ± 5808.7
Control	124874.7 ± 10750.9		

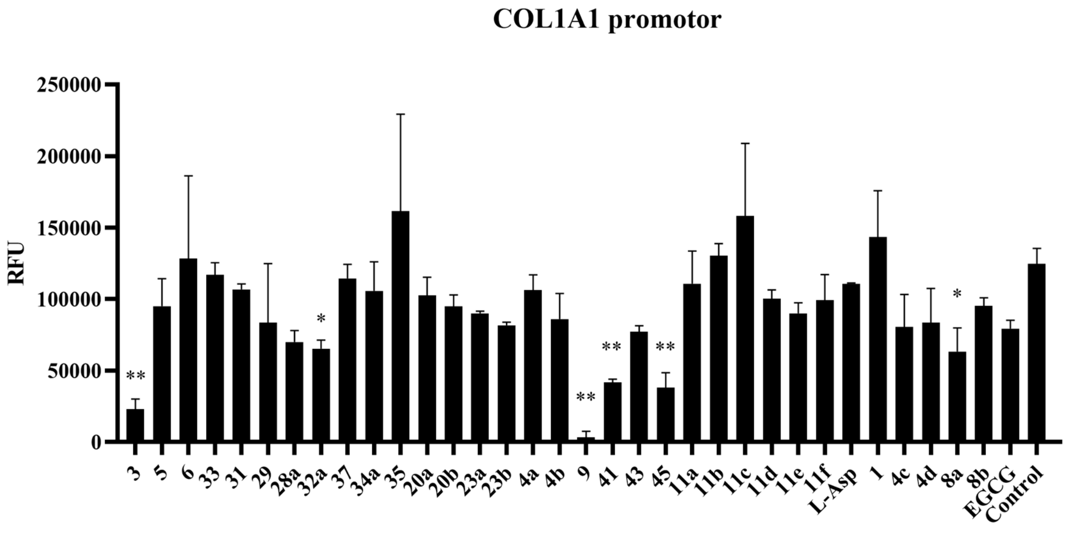

Supplementary Figure S1. The RFU of COL1A1 promotor of target compounds. n=3, *P <0.05, **P < 0.01 vs. Control group.

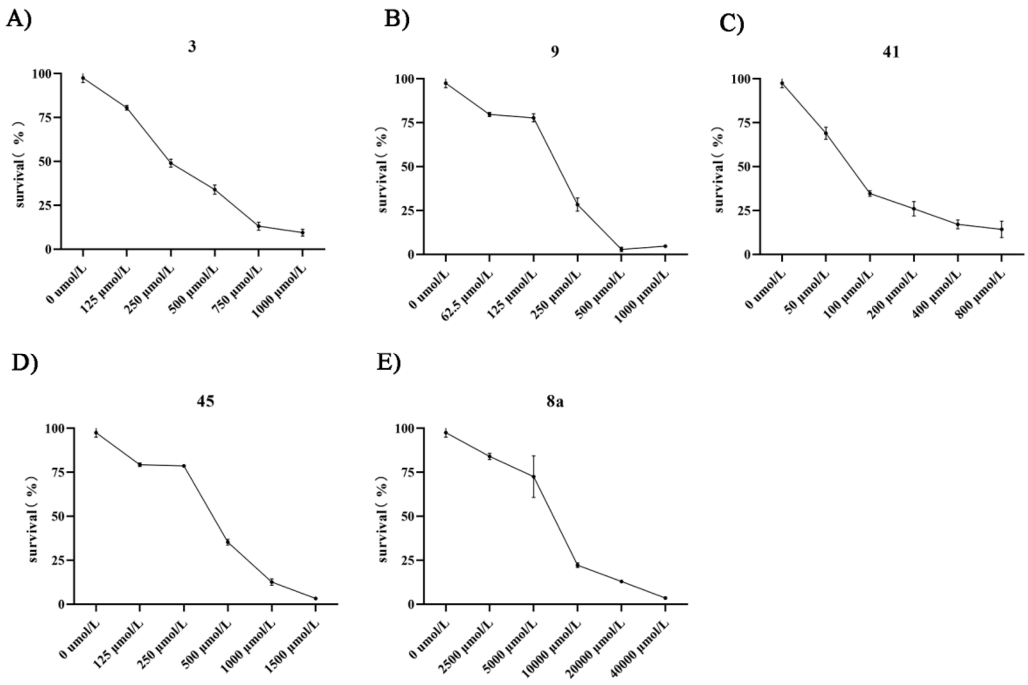

Supplementary Figure S2. (A-E) Sulforhodamine B (SRB) assay results of the target compound.

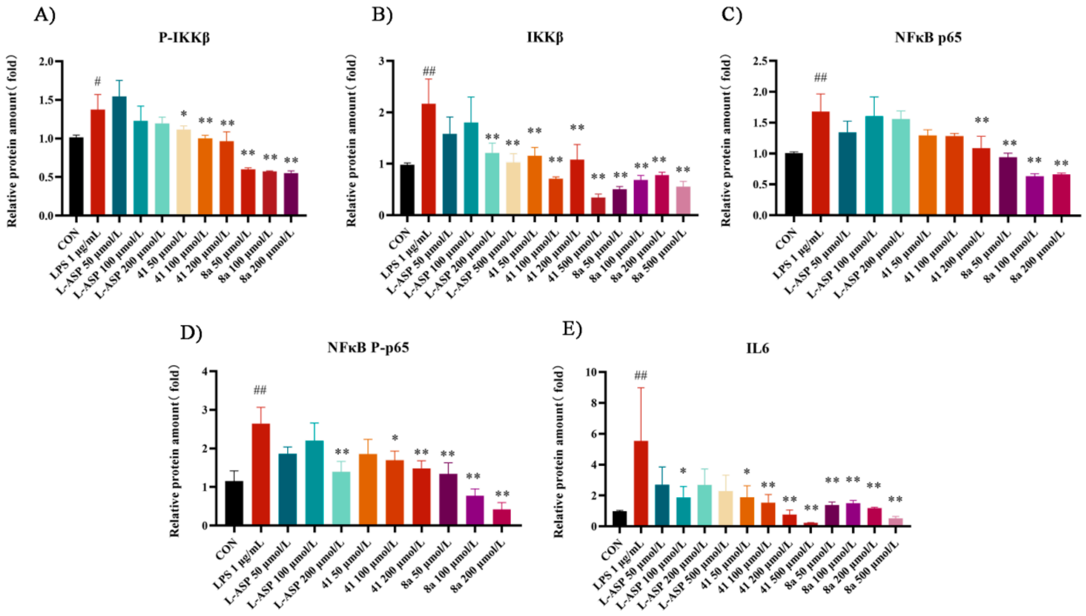

Supplementary Figure S3. (A-E) Compounds inhibit the expression of inflammation-related proteins. The values are expressed as the mean ± SD of three independent assays, $^{\#}P < 0.05$, $^{\#\#}P < 0.01$, significantly different from the non-treated control, and $^{*}P < 0.05$, $^{**}P < 0.01$ significantly different from the LPS treatment group.

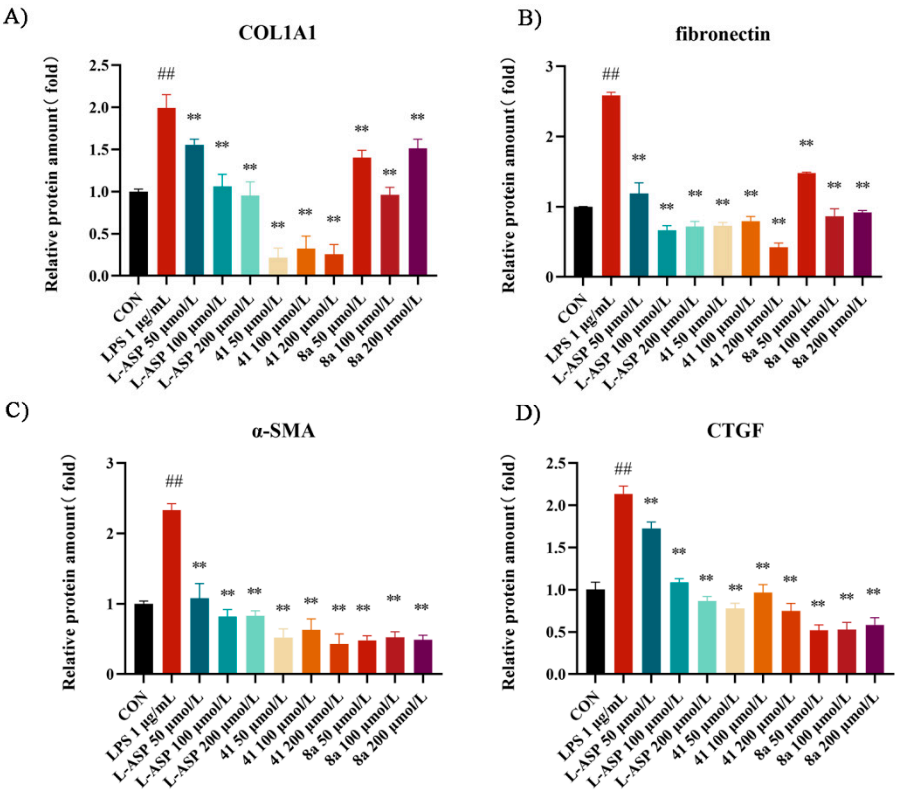

Supplementary Figure S4. (A-D) Compounds inhibit the expression of LPS-induced fibrogenic proteins in LX-2 cells. The values are expressed as the mean ± SD of three independent assays, $^{\#}$P < 0.05, $^{\#\#}$P < 0. 01, significantly different from the non-treated control, and *P < 0.05, **P < 0. 01 significantly different from the LPS treatment group.

^{1}H and ^{13}C NMR and HRMS spectra of all target compounds.

Compound 1: ^{1}H and ^{13}C NMR and HRMS spectra.

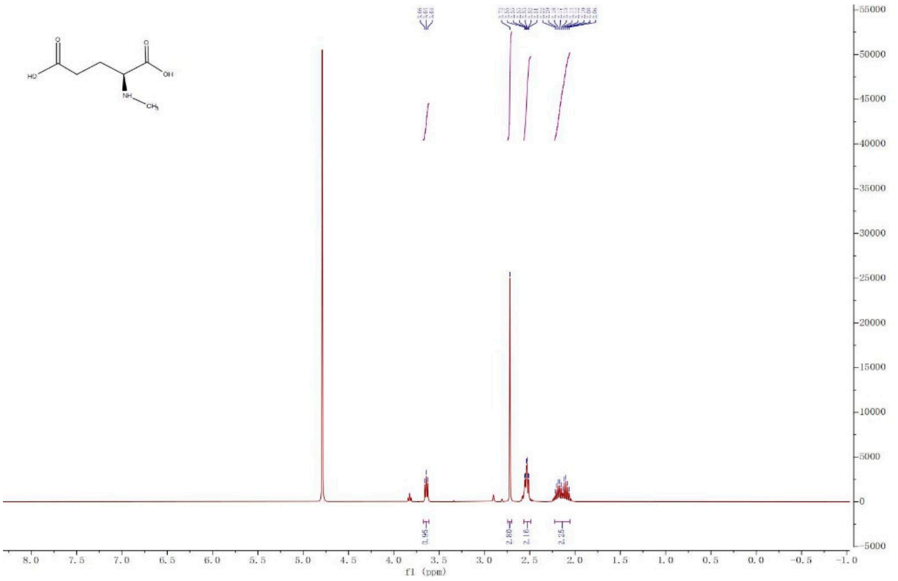

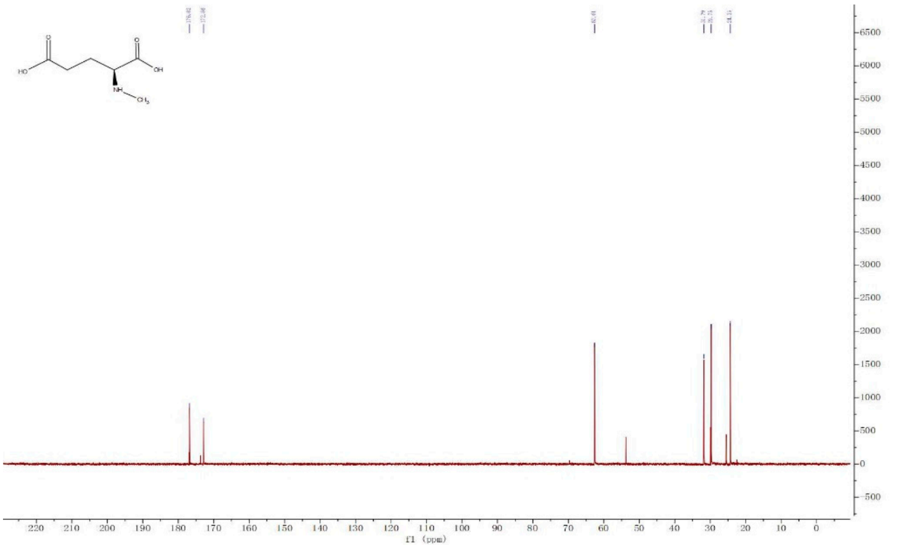

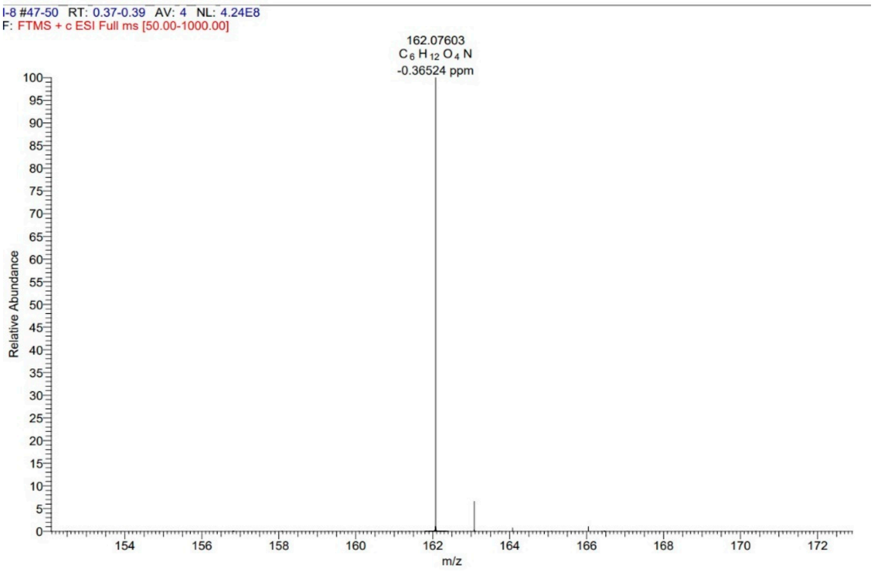

Compound 3: ^{1}H and ^{13}C NMR and HRMS spectra.

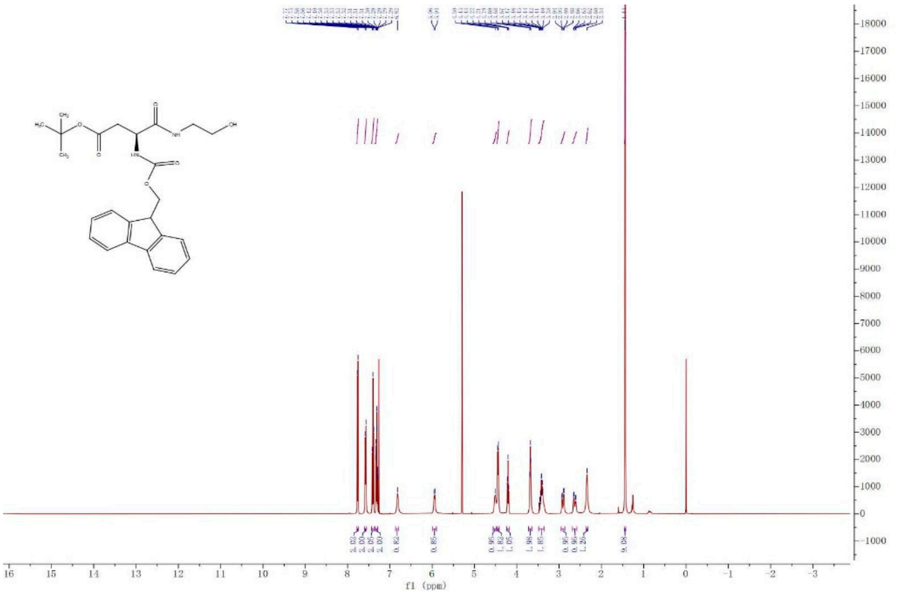

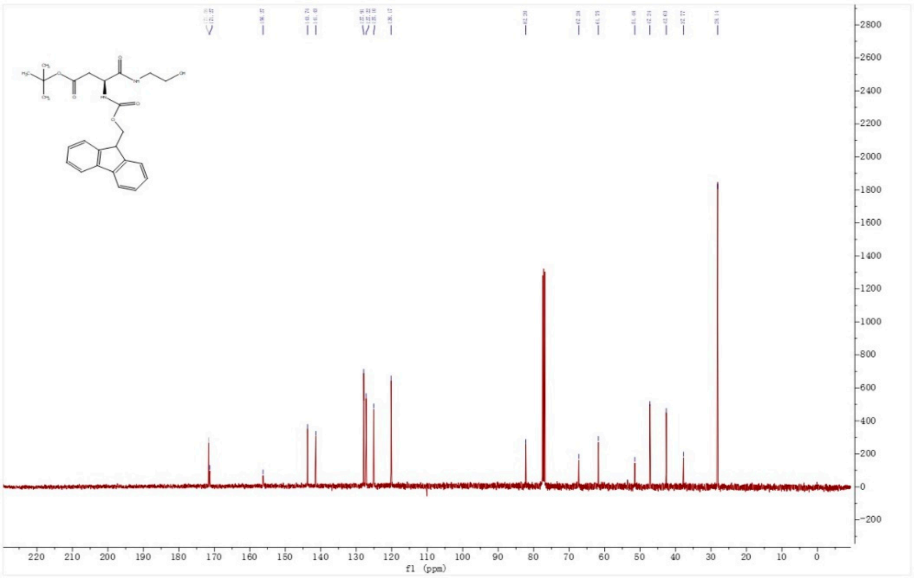

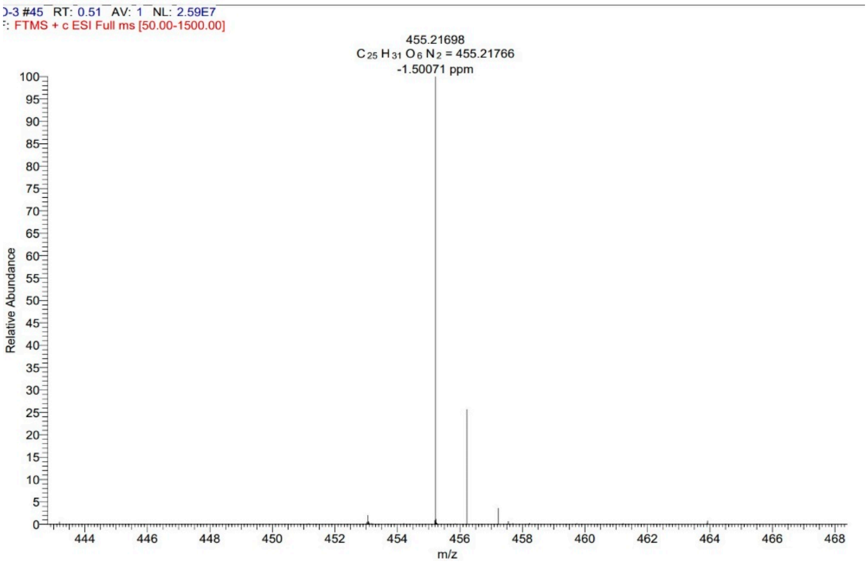

Compound 4a: ^{1}H and ^{13}C NMR and HRMS spectra.

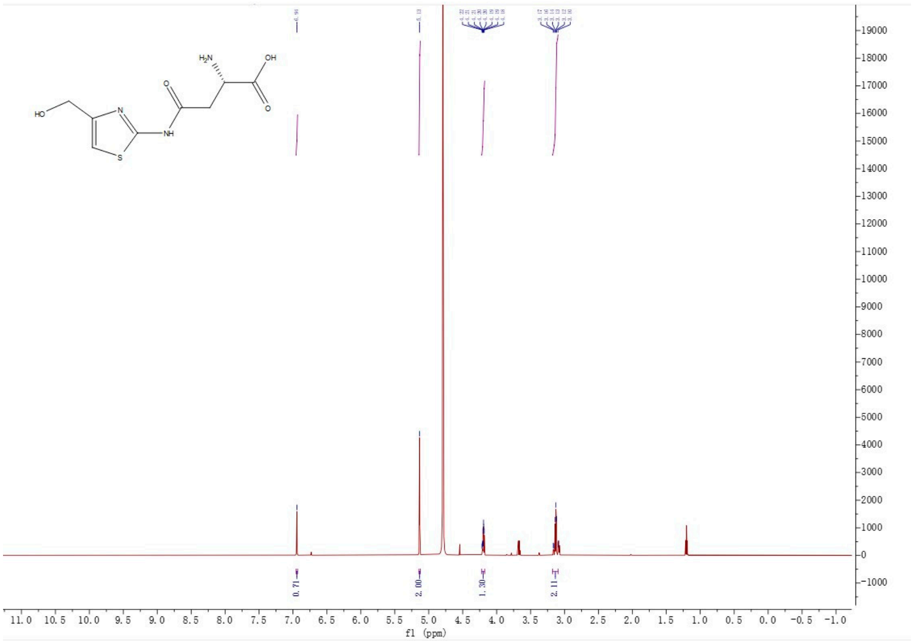

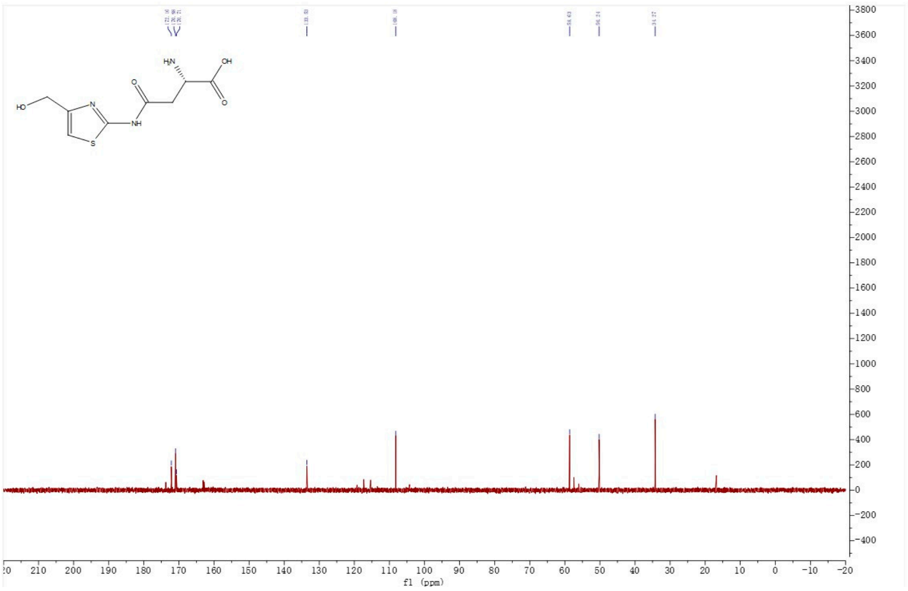

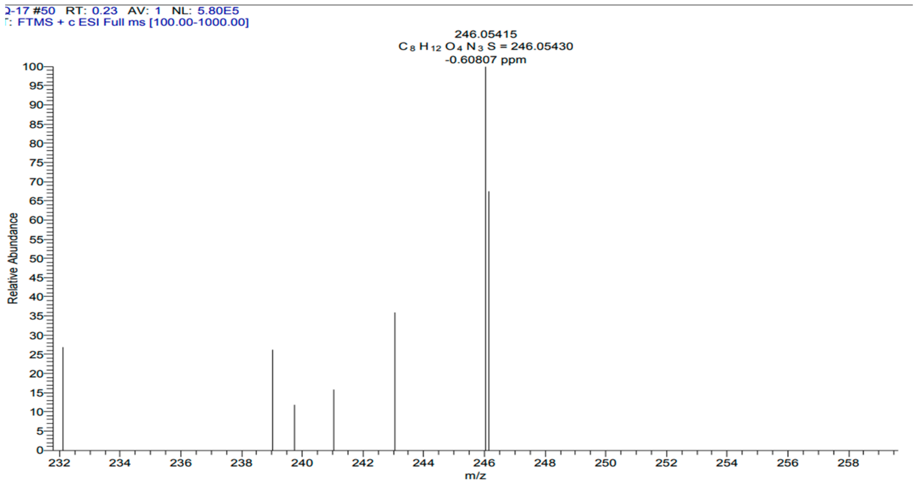

Compound 4b: ^{1}H and ^{13}C NMR and HRMS spectra.

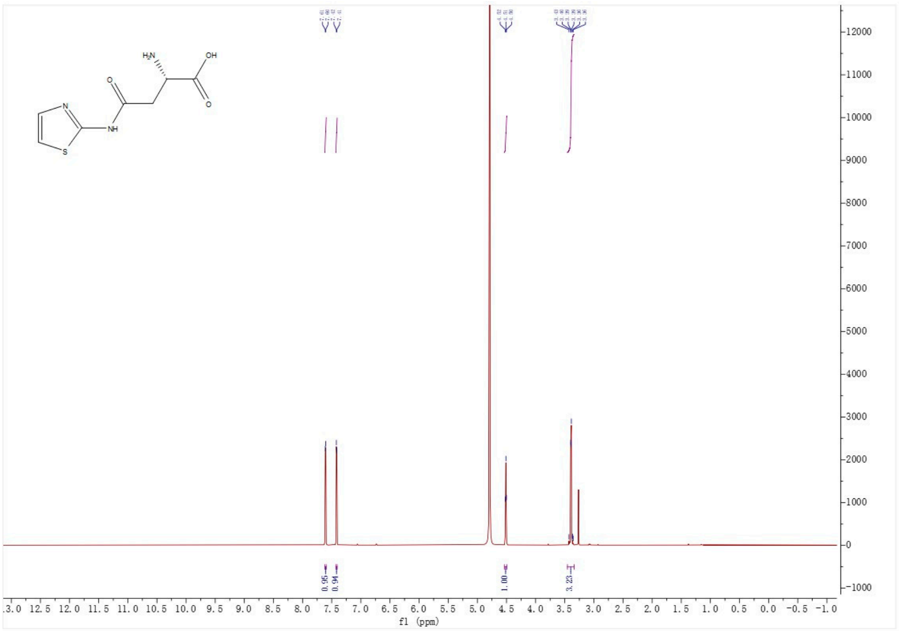
H2N
OH
O
O
N
NH
S
0.95
0.94
1.00
3.23
f1 (ppm)

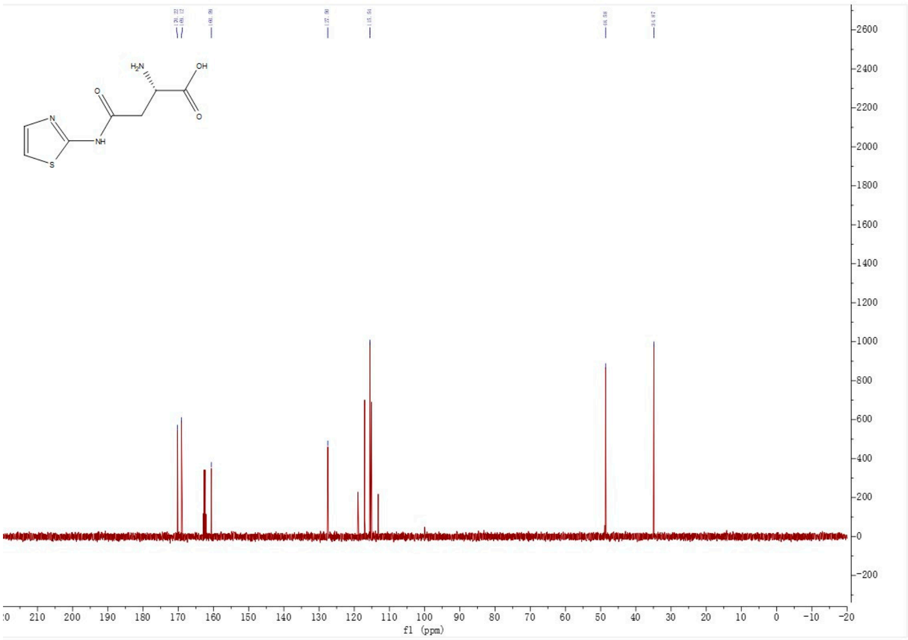
H2N
OH
O
O
N
NH
S
f1 (ppm)

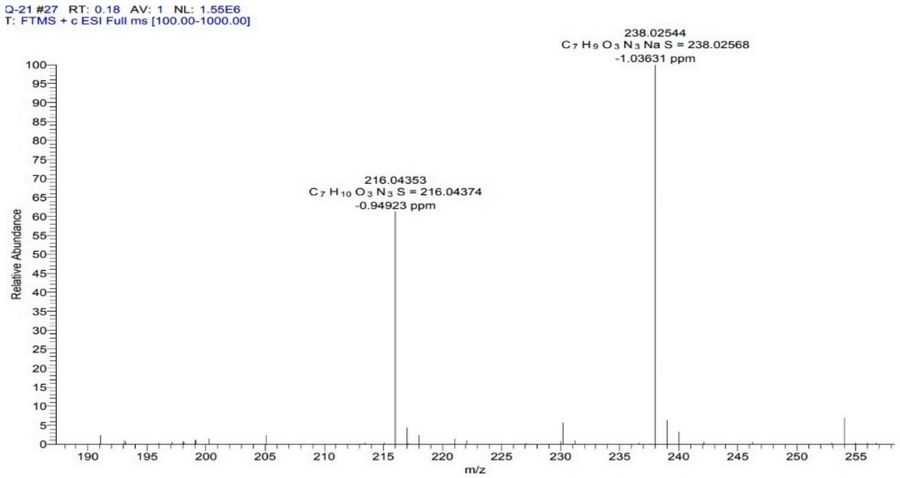

Compound 4c: ^{1}H and ^{13}C NMR and HRMS spectra.

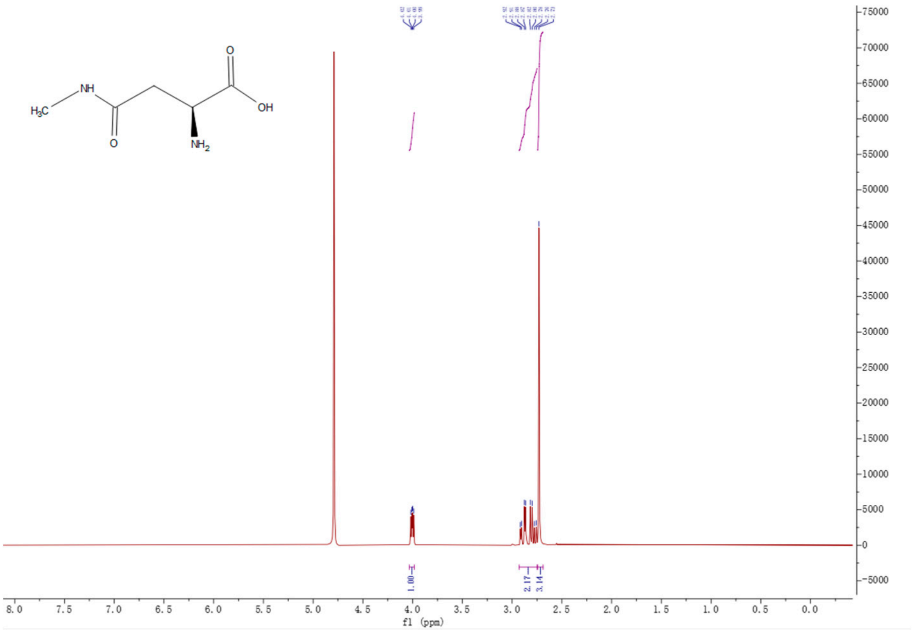

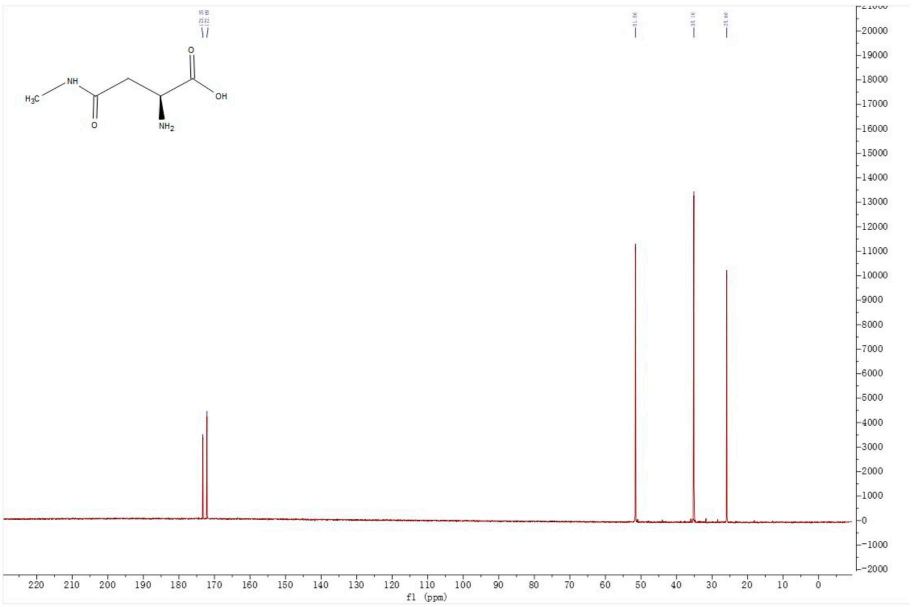

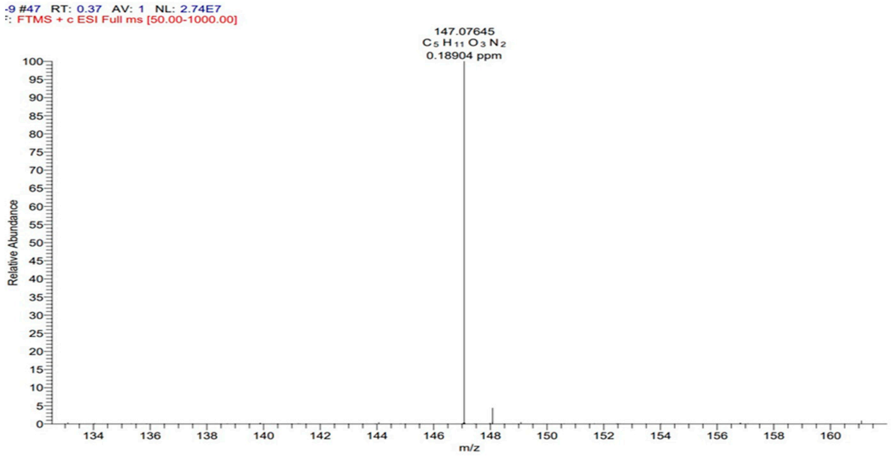

Compound 4d: ^{1}H and ^{13}C NMR and HRMS spectra.

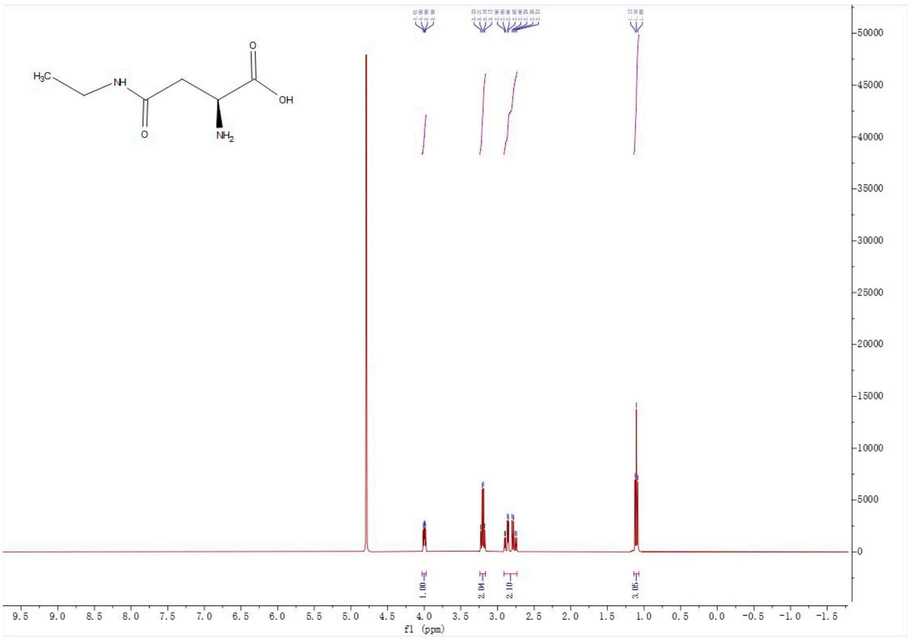

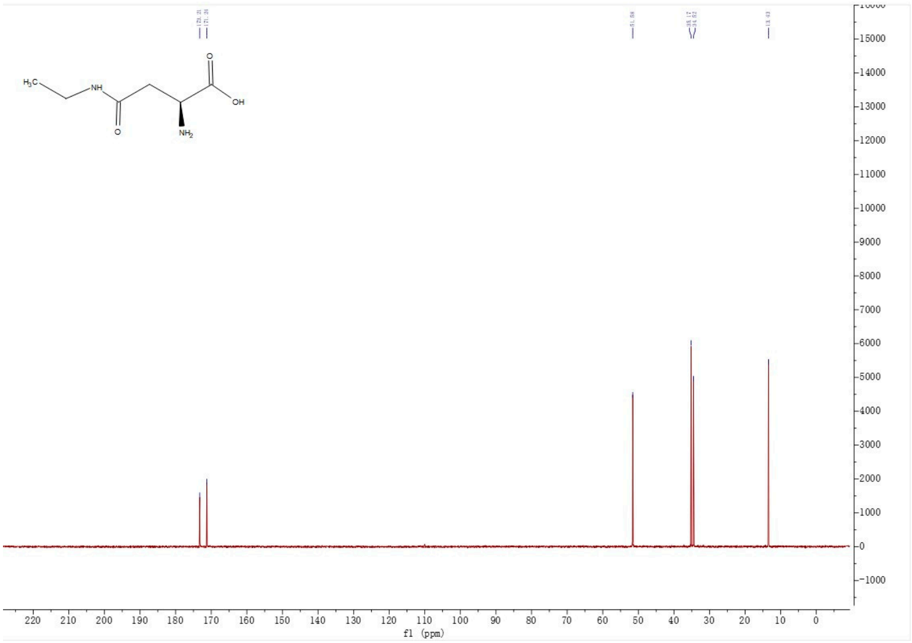

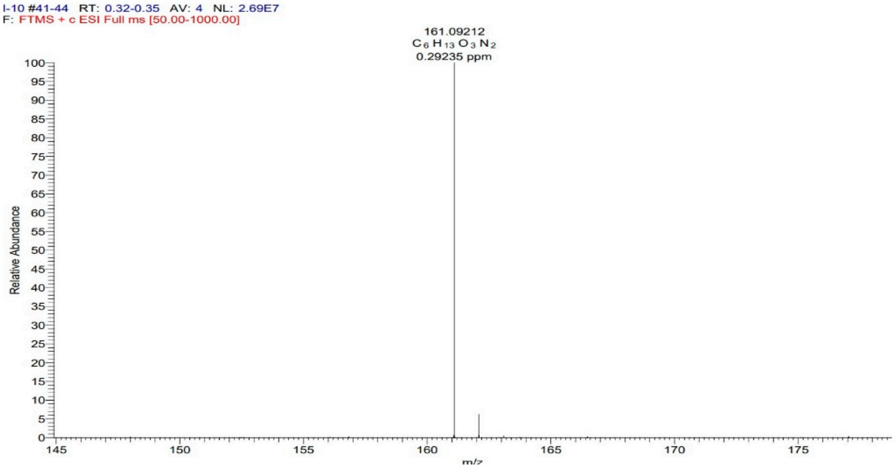

Compound 5: ^{1}H and ^{13}C NMR and HRMS spectra.

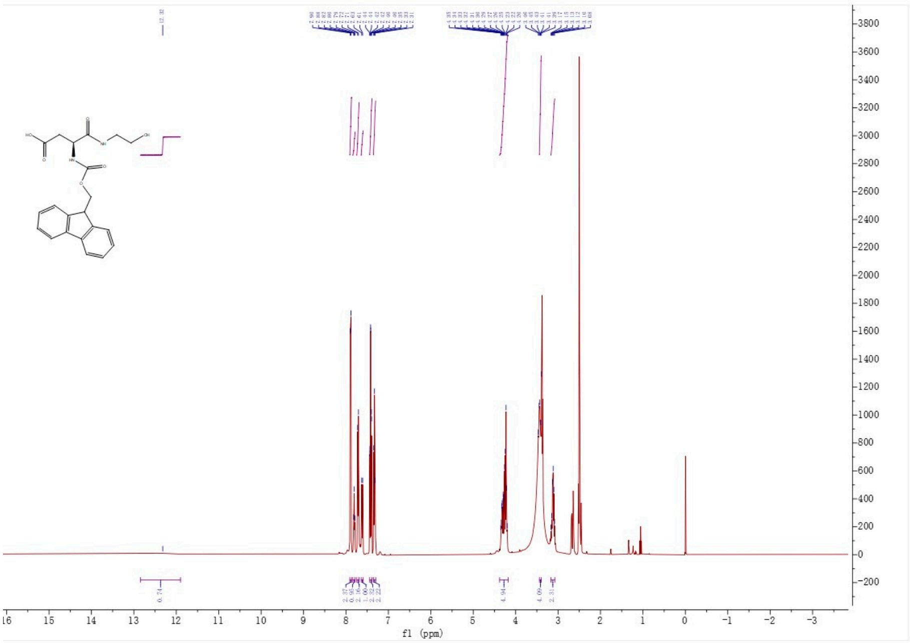

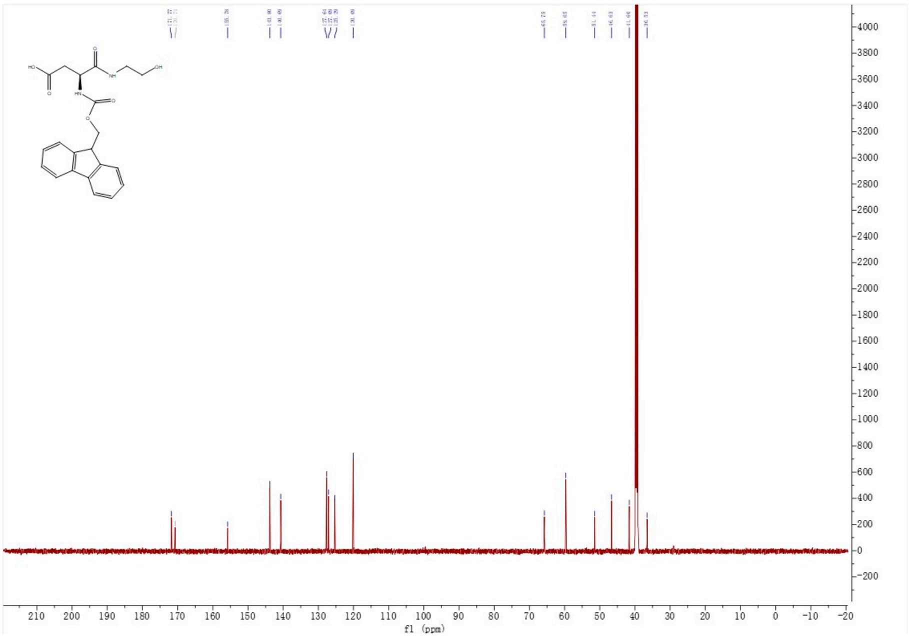

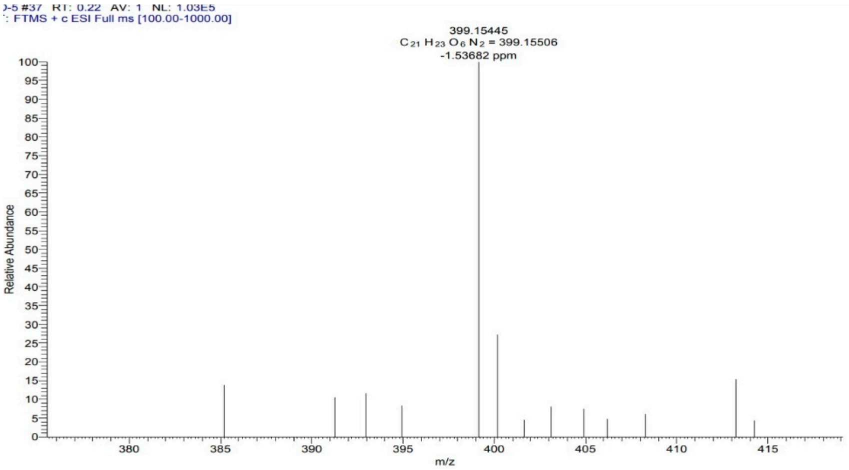

Compound 6: ¹H and ¹³C NMR and HRMS spectra.

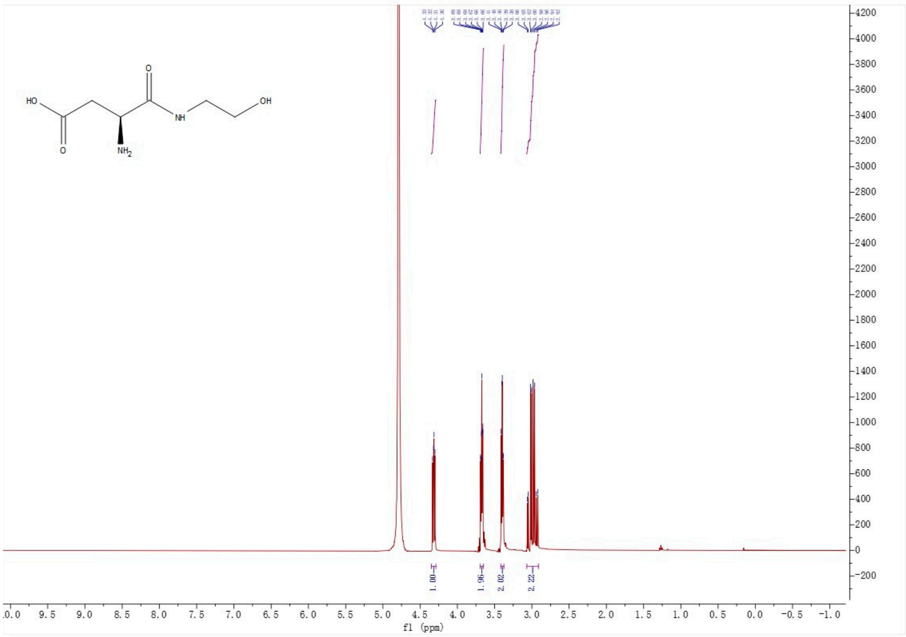

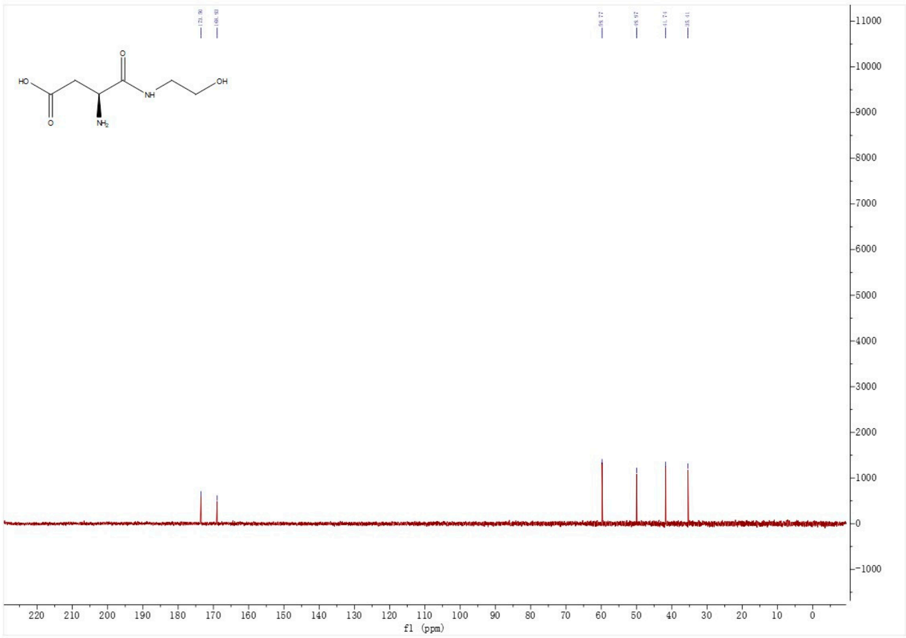

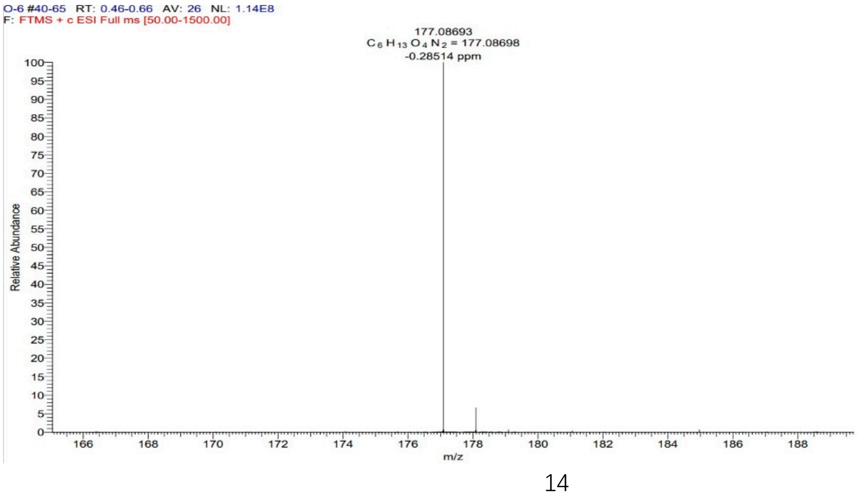
O-6 #40-65 RT: 0.46-0.66 AV: 26 NL: 1.14E8
F: FTMS + c ESI Full ms [50.00-1500.00]
177.08693
C6 H13 O4 N2 = 177.08698
-0.28514 ppm
Relative Abundance
m/z

Compound 8a: ^{1}H and ^{13}C NMR and HRMS spectra.

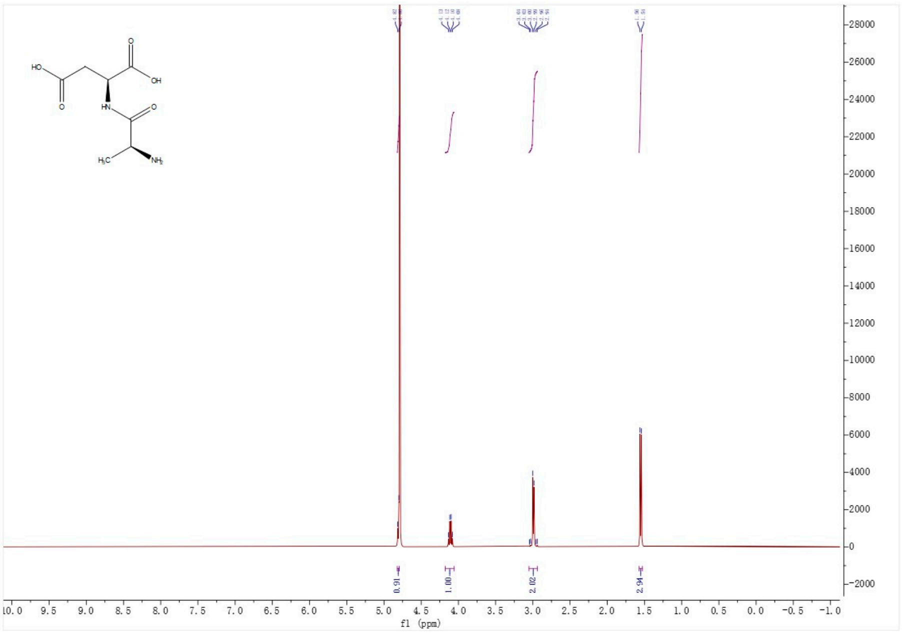

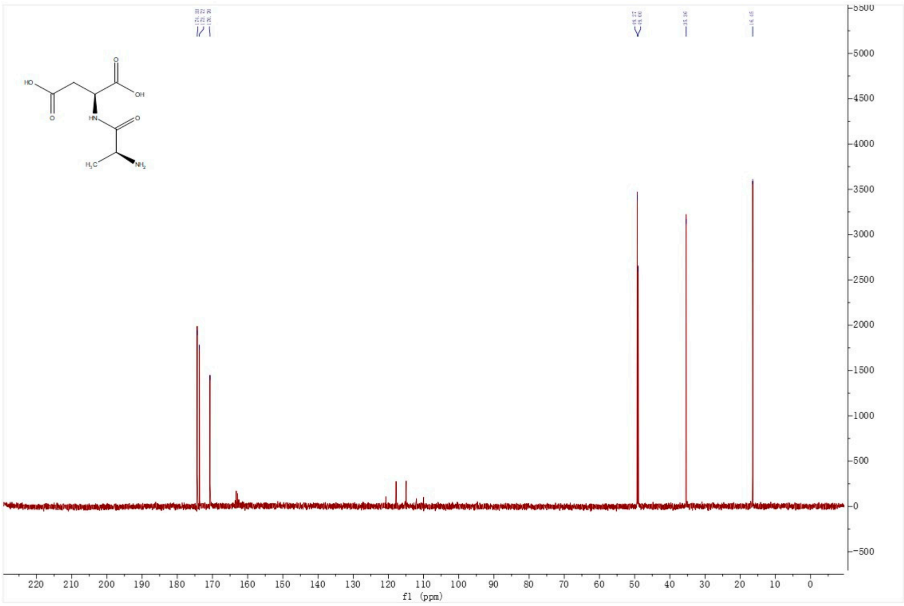

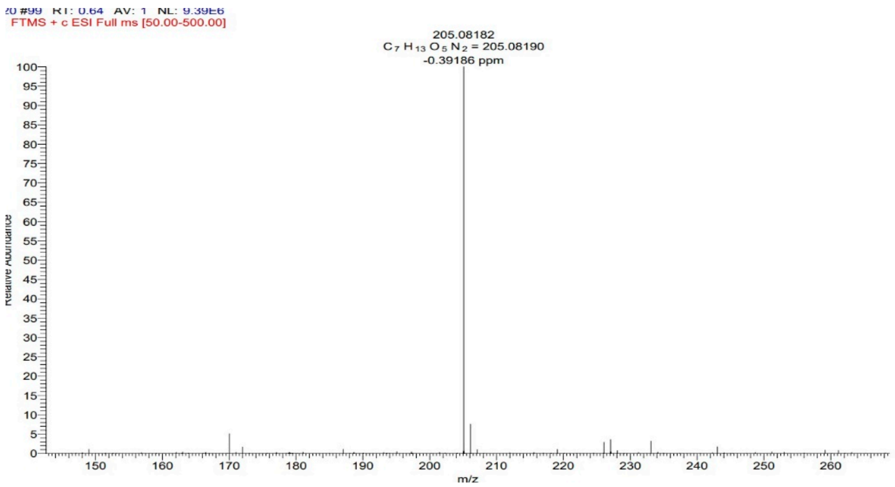

Compound 8b: ^{1}H and ^{13}C NMR and HRMS spectra.

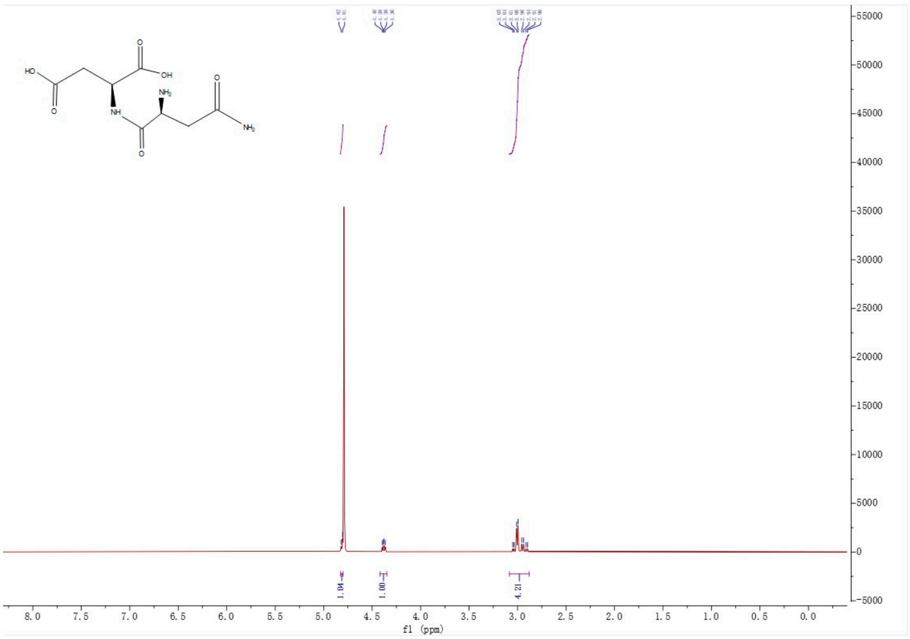

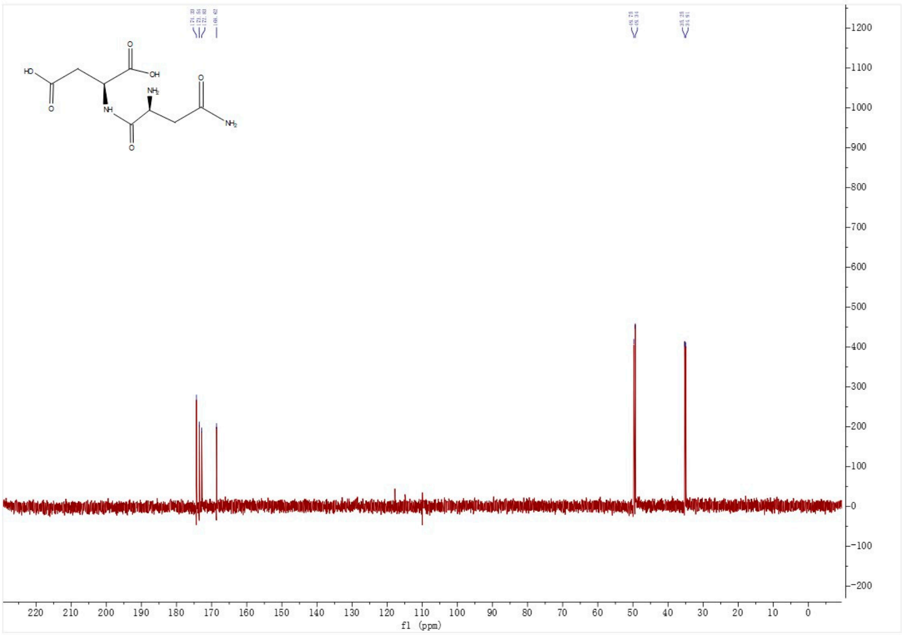

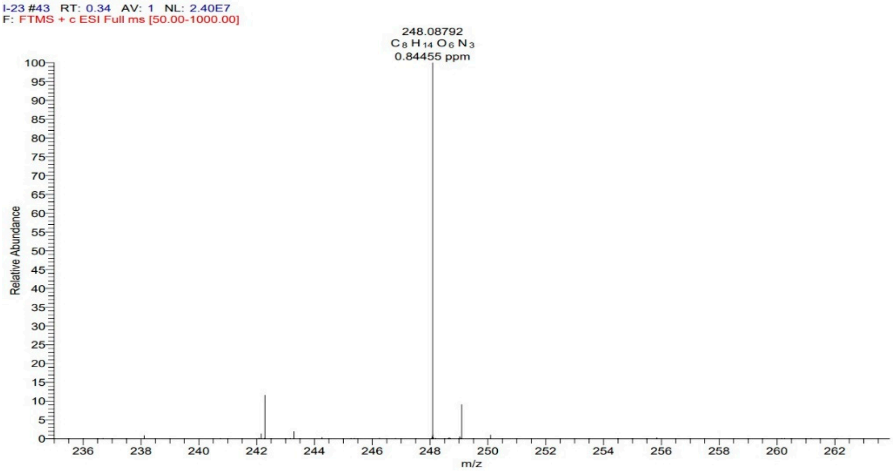

Compound 9: ^{1}H and ^{13}C NMR and HRMS spectra.

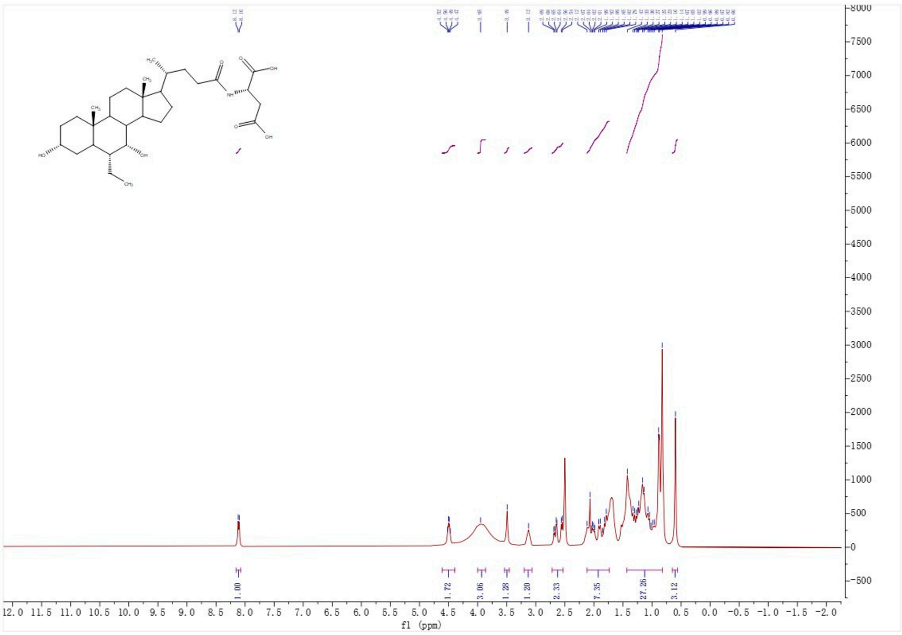
f1 (ppm)

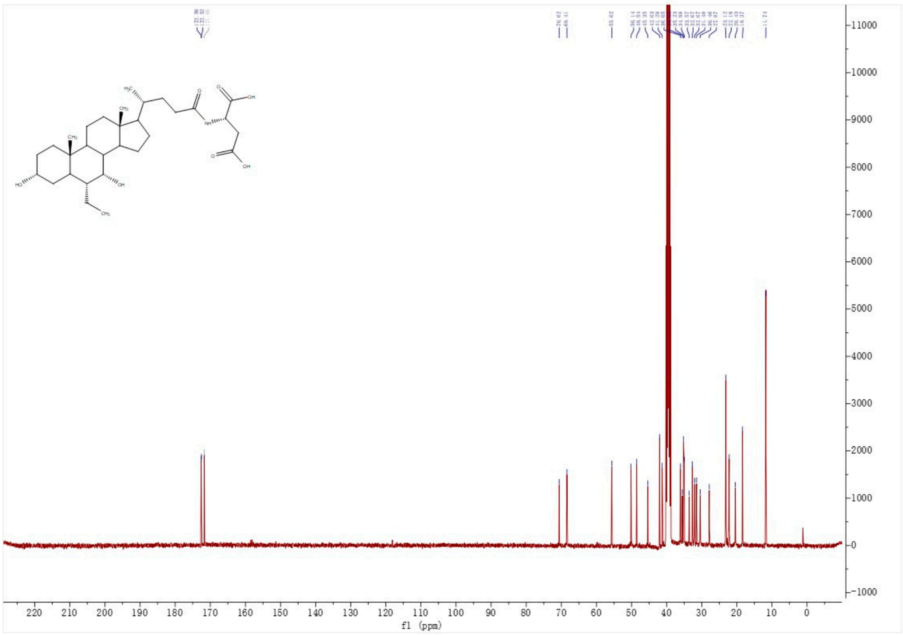
f1 (ppm)

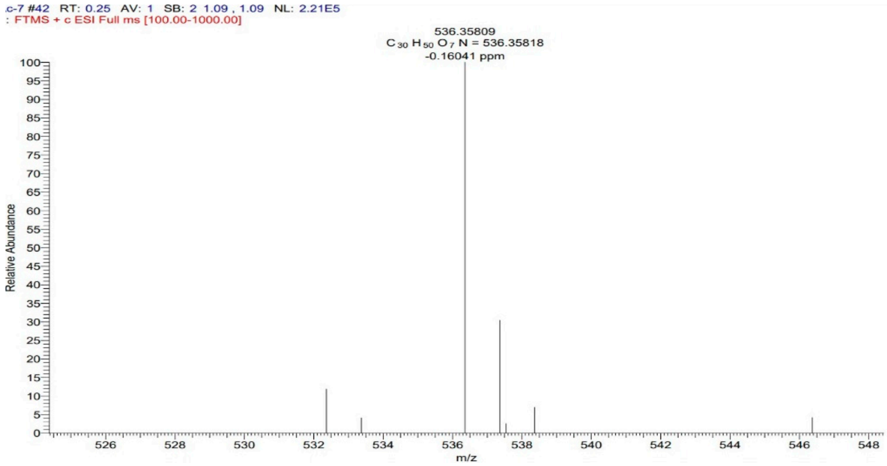
c-7 #42 RT: 0.25 AV: 1 SB: 2 1.09 , 1.09 NL: 2.21E5
: FTMS + c ESI Full ms [100.00-1000.00]
536.35809
C 30 H 50 O 7 N = 536.35818
-0.16041 ppm
Relative Abundance
m/z

Compound 11a: ^{1}H and ^{13}C NMR and HRMS spectra.

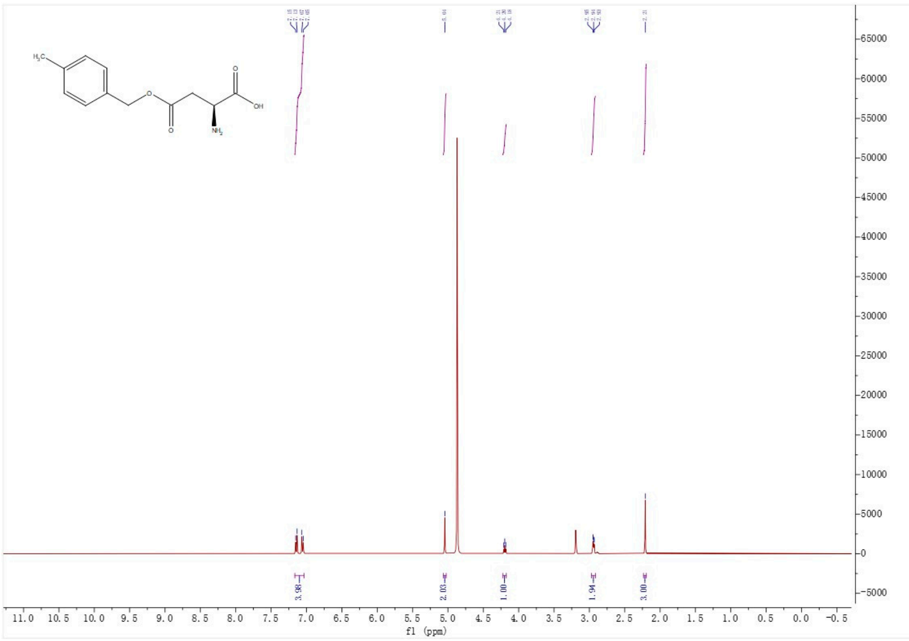

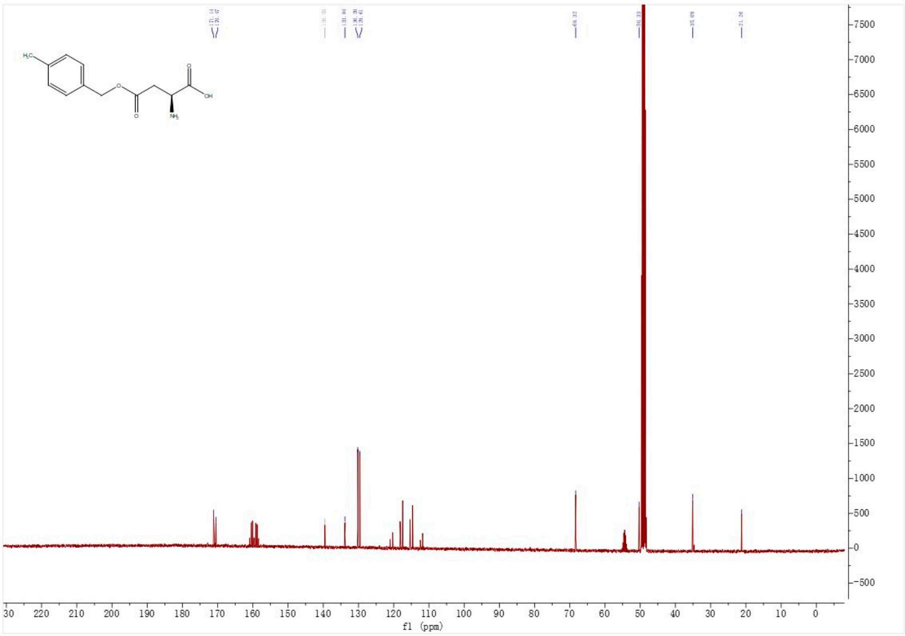

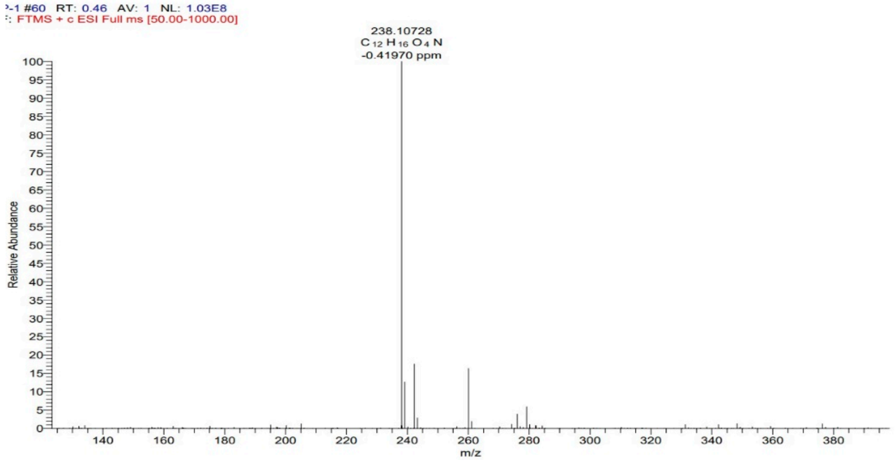

Compound 11b: ^{1}H and ^{13}C NMR and HRMS spectra.

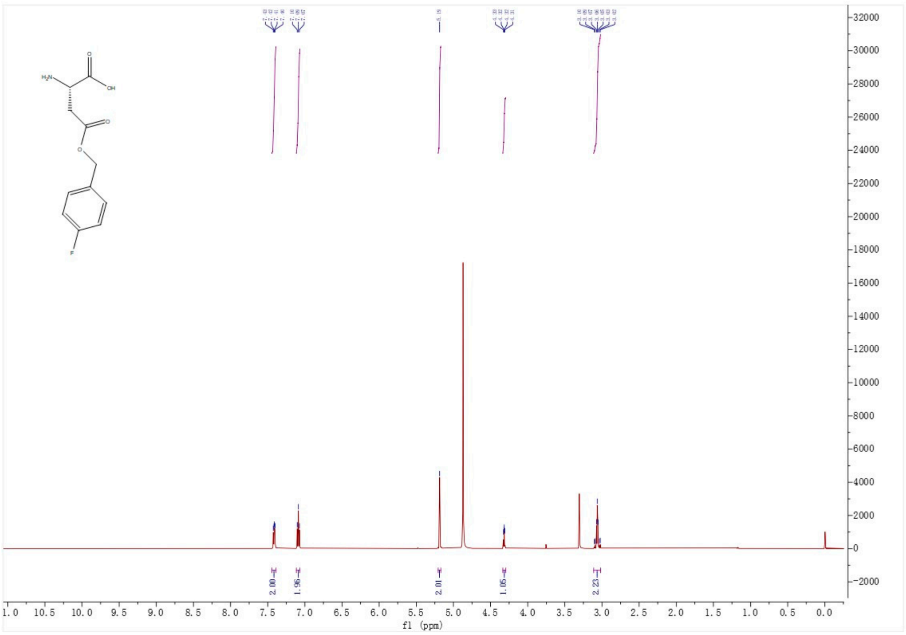

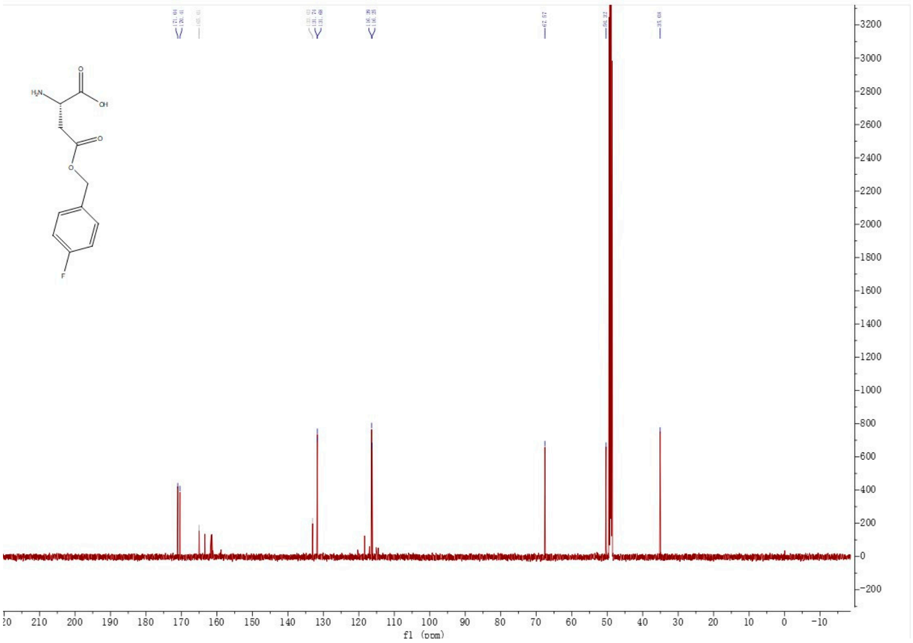

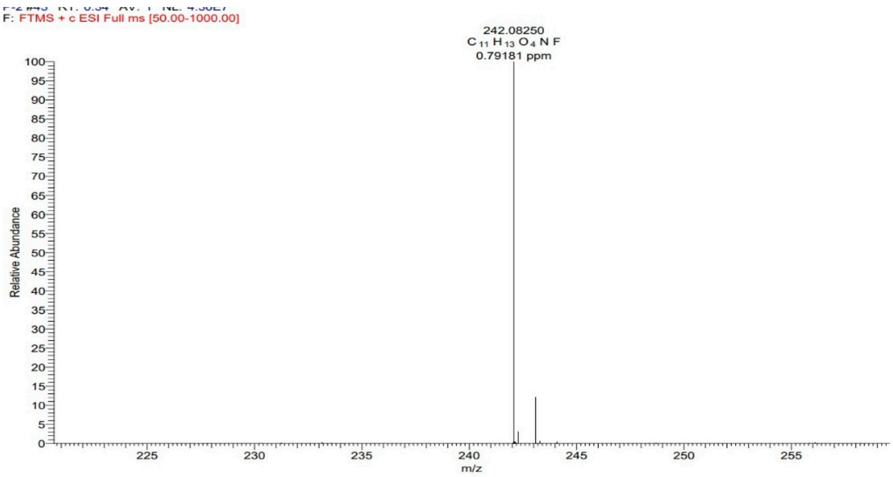

Compound 11c: ^{1}H and ^{13}C NMR and HRMS spectra.

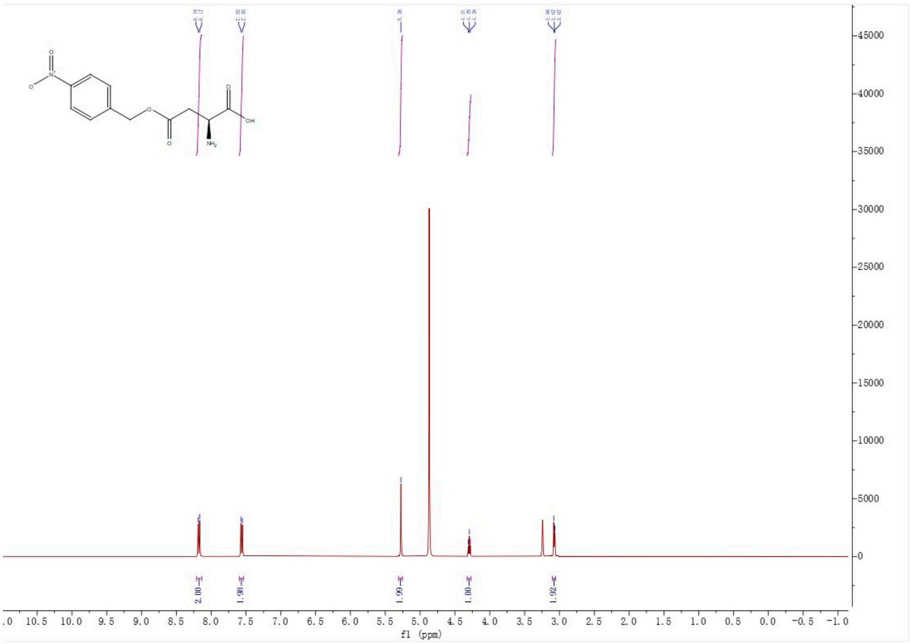
f1 (ppm)

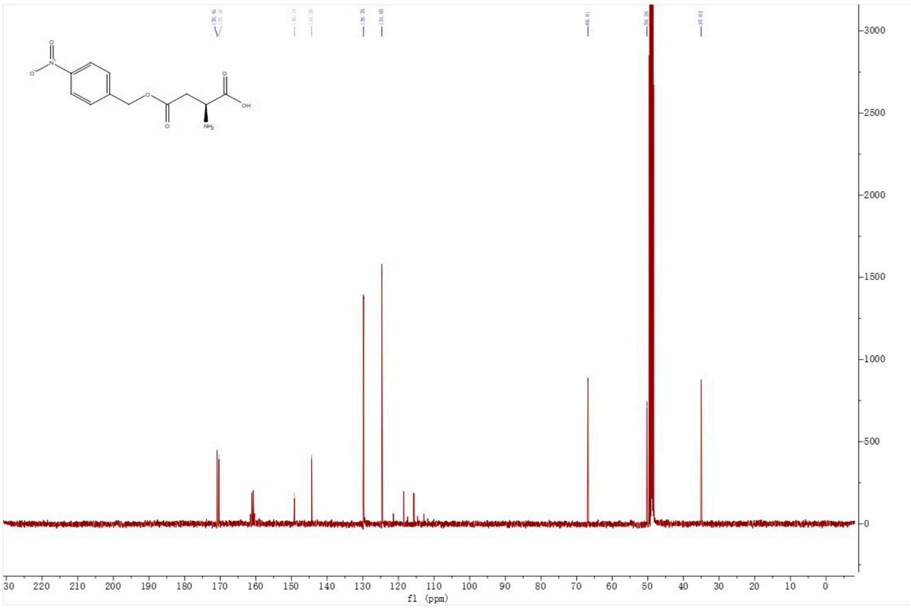
f1 (ppm)

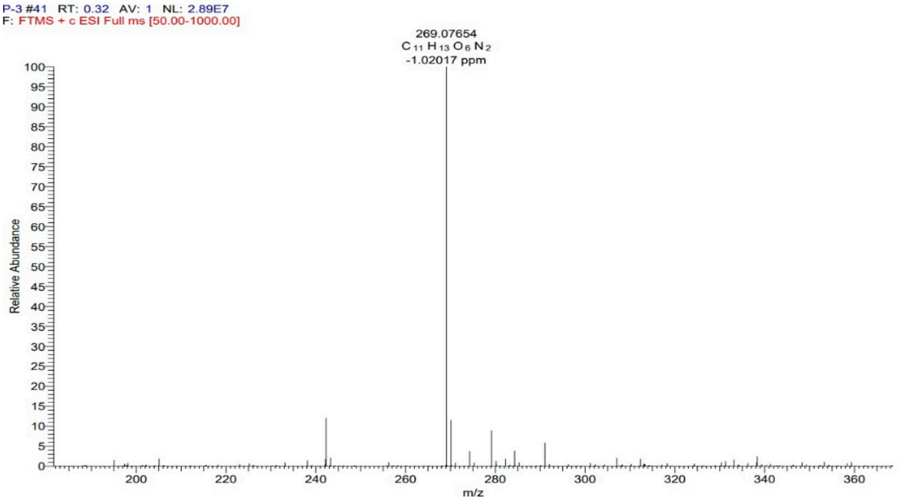
P-3 #41 RT: 0.32 AV: 1 NL: 2.89E7
F: FTMS + c ESI Full ms [50.00-1000.00]
269.07654
C 11 H 13 O 6 N 2
-1.02017 ppm
Relative Abundance
m/z

Compound 11d: ^{1}H and ^{13}C NMR and HRMS spectra.

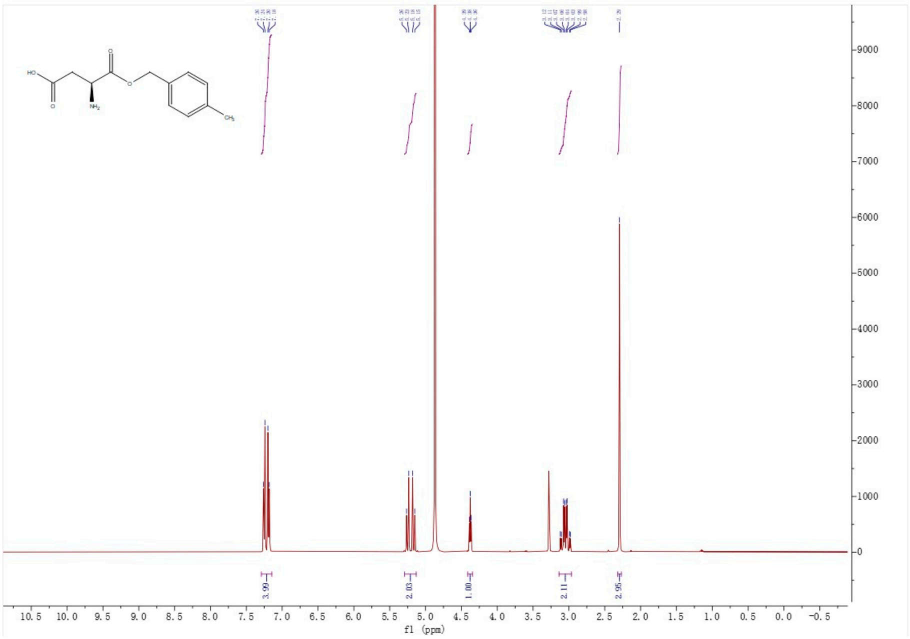

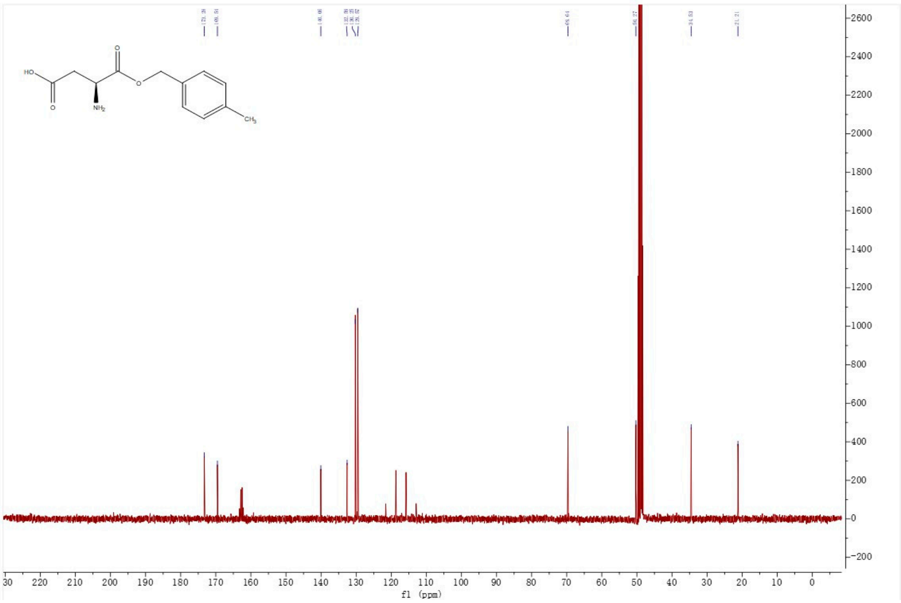

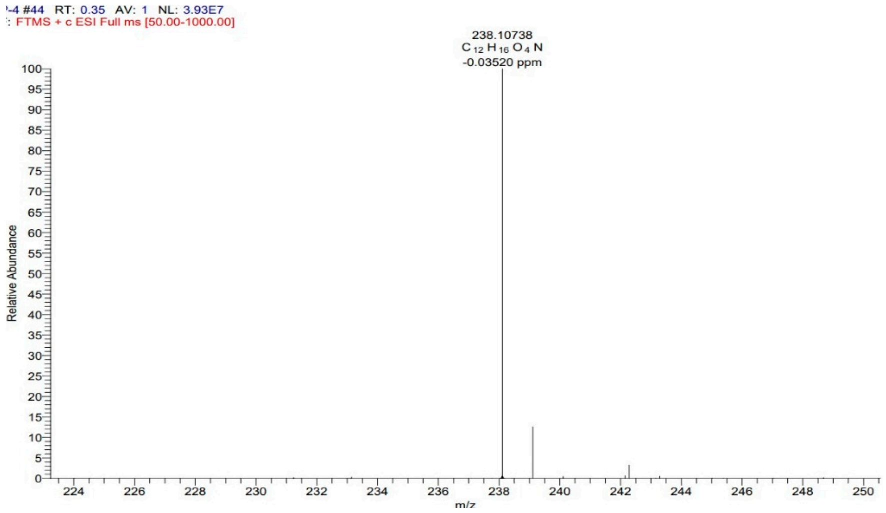

Compound 11e: ^{1}H and ^{13}C NMR and HRMS spectra.

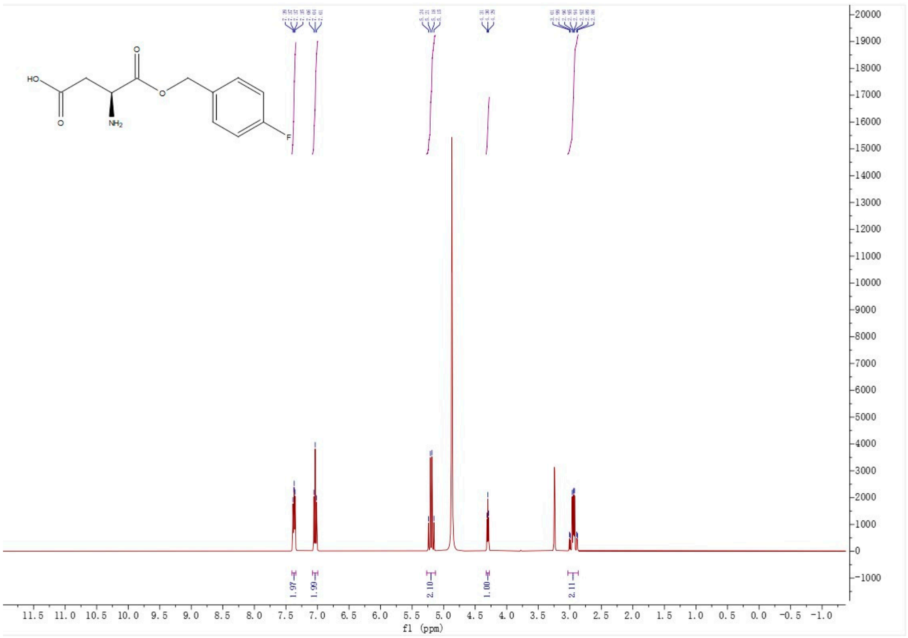

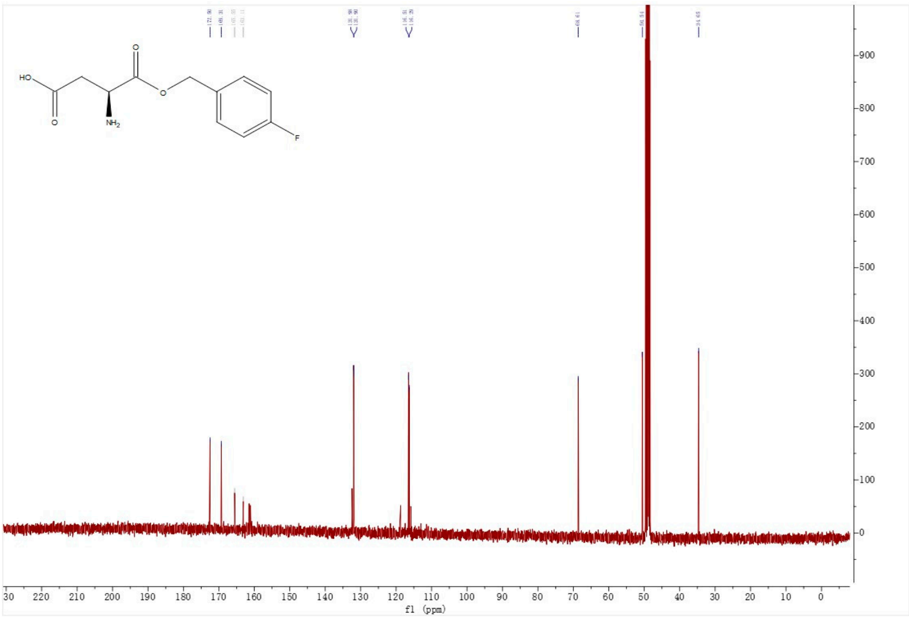

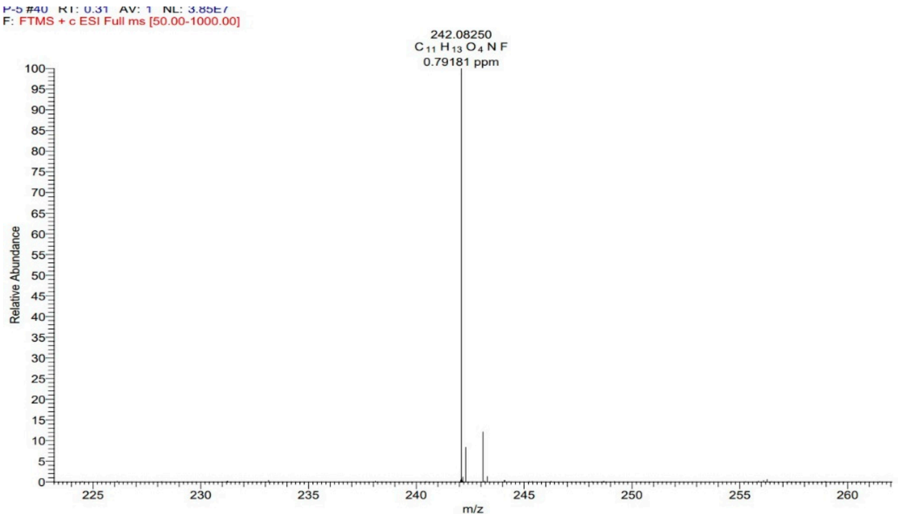

Compound 11f: ^{1}H and ^{13}C NMR and HRMS spectra.

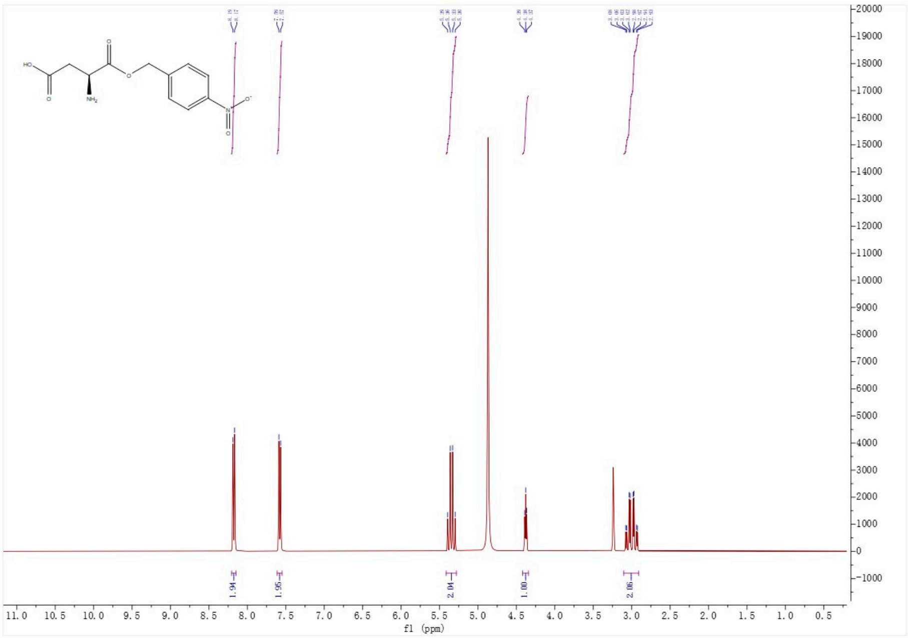
1.94
1.95
2.04
1.00
2.06
f1 (ppm)

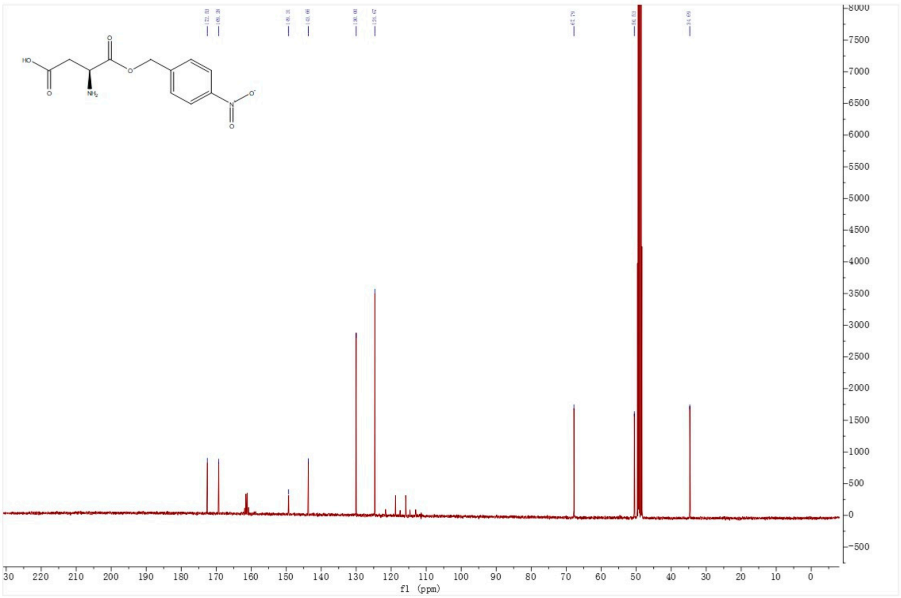
f1 (ppm)

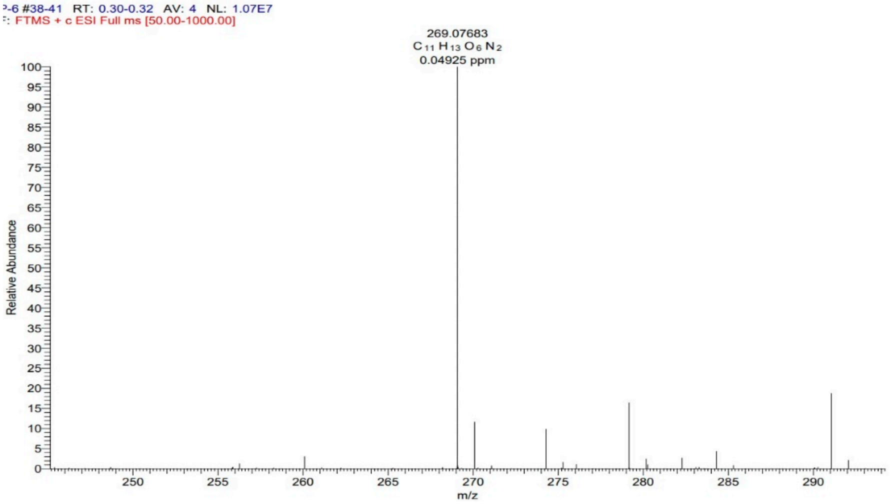
-6 #38-41 RT: 0.30-0.32 AV: 4 NL: 1.07E7
: FTMS + c ESI Full ms [50.00-1000.00]
269.07683
C 11 H 13 O 6 N 2
0.04925 ppm
Relative Abundance
m/z

Compound 20a: ^{1}H and ^{13}C NMR and HRMS spectra.

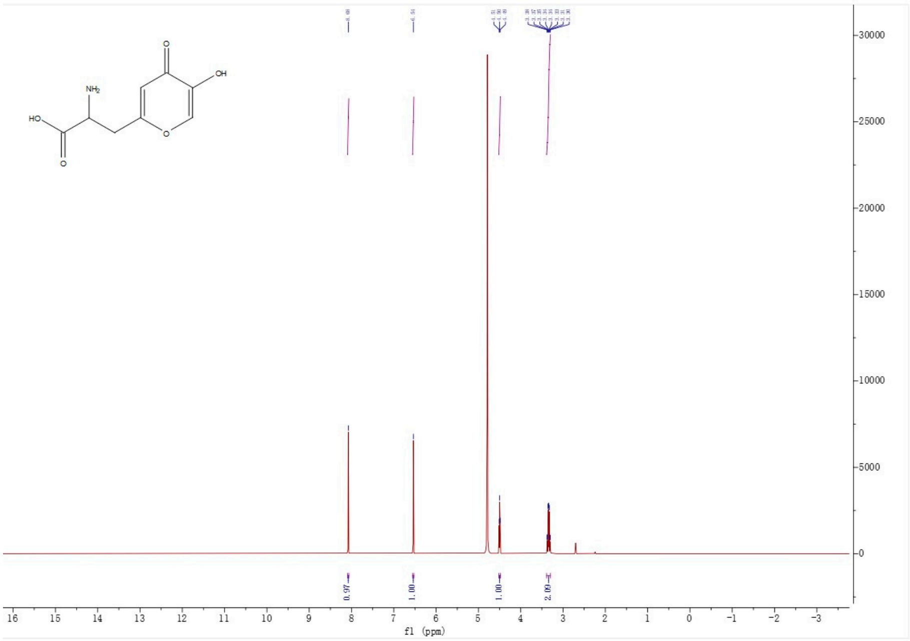

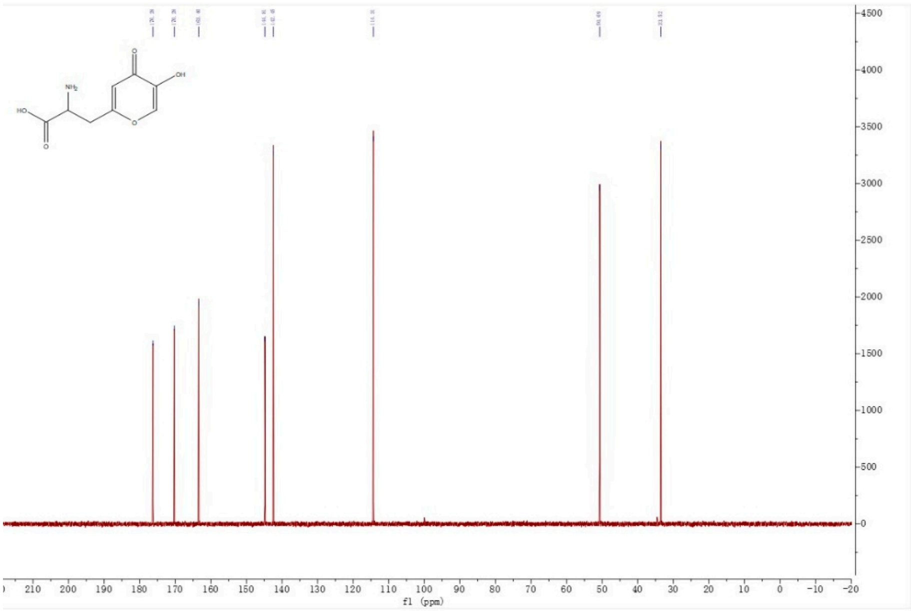

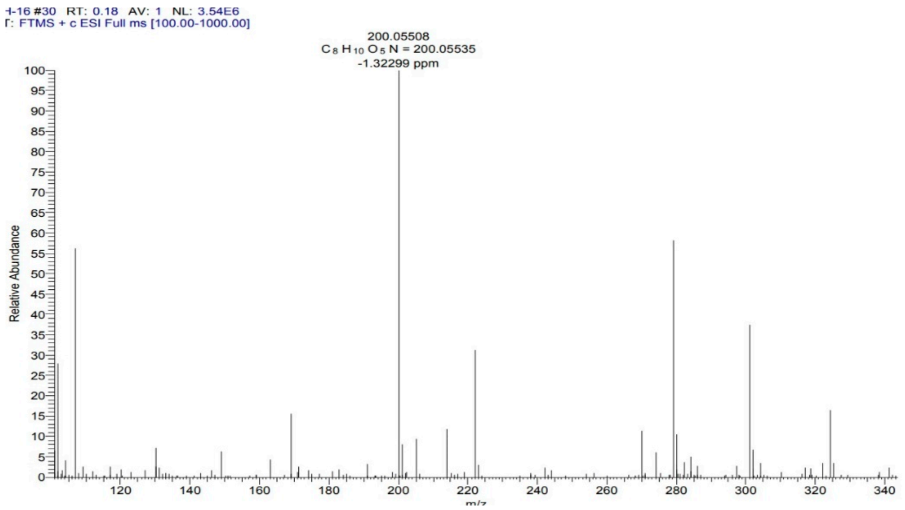

Compound 20b: ^{1}H and ^{13}C NMR and HRMS spectra.

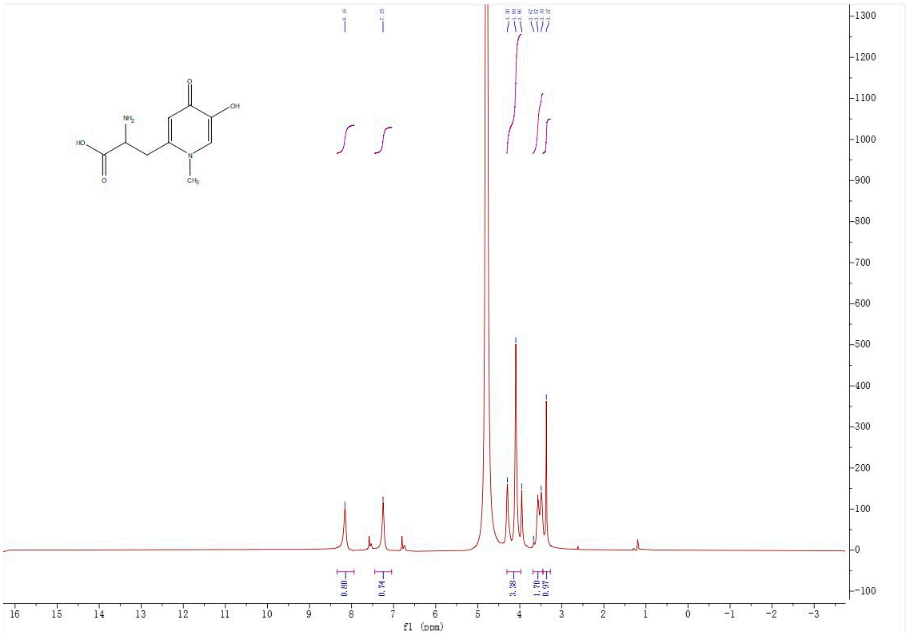

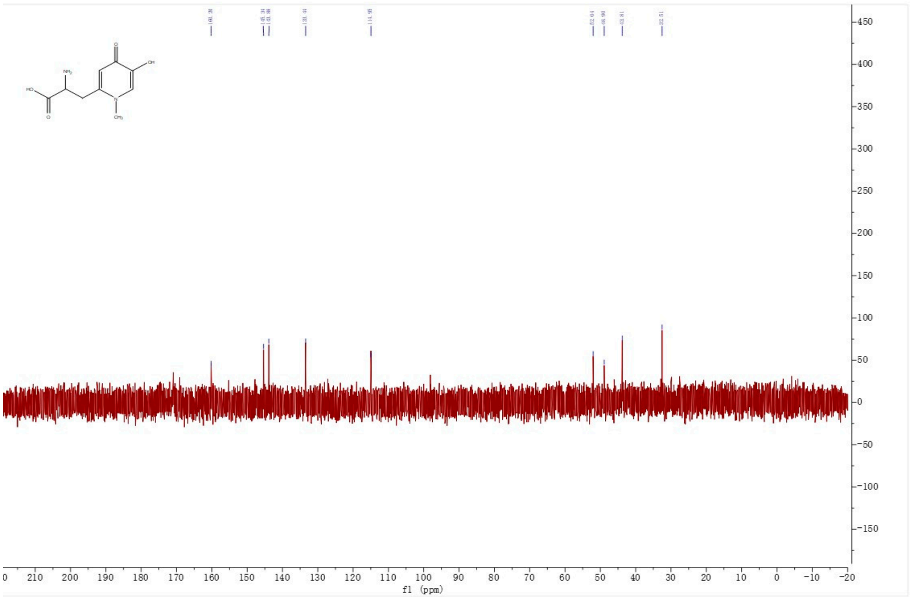

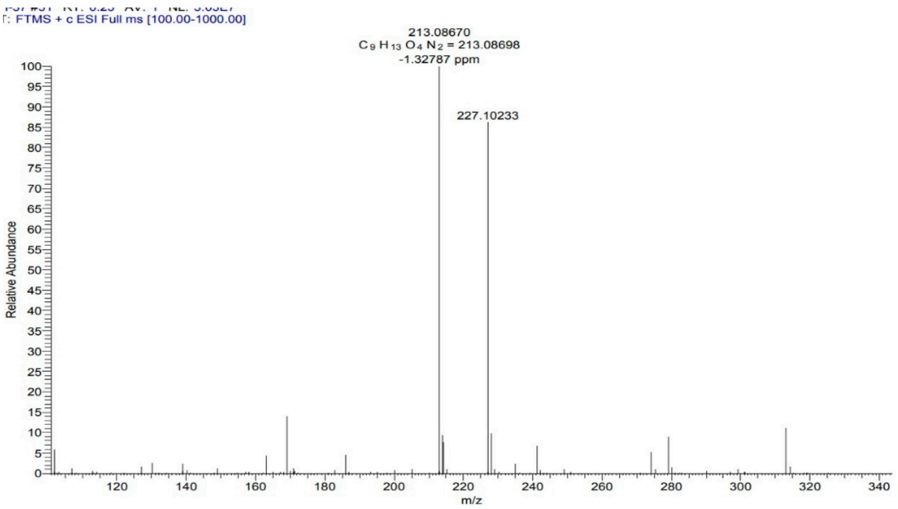

Compound 23a: ^{1}H and ^{13}C NMR and HRMS spectra.

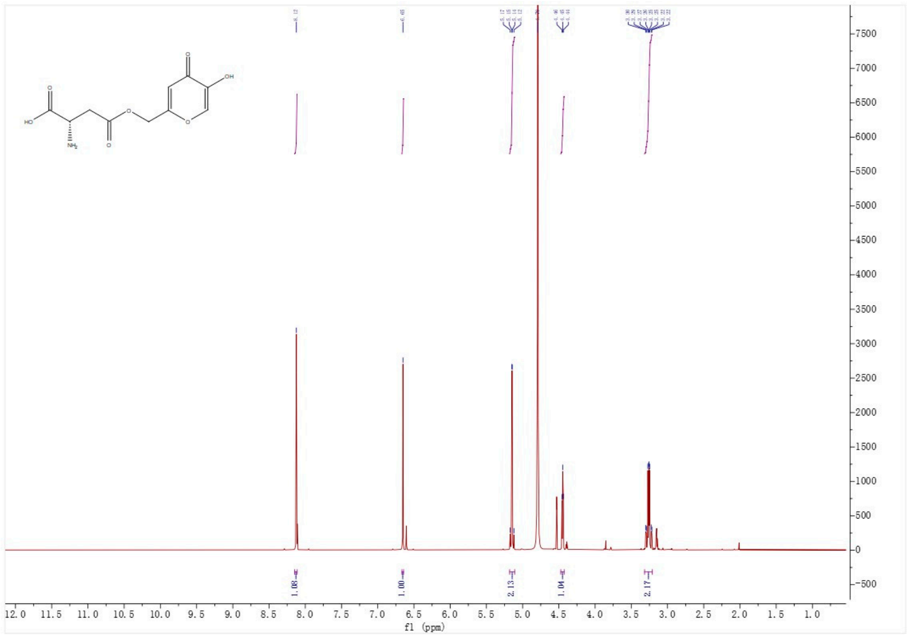

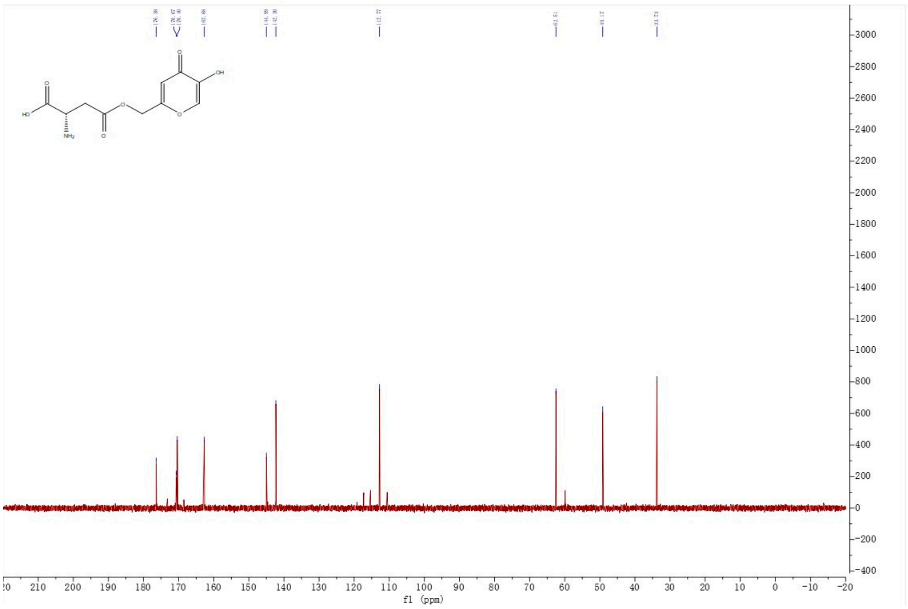

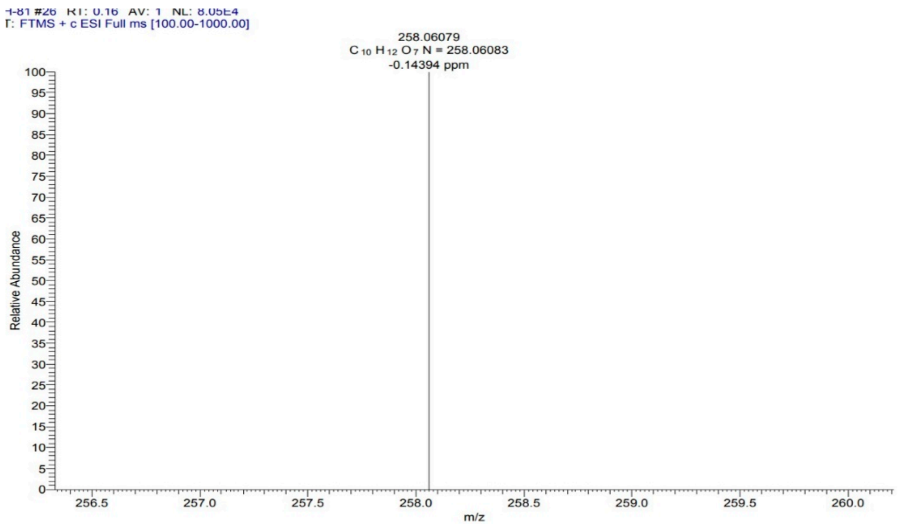

Compound 23b: ^{1}H and ^{13}C NMR and HRMS spectra.

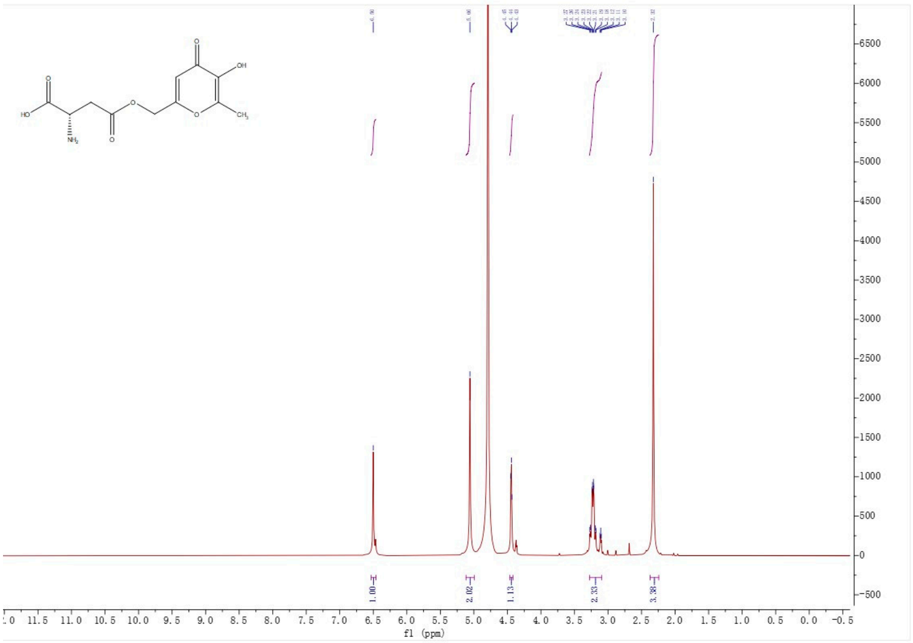

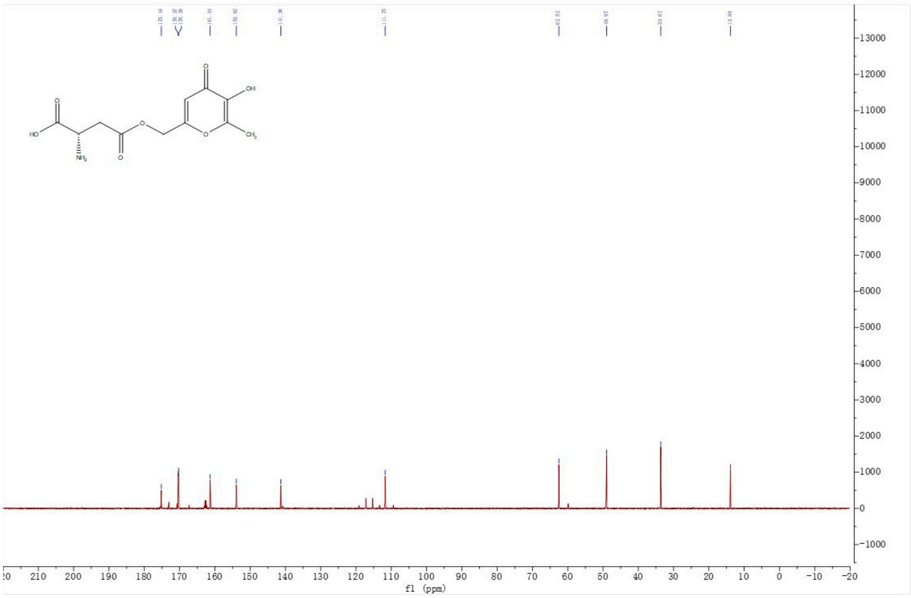

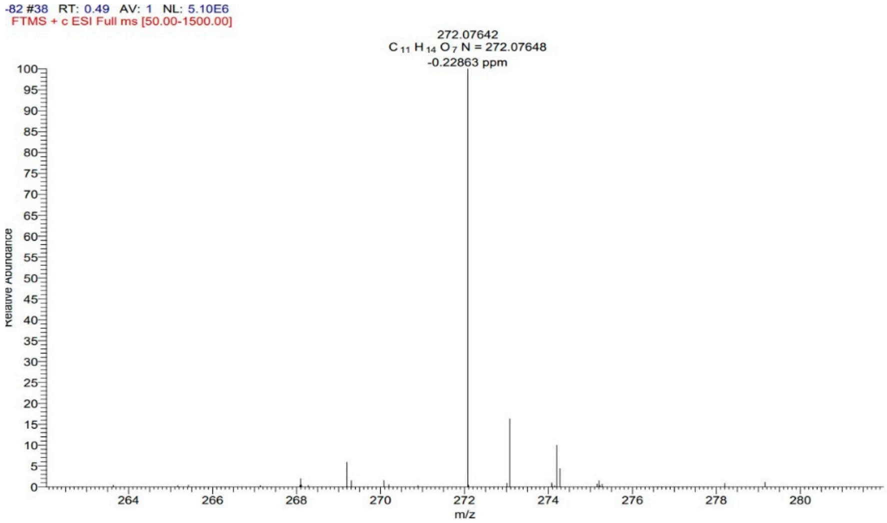

Compound 28a: ^{1}H and ^{13}C NMR and HRMS spectra.

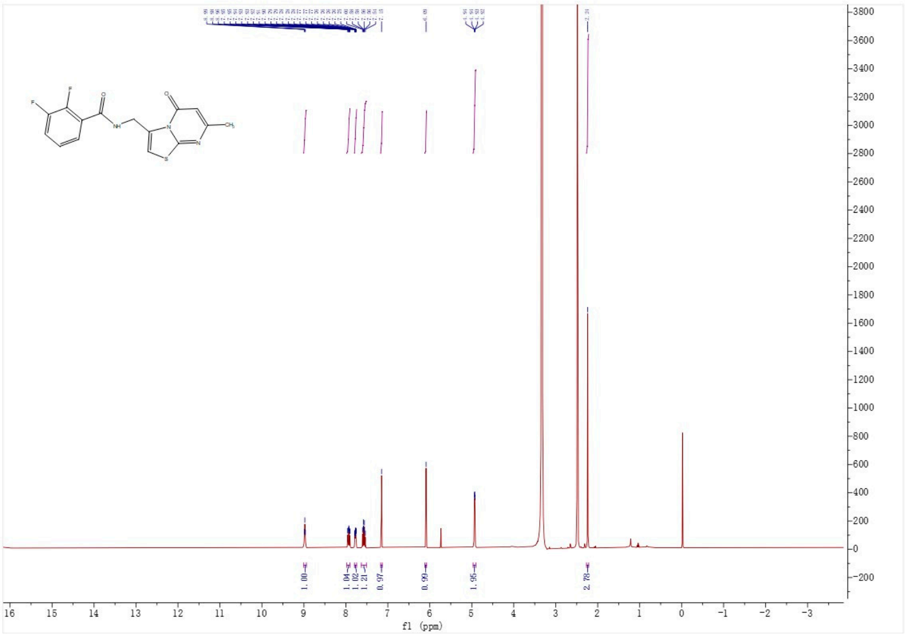
f1 (ppm)

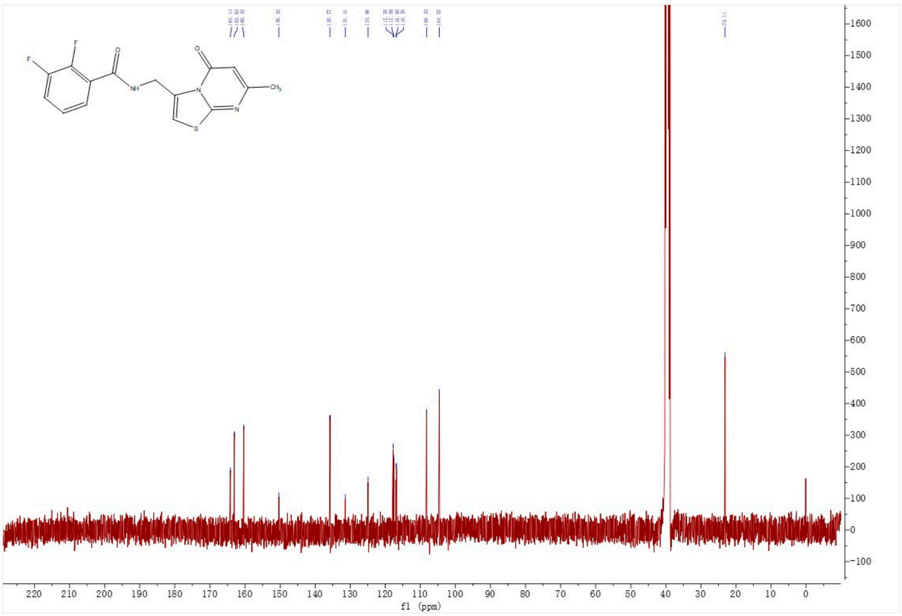
f1 (ppm)

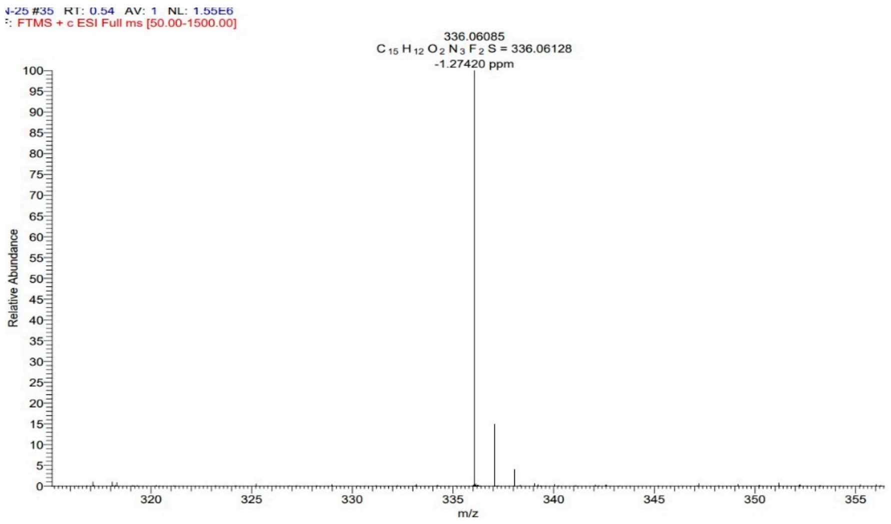

Compound 29: ^{1}H and ^{13}C NMR and HRMS spectra.

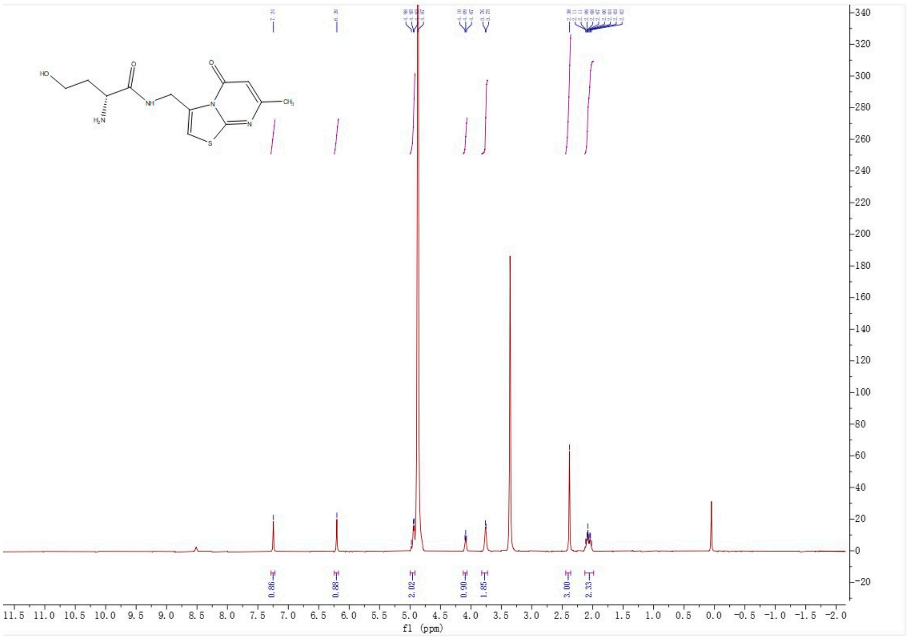

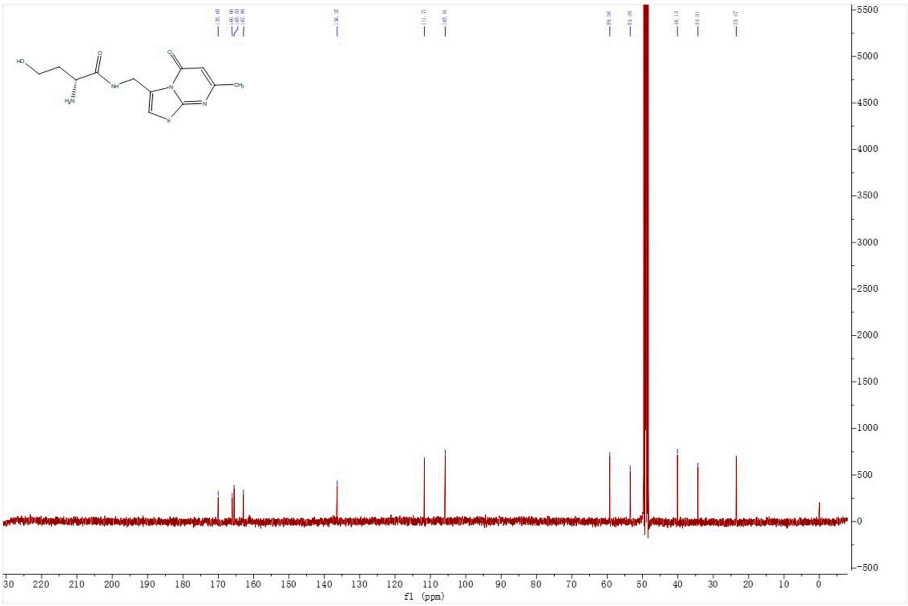

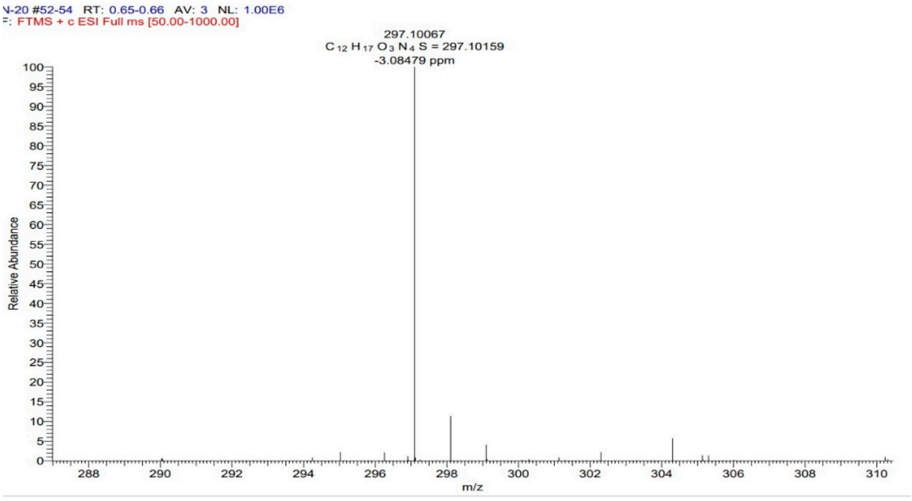

Compound 31: ^{1}H and ^{13}C NMR and HRMS spectra.

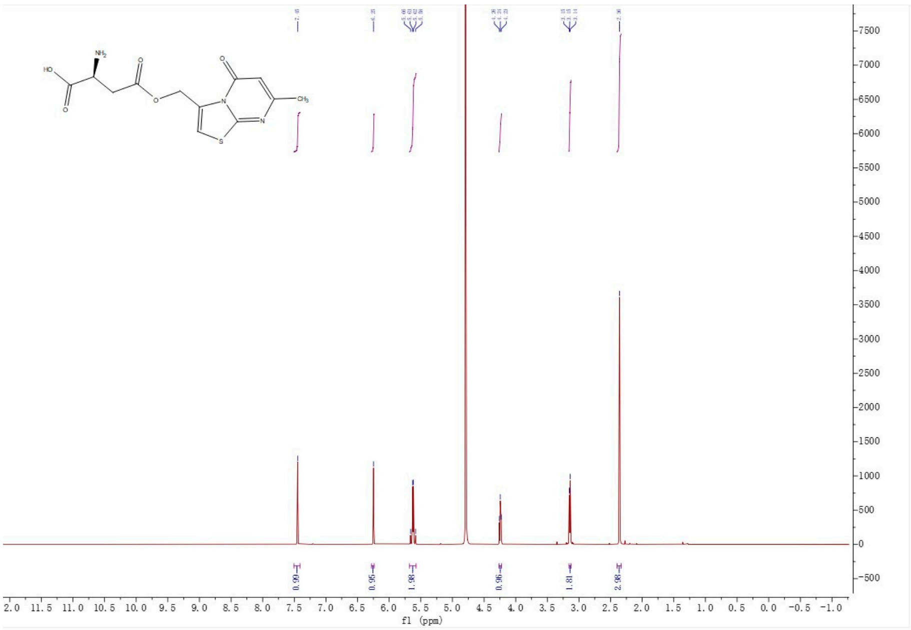

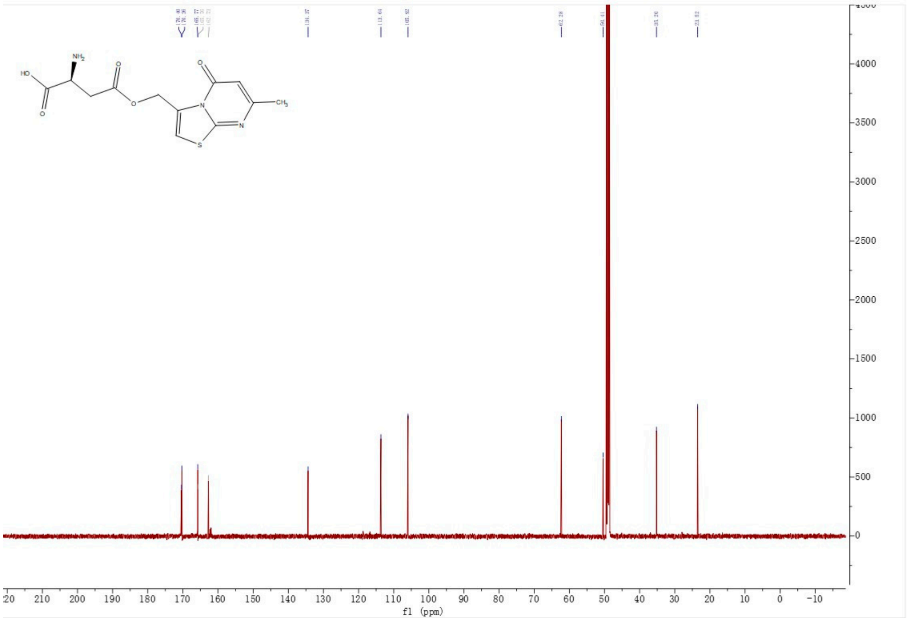

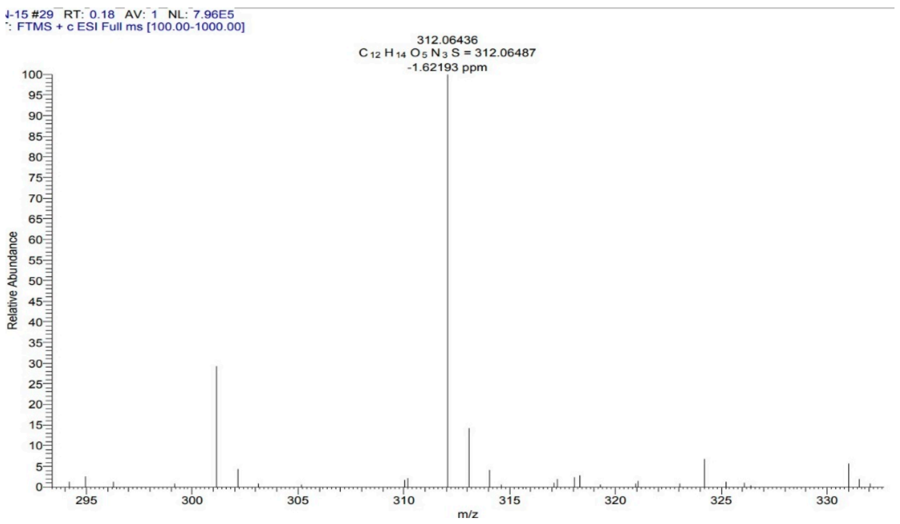

Compound 32a: ^{1}H and ^{13}C NMR and HRMS spectra.

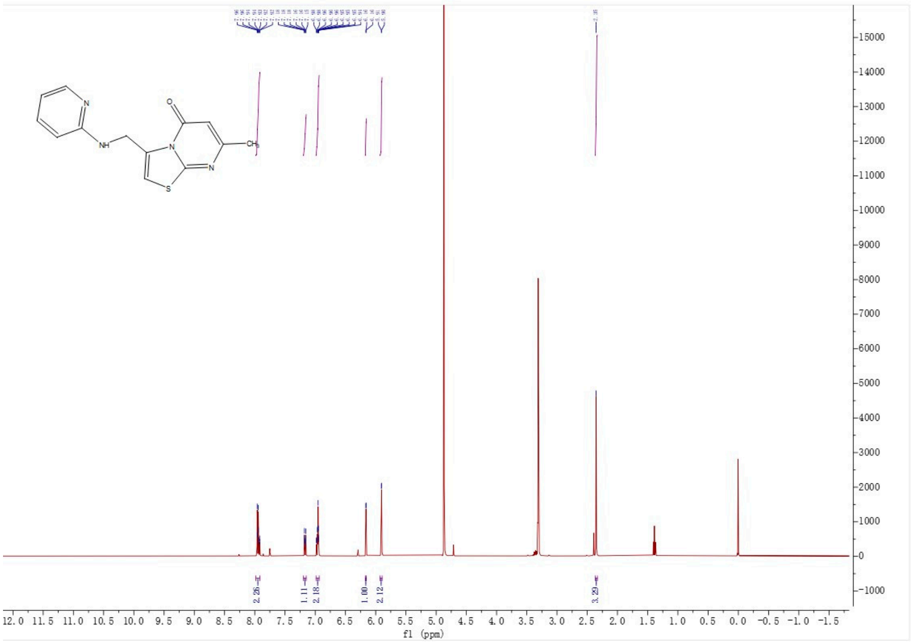

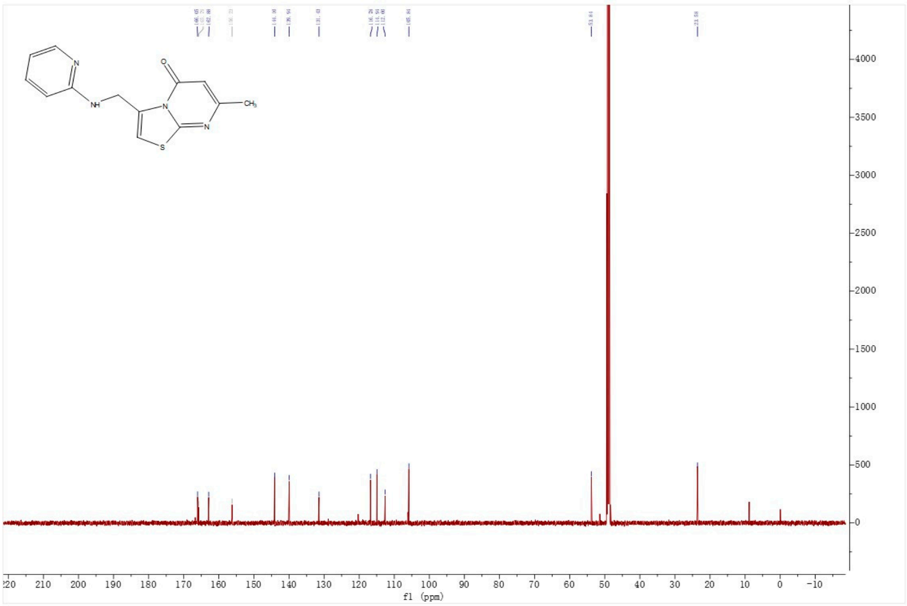

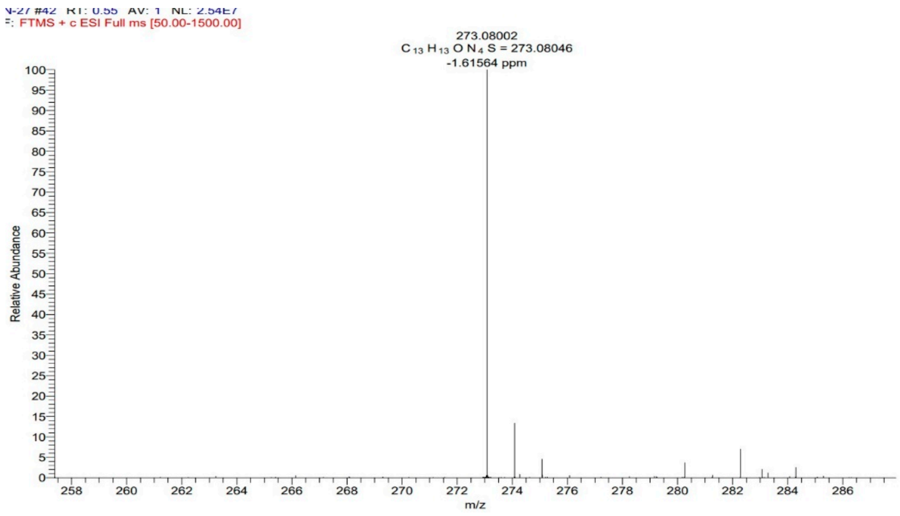

Compound 33: ^{1}H and ^{13}C NMR and HRMS spectra.

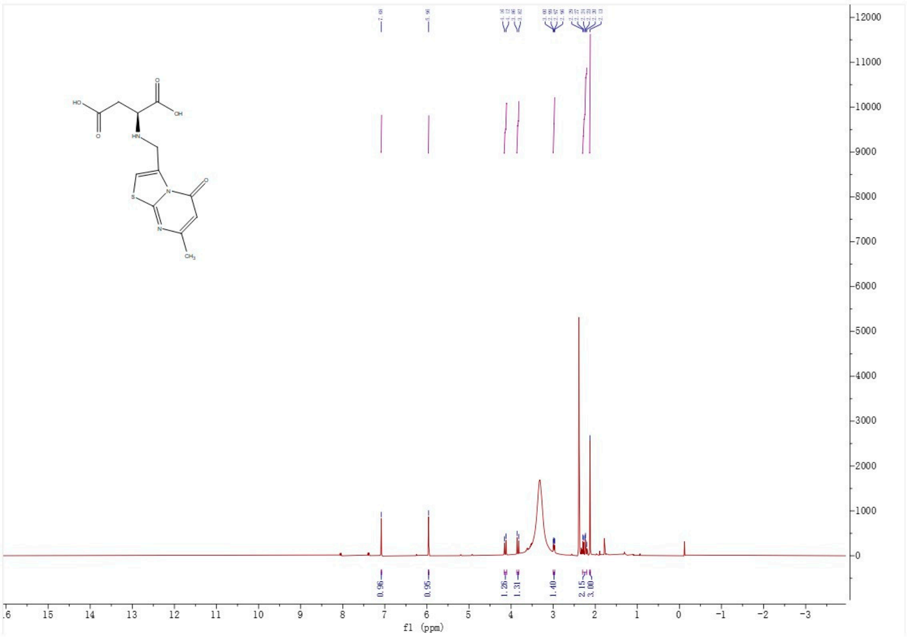

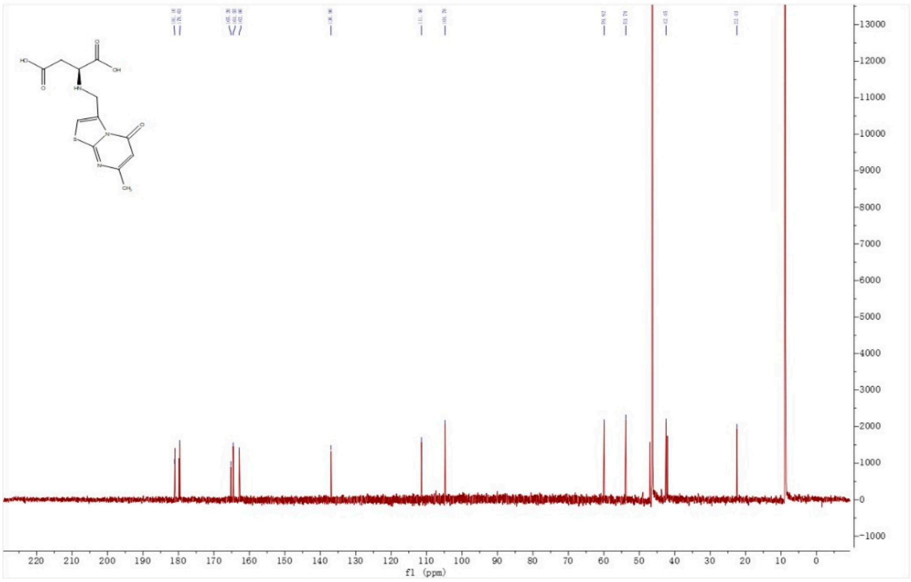

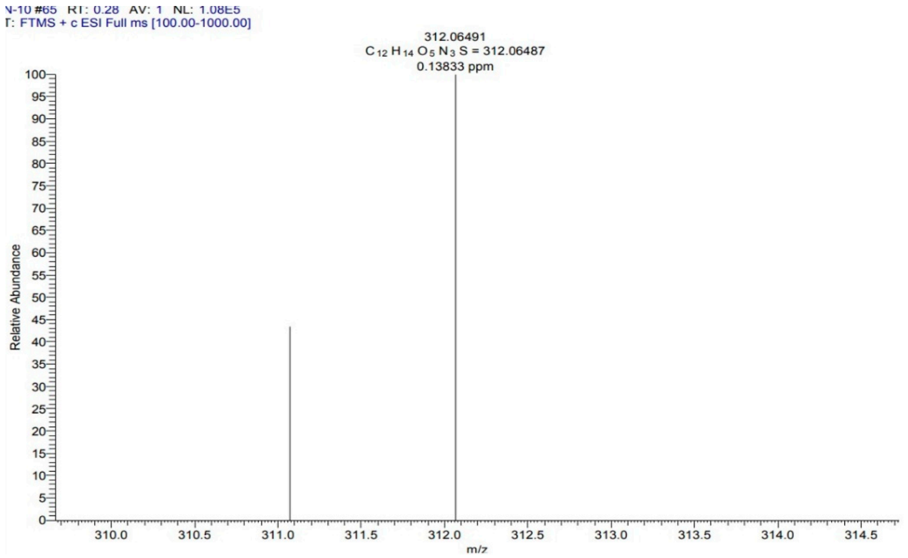

Compound 34a: ^{1}H and ^{13}C NMR and HRMS spectra.

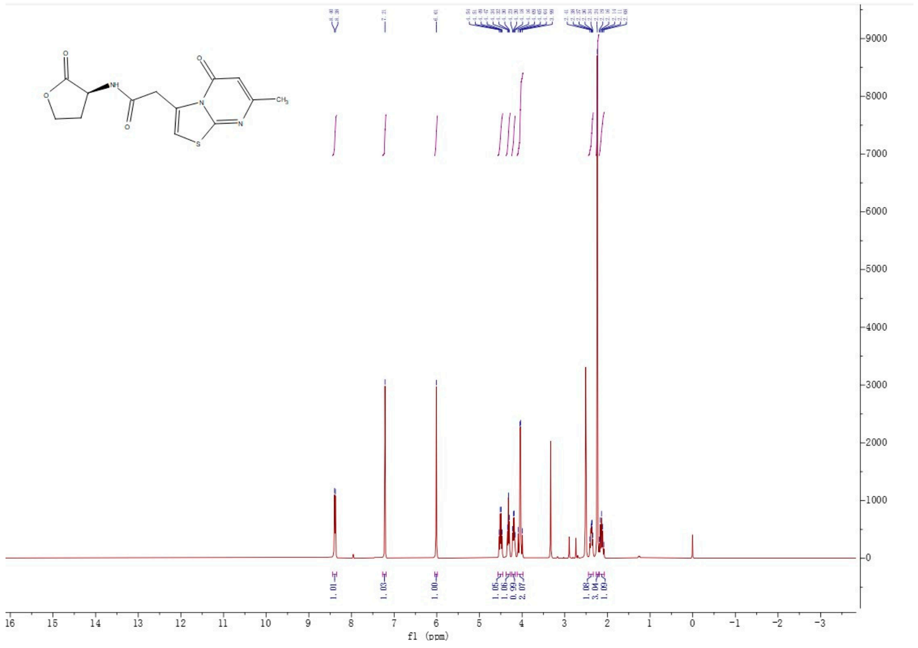

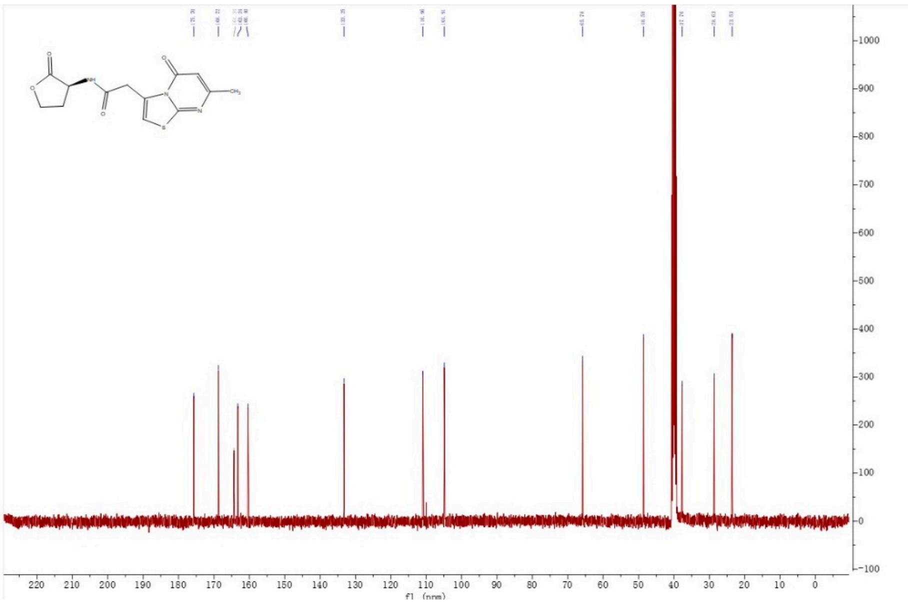

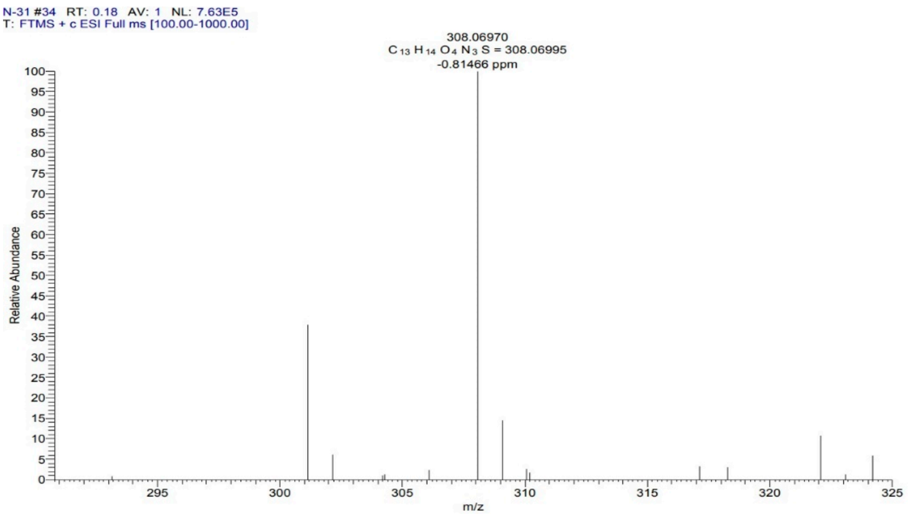

Compound 35: ^{1}H and ^{13}C NMR and HRMS spectra.

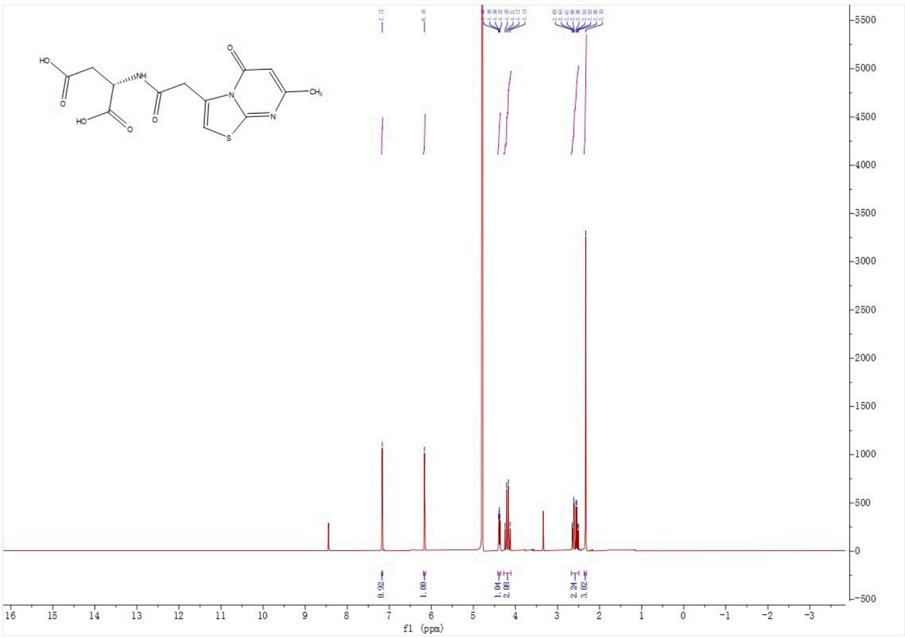

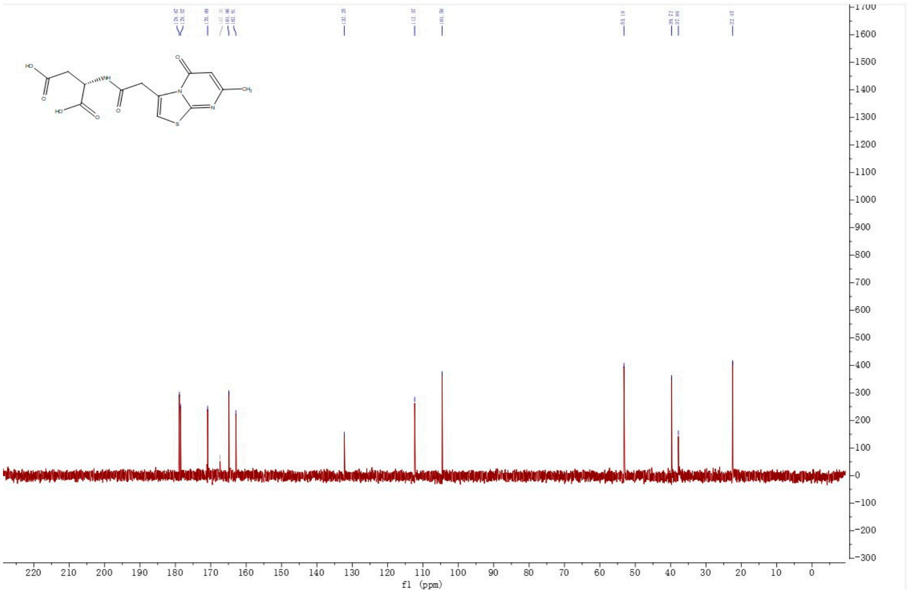

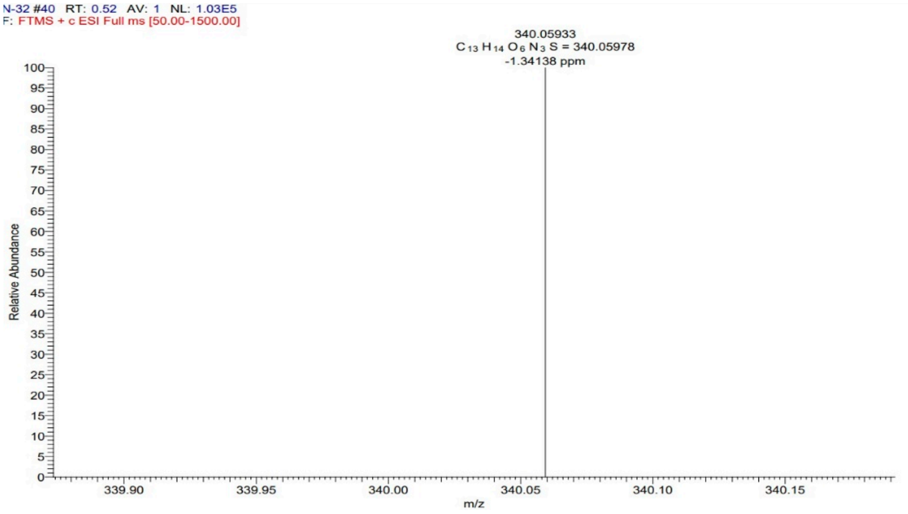

Compound 37: ^{1}H and ^{13}C NMR and HRMS spectra.

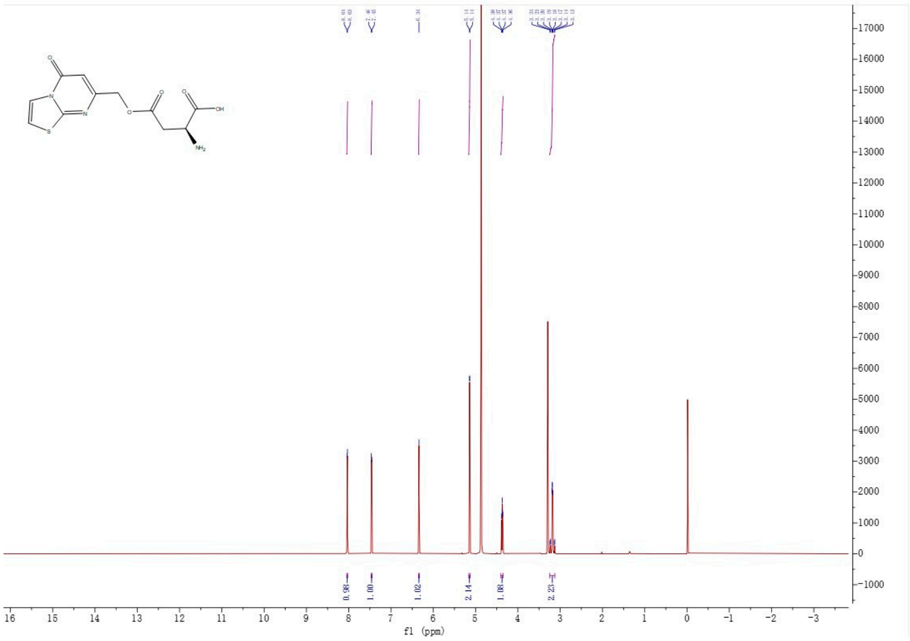

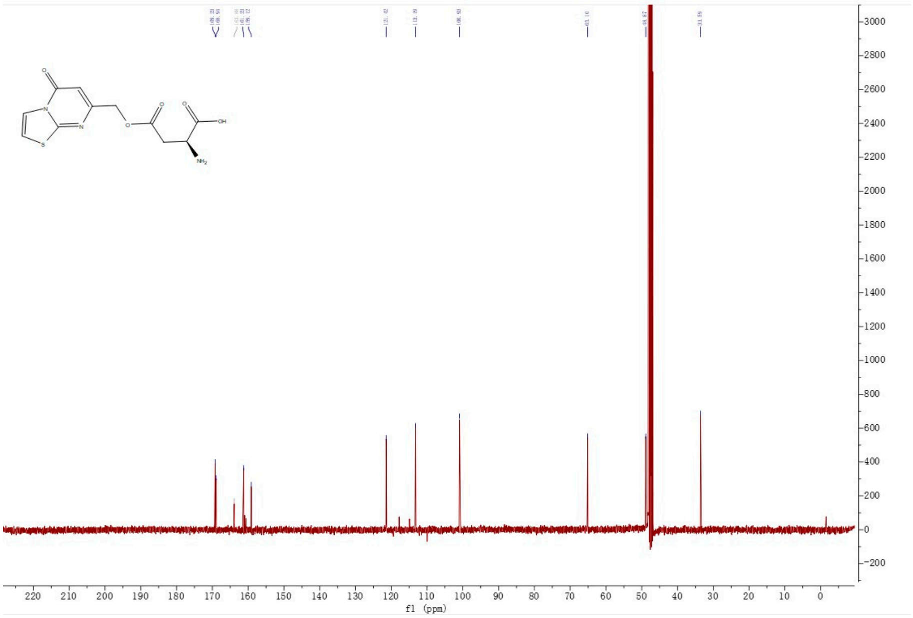

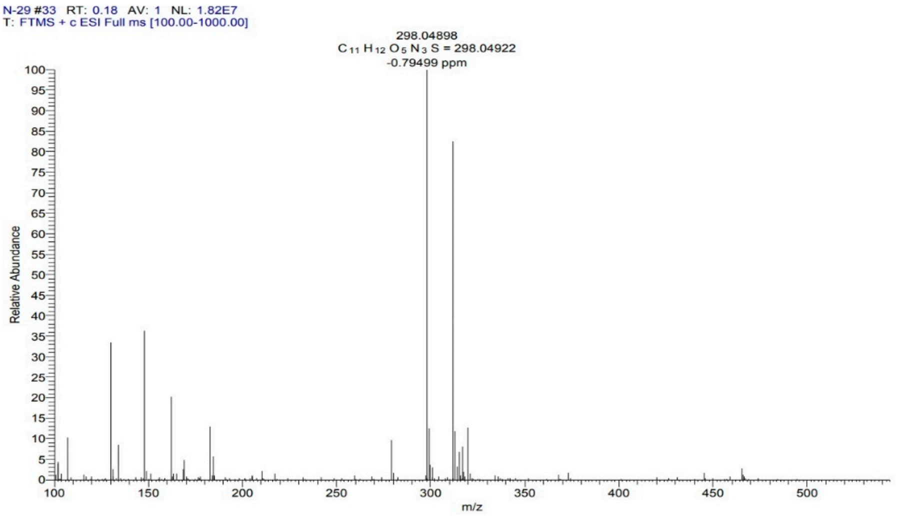

Compound 41: ^{1}H and ^{13}C NMR and HRMS spectra.

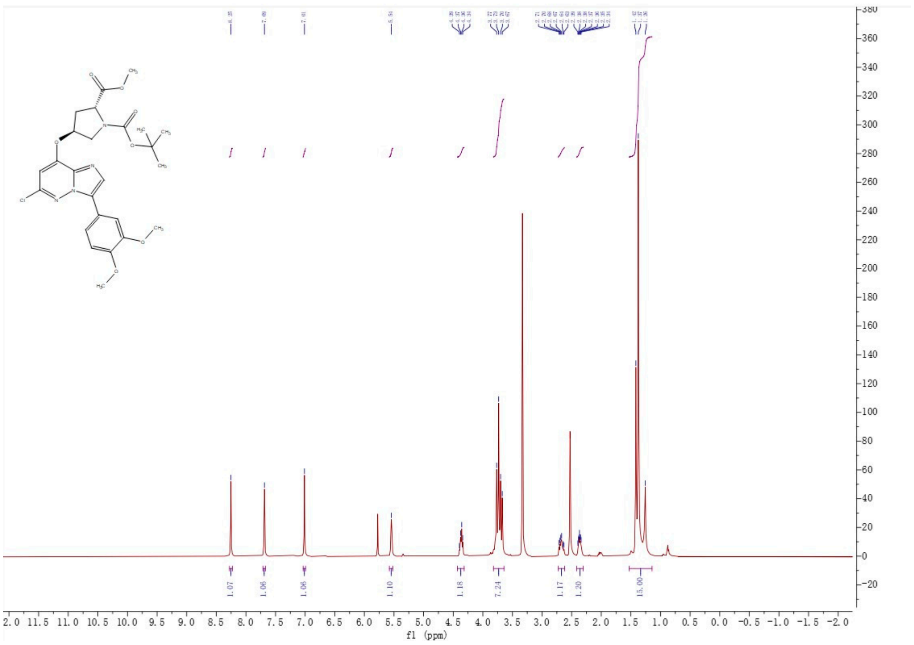
f1 (ppm)

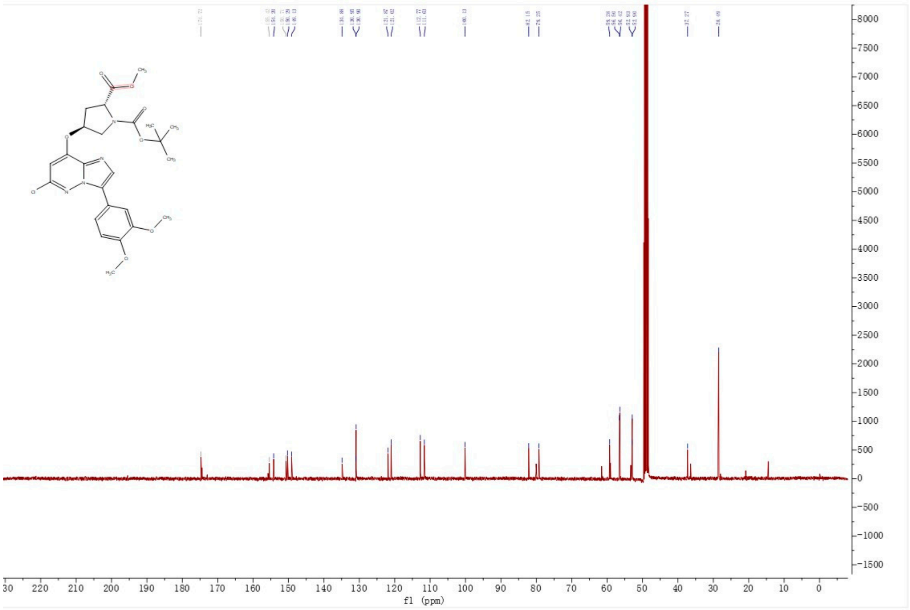
f1 (ppm)

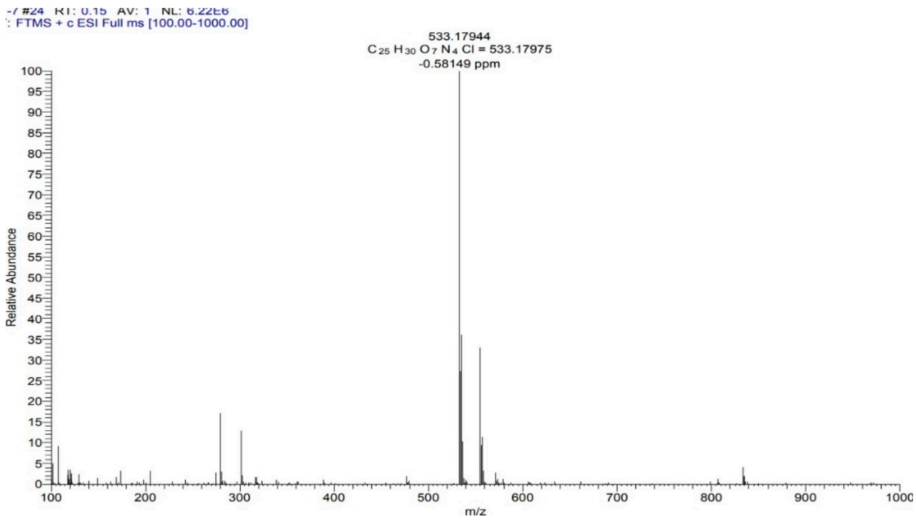

Compound 43: ^{1}H and ^{13}C NMR and HRMS spectra.

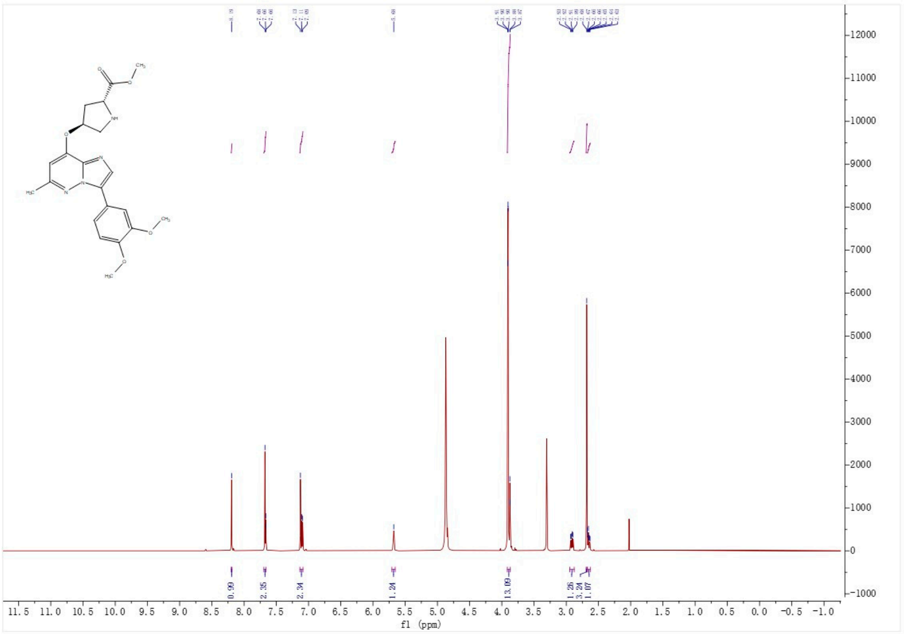

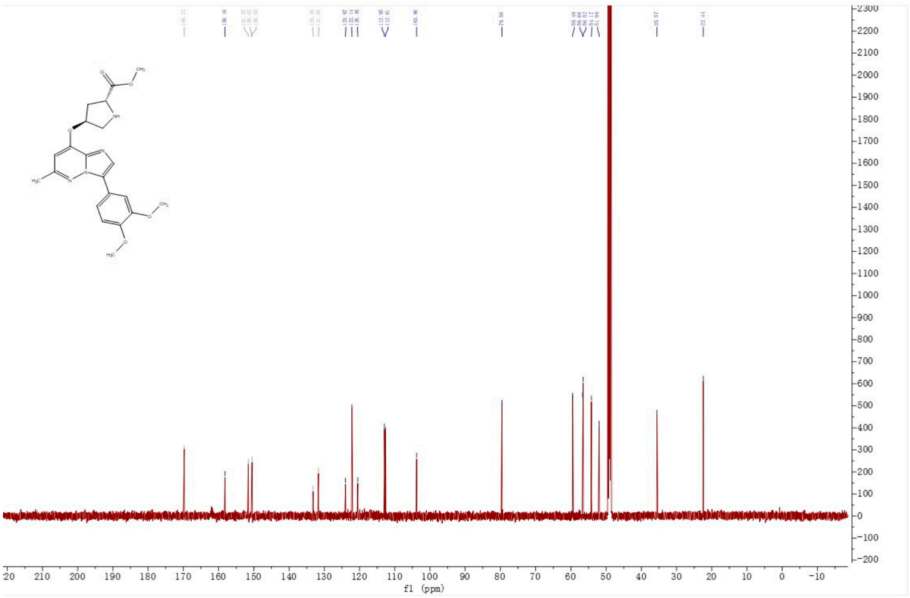

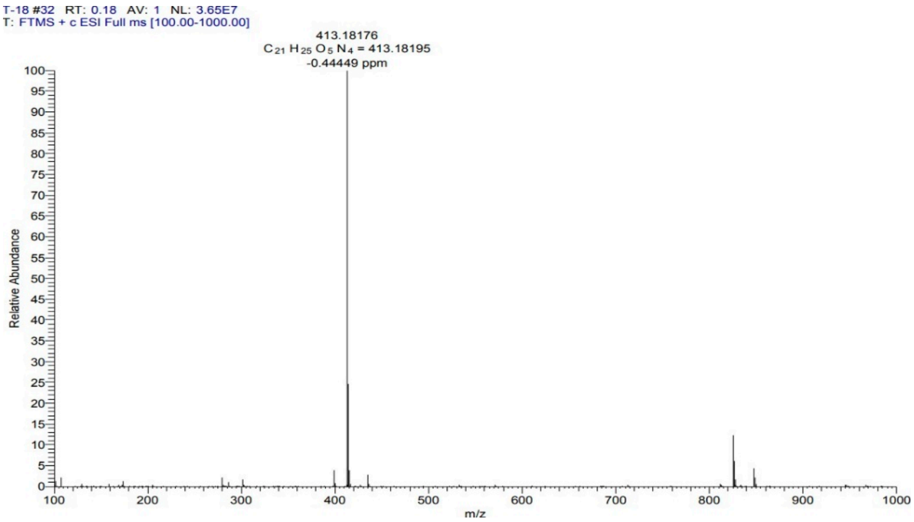

Compound 45: ^{1}H and ^{13}C NMR and HRMS spectra.

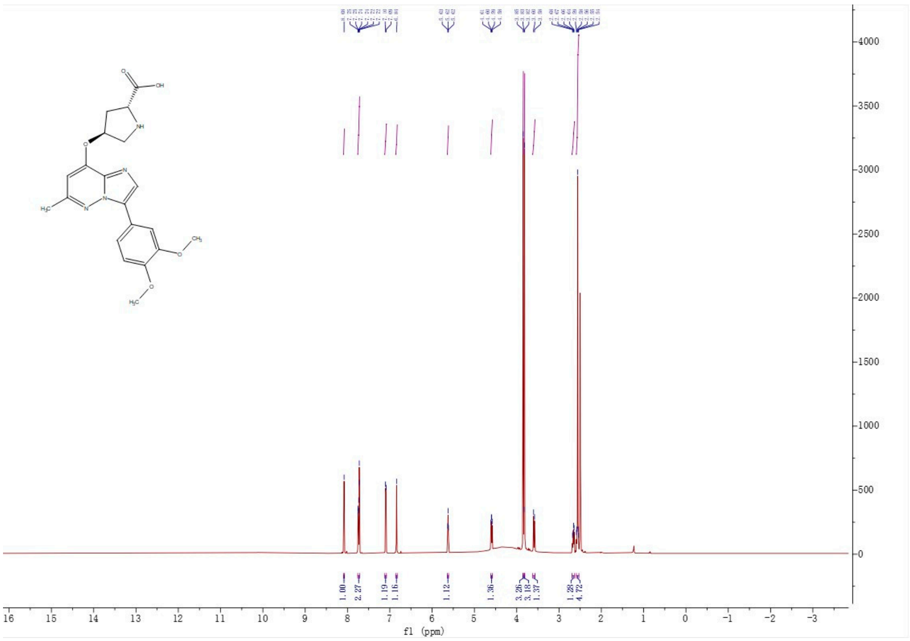

f1 (ppm)

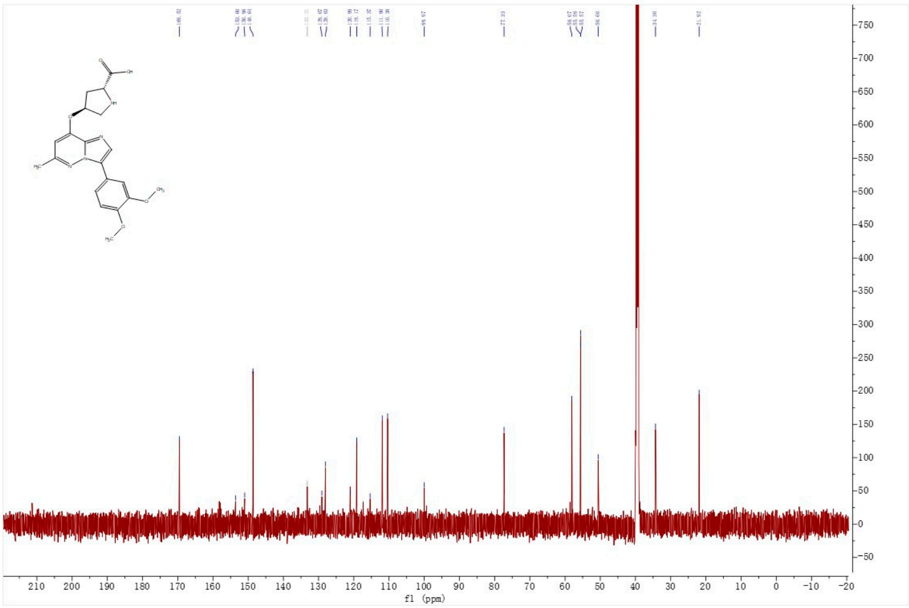

f1 (ppm)

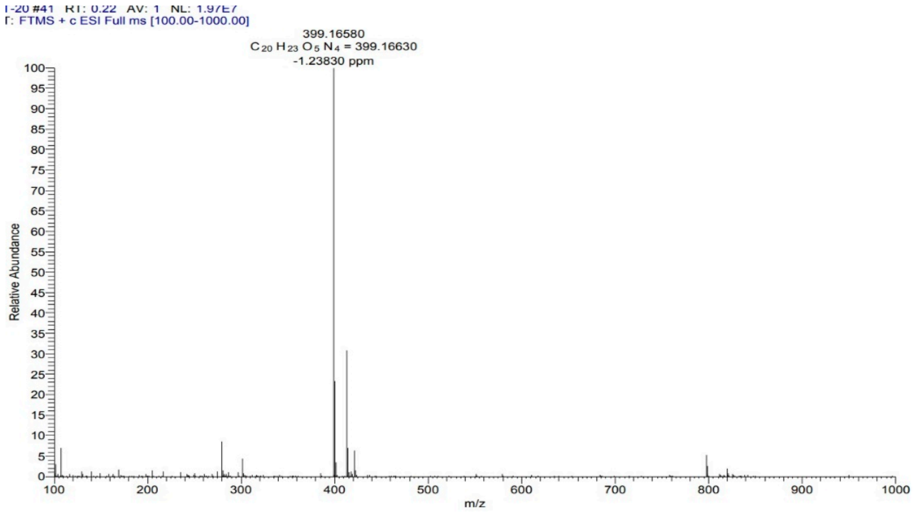

I-20 #41 RT: 0.22 AV: 1 NL: 1.97E7
T: FTMS + c ESI Full ms [100.00-1000.00]
399.16580
C 20 H 23 O 5 N 4 = 399.16630
-1.23830 ppm
Relative Abundance
m/z

HPLC traces for all target compounds

Compound 1

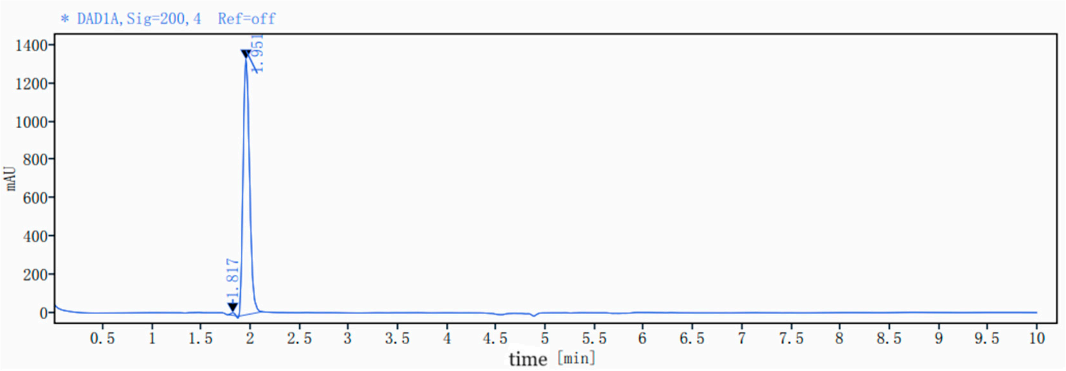

Signal: * DAD1A, Sig=200, 4 Ref=off

Retention time [min]	Type	Peak width [min]	Peak area	Height	Peak area%
1. 817	MM m	0. 04	24. 29	12. 10	0. 39
1. 951	MM m	0. 07	6133. 31	1336. 53	99. 61
		Total	6157. 61		

Compound 3

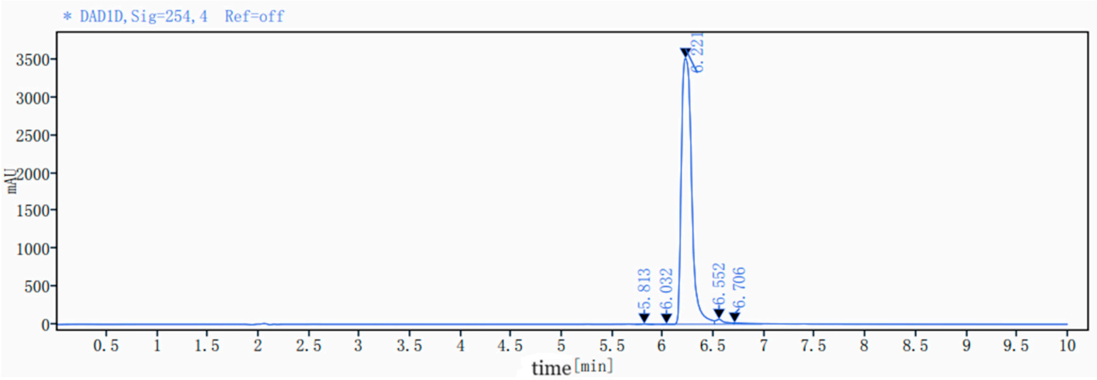

Signal: * DAD1D, Sig=254, 4 Ref=off

Retention time [min]	Type	Peak width [min]	Peak area	Height	Peak area%
5. 813	VB	0. 19	22. 83	5. 31	0. 09
6. 032	VB	0. 11	7. 80	2. 32	0. 03
6. 221	BV	0. 41	24866. 24	3516. 44	97. 62
6. 552	VV	0. 19	410. 13	63. 46	1. 61
6. 706	VV B	0. 28	165. 57	16. 04	0. 65
		Total	25472. 57		

Compound 4a

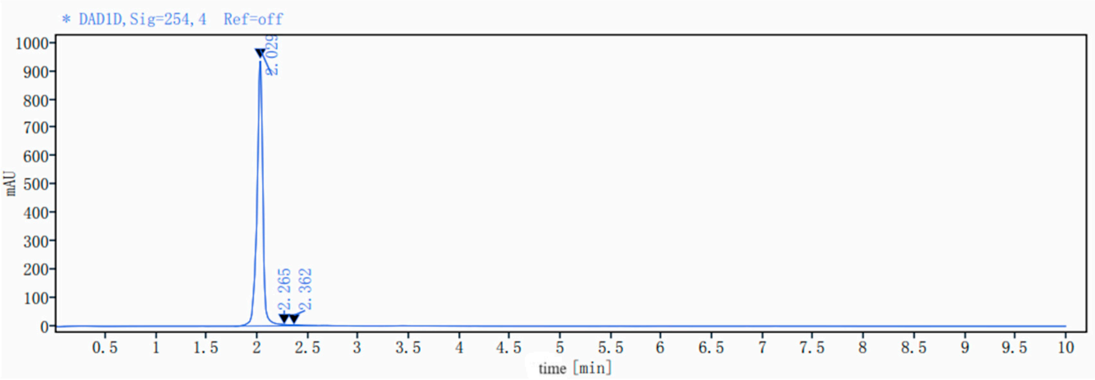

Signal: * DAD1D, Sig=254, 4 Ref=off

Retention time [min]	Type	Peak width [min]	Peak area	Height	Peak area%
2.029	BV	0.47	3768.18	942.96	98.81
2.265	VV B	0.07	18.53	4.76	0.49
2.362	VB	0.27	26.76	3.73	0.70
		Total	3813.47		

Compound 4b

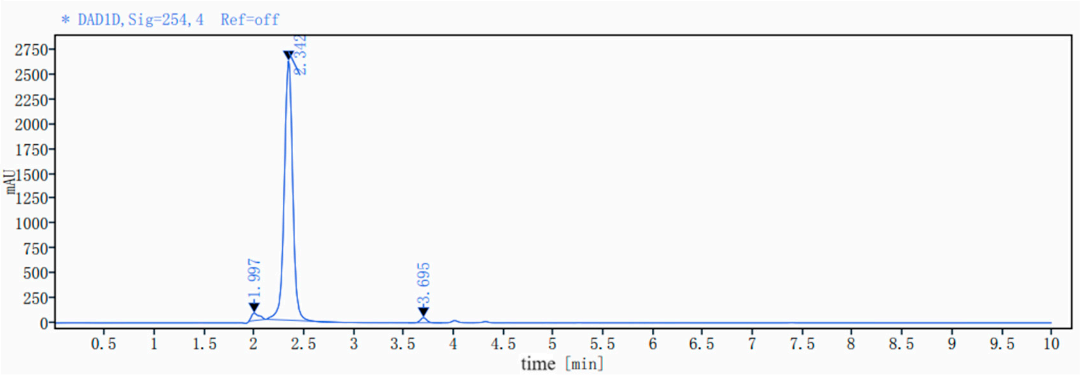

Signal: * DAD1D, Sig=254, 4 Ref=off

Retention time [min]	Type	Peak width [min]	Peak area	Height	Peak area%
1.997	MM m	0.08	402.19	75.44	2.51
2.342	MB m	0.09	15384.54	2616.05	96.08
3.695	BB	0.34	225.43	51.03	1.41
		Total	16012.16		

Compound 4c

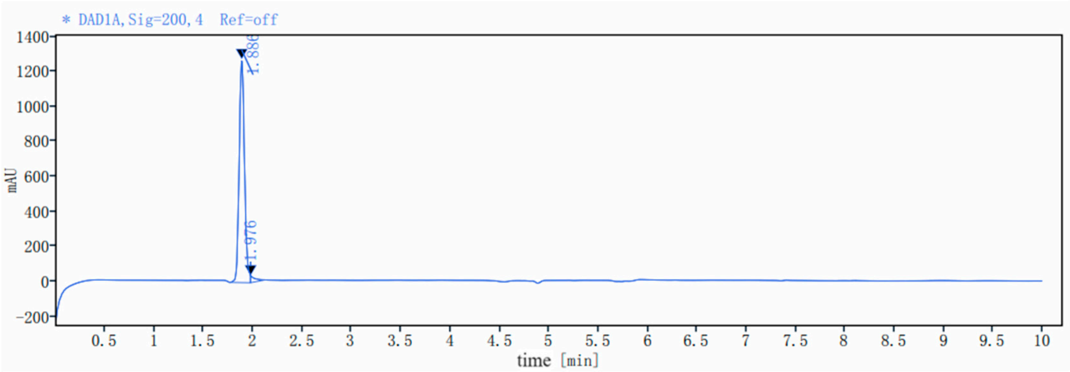

Signal: * DAD1A, Sig=200, 4 Ref=off

Retention time [min]	Type	Peak width [min]	Peak area	Height	Peak area%
1.886	MM m	0.06	4829.44	1277.15	97.43
1.976	MM m	0.05	127.24	41.09	2.57
		Total	4956.68		

Compound 4d

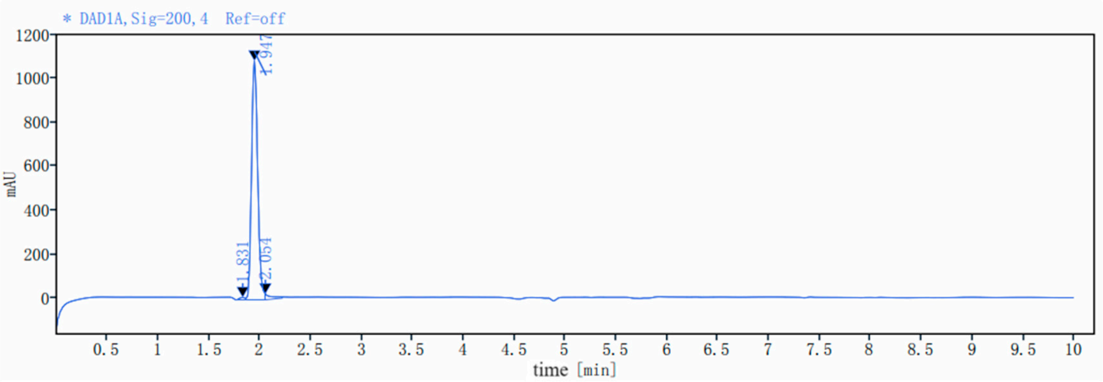

Signal: * DAD1A, Sig=200, 4 Ref=off

Retention time [min]	Type	Peak width [min]	Peak area	Height	Peak area%
1.831	BM m	0.06	37.38	10.30	0.80
1.947	MM m	0.06	4533.13	1085.73	96.71
2.054	MV m	0.07	116.71	26.15	2.49
		Total	4687.21		

Compound 5

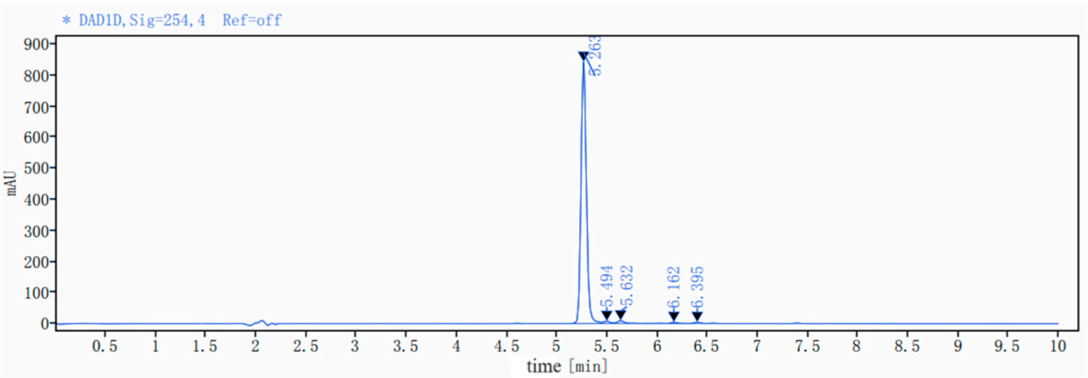

Signal: * DAD1D, Sig=254, 4 Ref=off

Retention time [min]	Type	Peak width [min]	Peak area	Height	Peak area%
5. 263	BV	0. 32	3170. 36	843. 28	97. 05
5. 494	VV	0. 12	31. 71	7. 12	0. 97
5. 632	VB	0. 16	36. 68	8. 69	1. 12
6. 162	BB	0. 23	17. 84	4. 29	0. 55
6. 395	MM m	0. 05	10. 28	3. 27	0. 31
		Total	3266. 87		

Compound 6

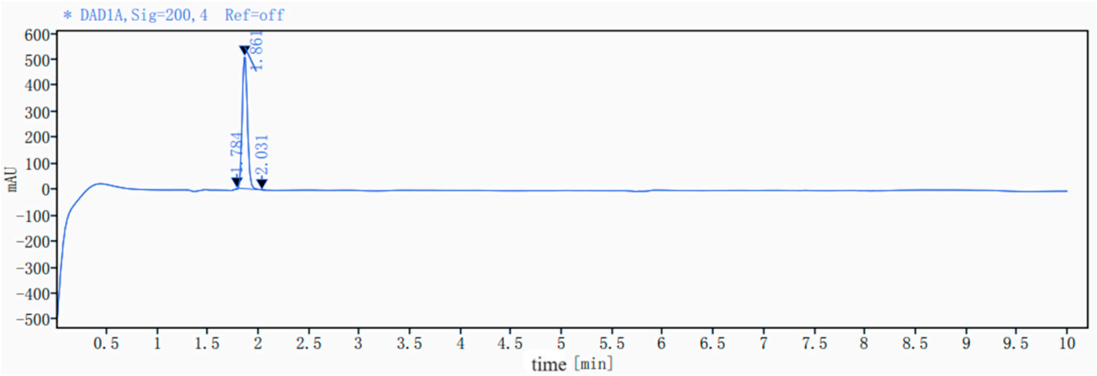

Signal: * DAD1A, Sig=200, 4 Ref=off

Retention time [min]	Type	Peak width [min]	Peak area	Height	Peak area%
1. 784	MM n	0. 02	2. 11	2. 36	0. 12
1. 861	MM m	0. 06	1817. 87	509. 78	99. 87
2. 031	MM m	0. 02	0. 24	0. 18	0. 01
		Total	1820. 23		

Compound 8a

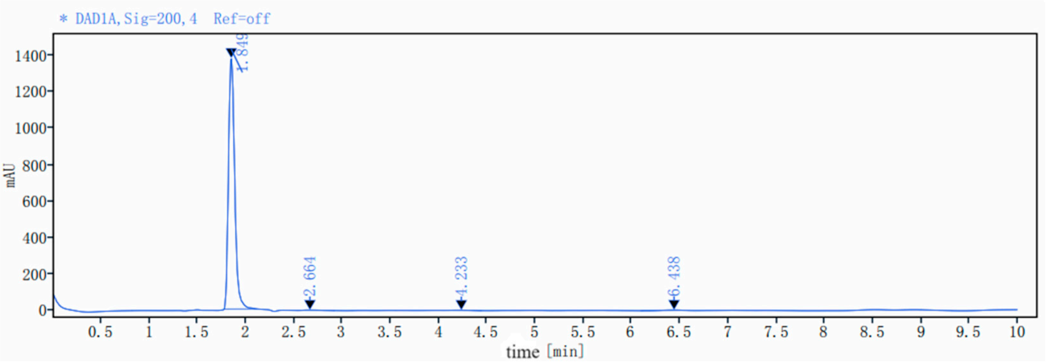

Signal: * DAD1A, Sig=200, 4 Ref=off

Retention time [min]	Type	Peak width [min]	Peak area	Height	Peak area%
1.849	MM m	0.07	6135.77	1378.19	98.82
2.664	MM m	0.19	22.57	1.81	0.36
4.233	MM m	0.20	25.12	1.74	0.40
6.438	MM m	0.19	25.77	2.17	0.42
		Total	6209.24		

Compound 8b

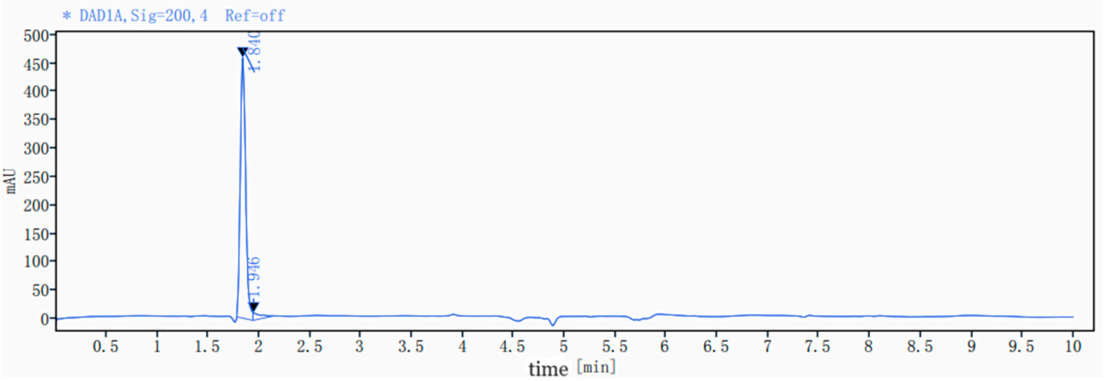

Signal: * DAD1A, Sig=200, 4 Ref=off

Retention time [min]	Type	Peak width [min]	Peak area	Height	Peak area%
1.840	MM m	0.06	1673.98	458.37	96.09
1.946	MM m	0.09	68.05	12.98	3.91
		Total	1742.04		

Compound 9

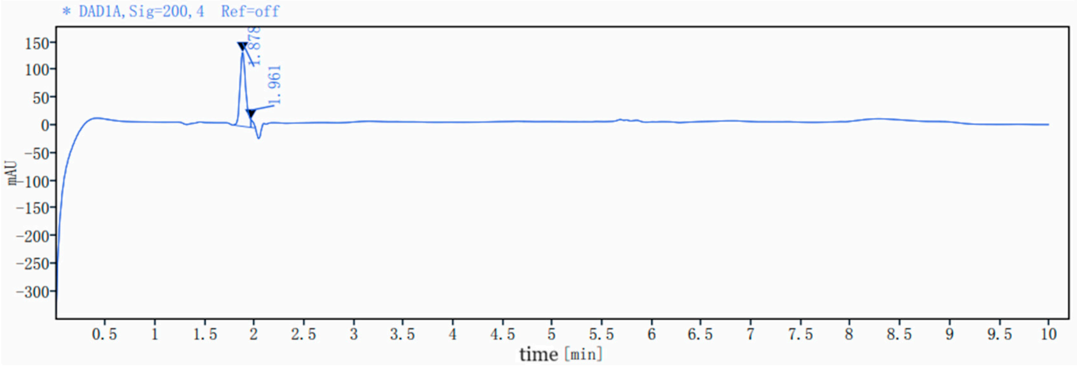

Signal: * DAD1A, Sig=200, 4 Ref=off

Retention time [min]	Type	Peak width [min]	Peak area	Height	Peak area%
1.878	MM m	0.06	540.99	133.74	95.64
1.961	MM m	0.03	24.68	14.25	4.36
		Total	565.67		

Compound 11a

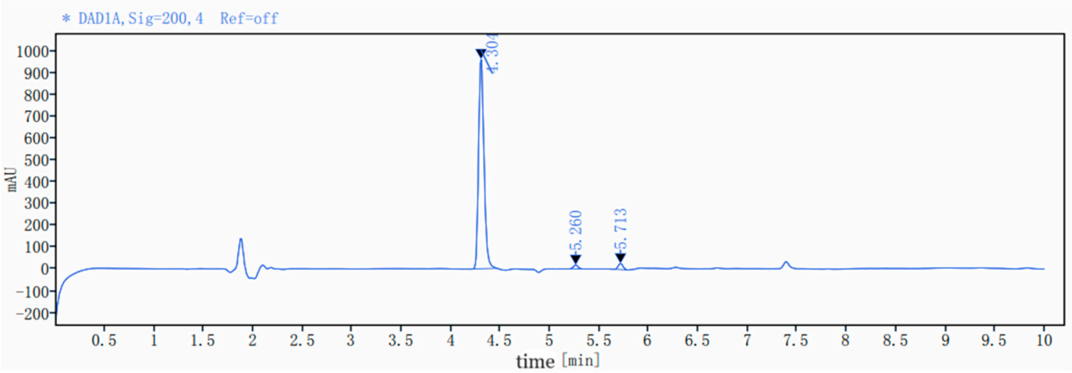

Signal: * DAD1A, Sig=200, 4 Ref=off

Retention time [min]	Type	Peak width [min]	Peak area	Height	Peak area%
4.304	MM m	0.06	3694.68	959.93	96.17
5.260	MM m	0.05	54.66	16.94	1.42
5.713	MB m	0.05	92.68	27.14	2.41
		Total	3842.02		

Compound 11b

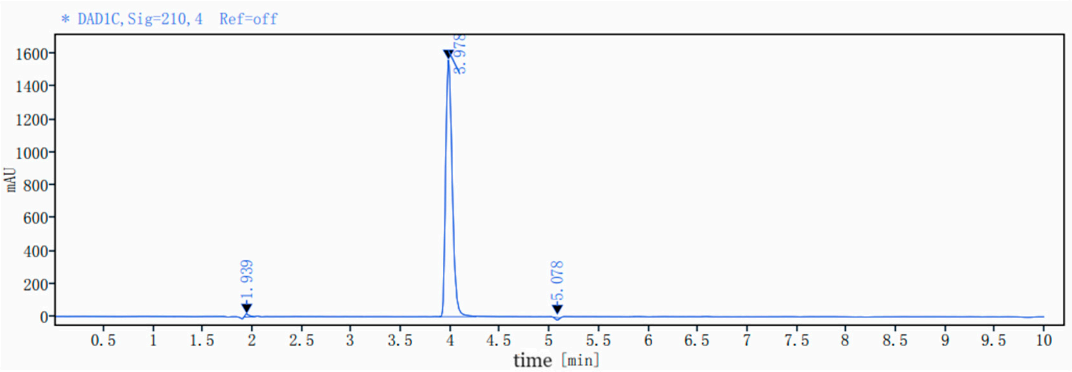

Signal: * DAD1C, Sig=210, 4 Ref=off

Retention time [min]	Type	Peak width [min]	Peak area	Peak height	Peak area%
1.939	MM m	0.05	51.29	18.48	0.70
3.978	BV	0.37	7219.24	1562.53	98.76
5.078	MM n	0.04	39.14	14.84	0.54
		Total	7309.67		

Compound 11c

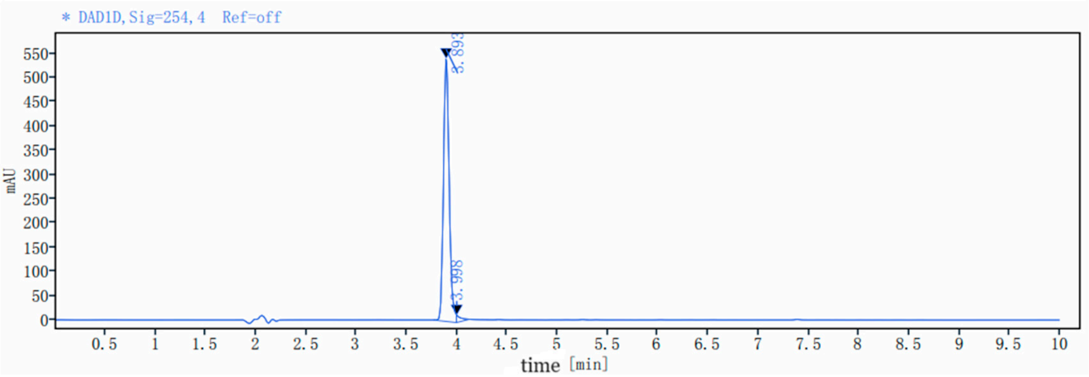

Signal: * DAD1D, Sig=254, 4 Ref=off

Retention time [min]	Type	Peak width [min]	Peak area	Height	Peak area%
3.893	BM m	0.06	2171.47	542.21	97.97
3.998	MM m	0.05	45.02	15.00	2.03
		Total	2216.50		

Compound 11d

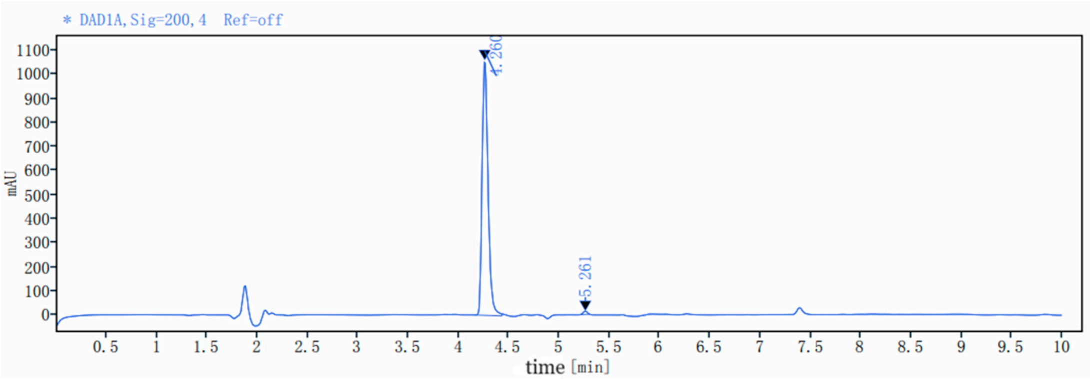

Signal: * DAD1A, Sig=200, 4 Ref=off

Retention time [min]	Type	Peak width [min]	Peak area	Height	Peak area%
4. 260	MM m	0. 06	4339. 40	1059. 55	99. 12
5. 261	MM m	0. 05	38. 52	13. 22	0. 88
		Total	4377. 92		

Compound 11e

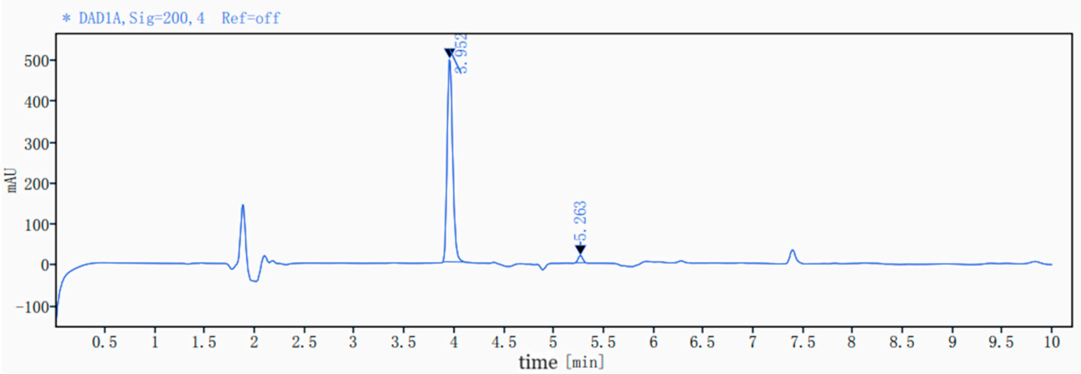

Signal: * DAD1A, Sig=200, 4 Ref=off

Retention time [min]	Type	Peak width [min]	Peak area	Height	Peak area%
3. 952	MM m	0. 06	1904. 79	496. 07	97. 24
5. 263	MM m	0. 05	53. 98	17. 55	2. 76
		Total	1958. 77		

Compound 11f

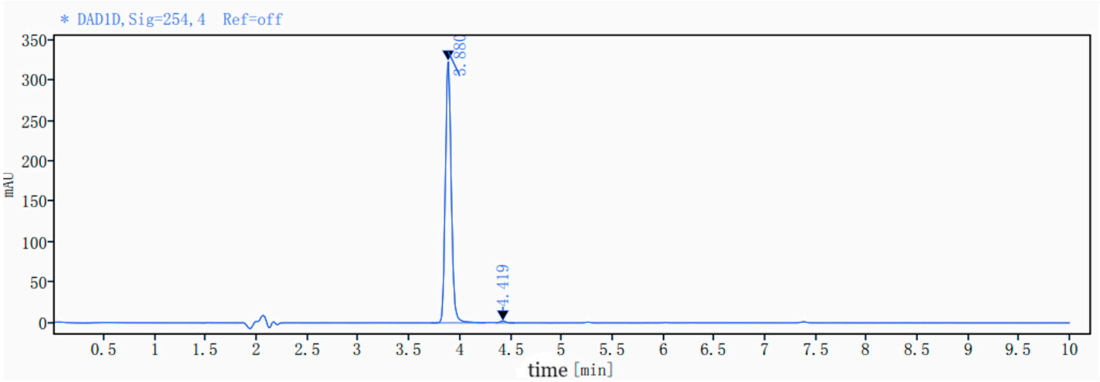

Signal: * DAD1D, Sig=254, 4 Ref=off

Retention time [min]	Type	Peak width [min]	Peak area	Height	Peak area%
3. 880	BV	0. 53	1313. 94	324. 20	99. 31
4. 419	VB	0. 19	9. 08	2. 32	0. 69
		Total	1323. 01		

Compound 20a

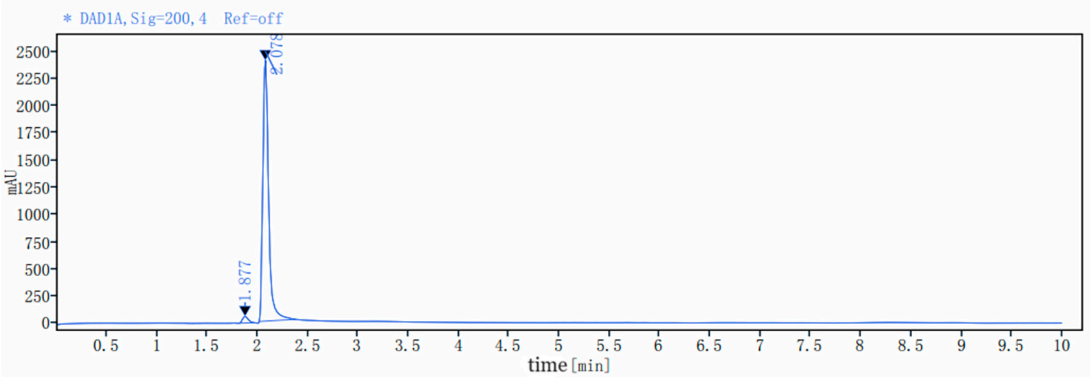

Signal: * DAD1A, Sig=200, 4 Ref=off

Retention time [min]	Type	Peak width [min]	Peak area	Height	Peak area%
1. 877	BB	0. 15	236. 88	60. 30	2. 40
2. 078	BM m	0. 06	9623. 67	2396. 03	97. 60
		Total	9860. 54		

Compound 20b

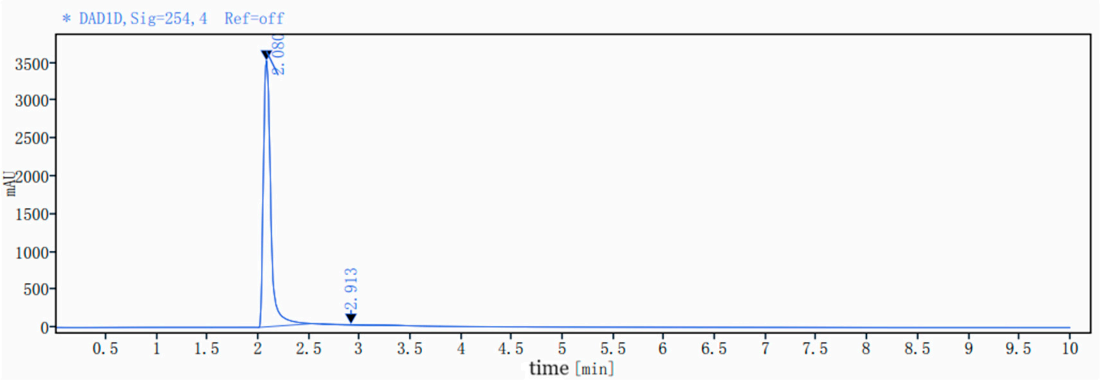

Signal: * DAD1D, Sig=254, 4 Ref=off

Retention time [min]	Type	Peak width [min]	Peak area	Height	Peak area%
2.080	BM m	0.08	17911.79	3519.99	99.42
2.913	MM n	0.34	103.77	5.14	0.58
		Total	18015.55		

Compound 23a

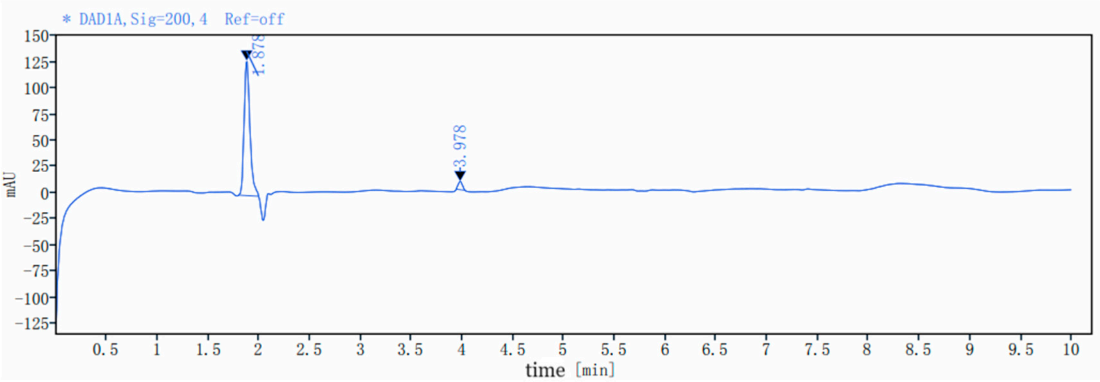

Signal: * DAD1A, Sig=200, 4 Ref=off

Retention time [min]	Type	Peak width [min]	Peak area	Height	Peak area%
1.878	MM m	0.06	513.75	128.95	95.82
3.978	MM m	0.05	22.44	7.81	4.18
		Total	536.18		

Compound 23b

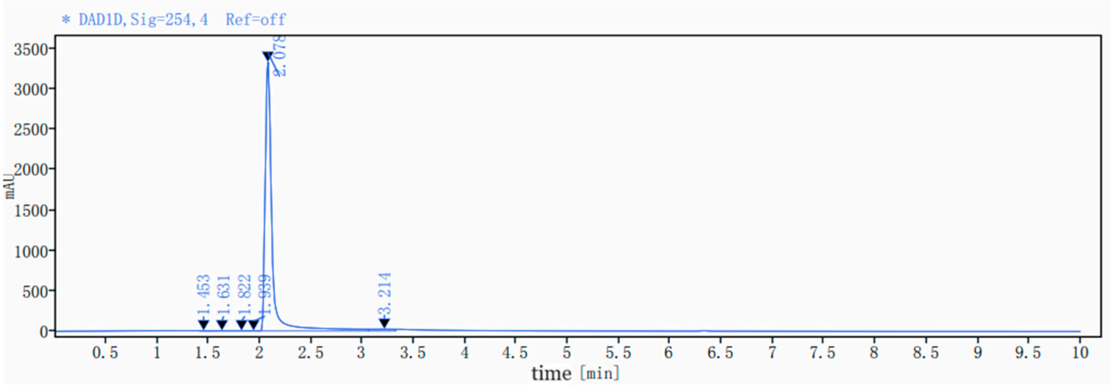

Signal: * DAD1D, Sig=254, 4 Ref=off

Retention time [min]	Type	Peak width [min]	Peak area	Height	Peak area%
1.453	VV B	0.10	19.31	3.46	0.12
1.631	VV	0.27	47.22	3.12	0.30
1.822	VV	0.11	15.43	2.46	0.10
1.939	VB	0.11	11.47	2.02	0.07
2.078	BV	1.05	15465.18	3324.34	97.25
3.214	VV	0.27	344.21	21.81	2.16
		Total	15902.82		

Compound 28a

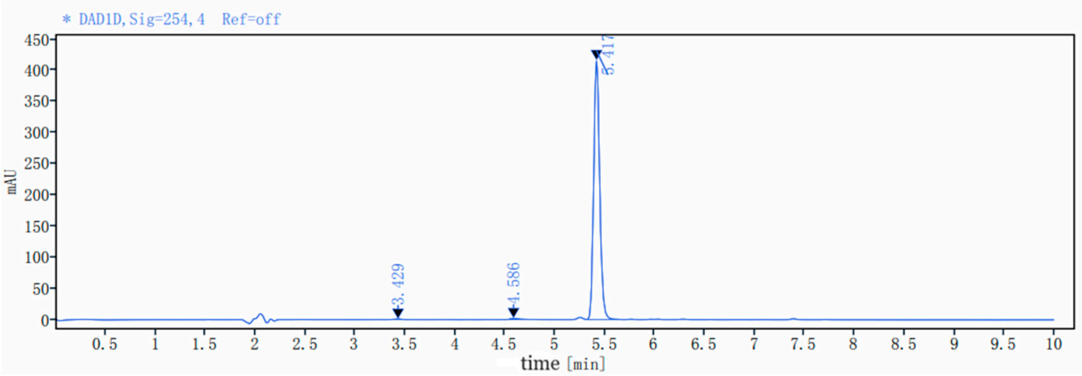

Signal: * DAD1D, Sig=254, 4 Ref=off

Retention time [min]	Type	Peak width [min]	Peak area	Height	Peak area%
3.429	MM m	0.07	3.40	0.78	0.19
4.586	MM m	0.08	9.99	1.86	0.57
5.417	VB	0.38	1749.02	416.19	99.24
		Total	1762.40		

Compound 29

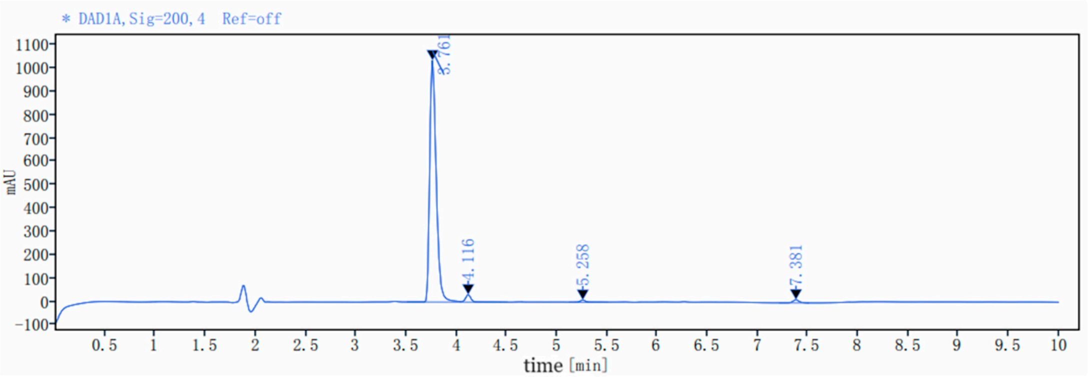

Signal: * DAD1A, Sig=200, 4 Ref=off

Retention time [min]	Type	Peak width [min]	Peak area	Height	Peak area%
3. 761	BV	0. 53	4667. 07	1034. 05	95. 32
4. 116	VB	0. 47	125. 95	31. 50	2. 57
5. 258	VV	0. 39	46. 27	9. 26	0. 94
7. 381	VB	0. 30	56. 83	13. 23	1. 16
		Total	4896. 12		

Compound 31

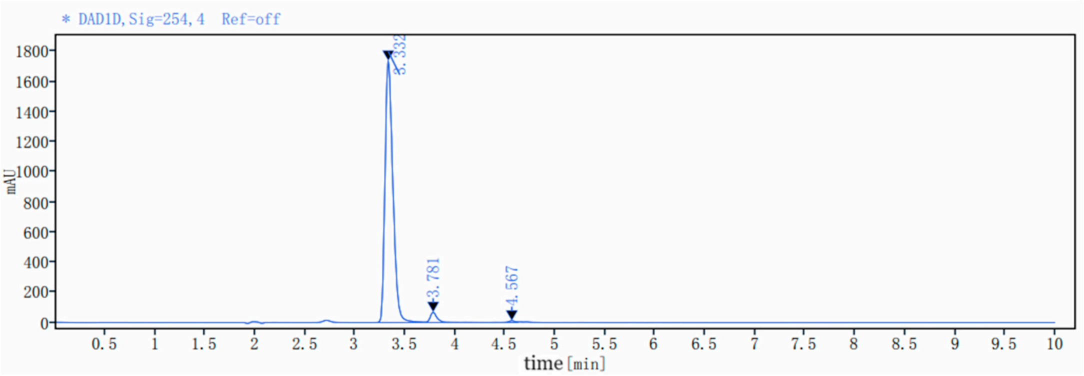

Signal: * DAD1D, Sig=254, 4 Ref=off

Retention time [min]	Type	Peak width [min]	Peak area	Height	Peak area%
3. 332	BV	0. 54	9530. 45	1739. 16	96. 32
3. 781	VB	0. 35	314. 98	68. 06	3. 18
4. 567	BV	0. 17	48. 92	11. 80	0. 49
		Total	9894. 35		

Compound 32a

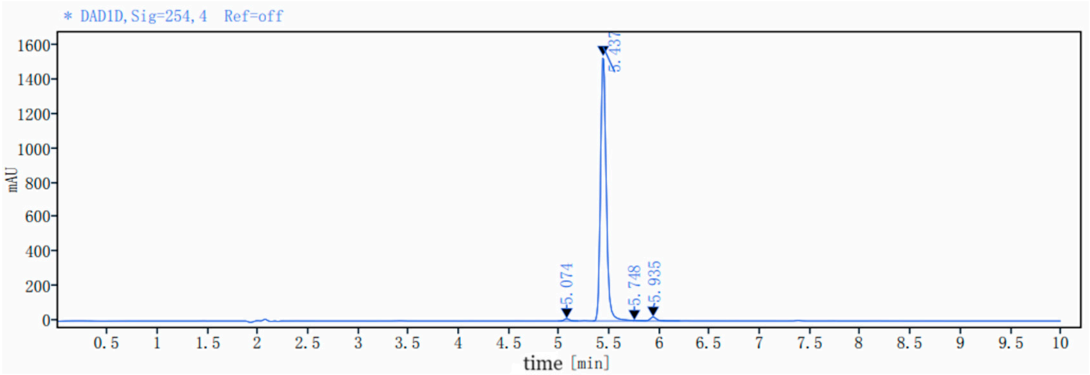

Signal: * DAD1D, Sig=254, 4 Ref=off

Retention time [min]	Type	Peak width [min]	Peak area	Height	Peak area%
5.074	BV	0.20	49.44	13.49	0.79
5.437	BV	0.41	6073.24	1532.21	97.34
5.748	VV B	0.11	15.00	3.18	0.24
5.935	VB	0.35	101.61	22.34	1.63
		Total	6239.28		

Compound 33

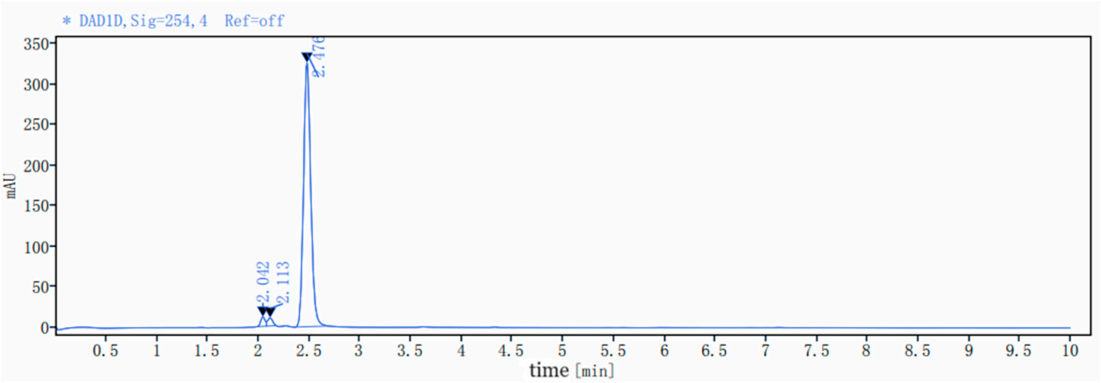

Signal: * DAD1D, Sig=254, 4 Ref=off

Retention time [min]	Type	Peak width [min]	Peak area	Height	Peak area%
2.042	MM m	0.05	33.92	11.38	2.02
2.113	MM m	0.06	34.66	9.83	2.07
2.476	BM m	0.08	1609.89	325.34	95.91
		Total	1678.48		

Compound 34a

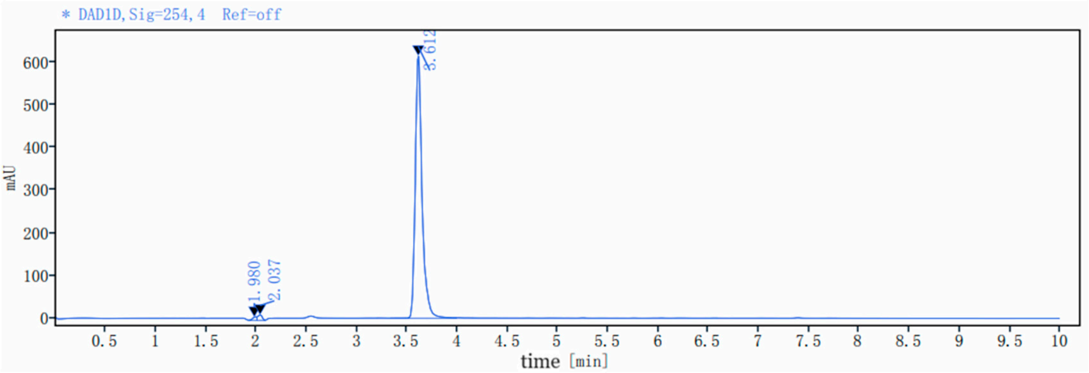

Signal: * DAD1D, Sig=254, 4 Ref=off

Retention time [min]	Type	Peak width [min]	Peak area	Height	Peak area%
1.980	BV F	0.08	21.26	7.39	0.72
2.037	VB	0.08	36.75	12.53	1.24
3.612	BV	0.66	2911.30	613.26	98.05
		Total	2969.31		

Compound 35

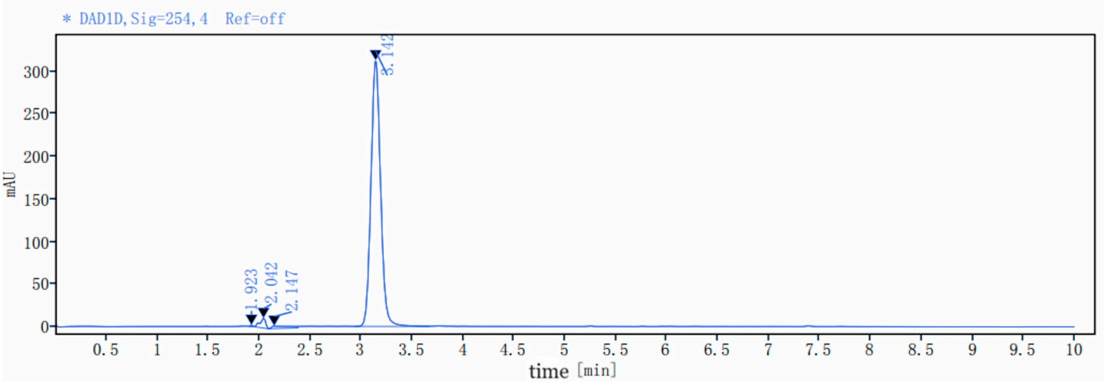

Signal: * DAD1D, Sig=254, 4 Ref=off

Retention time [min]	Type	Peak width [min]	Peak area	Height	Peak area%
1.923	VB	0.05	2.94	1.74	0.14
2.042	BB	0.14	43.99	11.35	2.06
2.147	BV	0.29	36.82	2.90	1.72
3.142	BV	0.74	2051.62	312.39	96.08
		Total	2135.38		

Compound 37

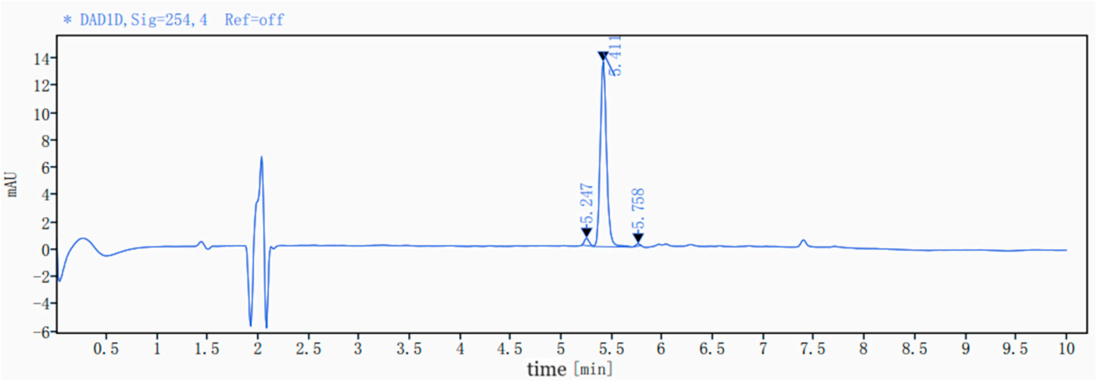

Signal: * DAD1D, Sig=254, 4 Ref=off

Retention time [min]	Type	Peak width [min]	Peak area	Height	Peak area%
5. 247	MM m	0. 05	1. 79	0. 53	2. 93
5. 411	VV	0. 38	58. 80	13. 54	96. 30
5. 758	MM m	0. 05	0. 47	0. 17	0. 77
		Total	61. 06		

Compound 41

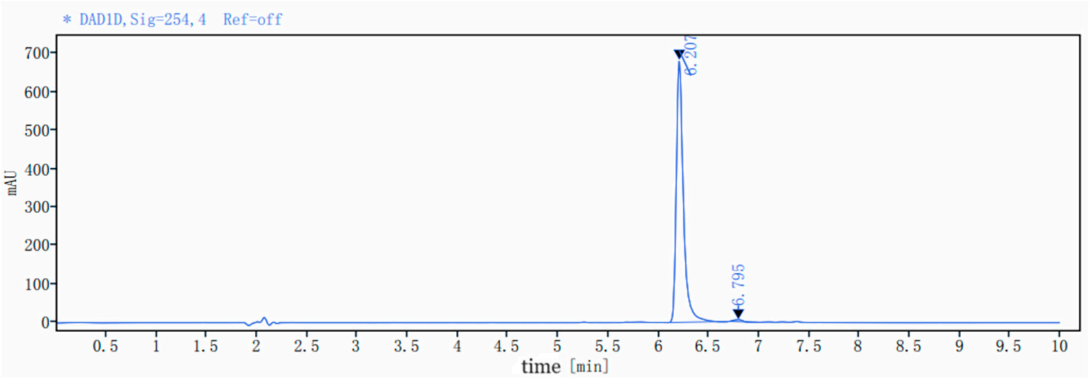

Signal: * DAD1D, Sig=254, 4 Ref=off

Retention time [min]	Type	Peak width [min]	Peak area	Height	Peak area%
6. 207	BM m	0. 07	3354. 33	681. 82	99. 11
6. 795	MB m	0. 10	30. 28	5. 31	0. 89
		Total	3384. 61		

Compound 43

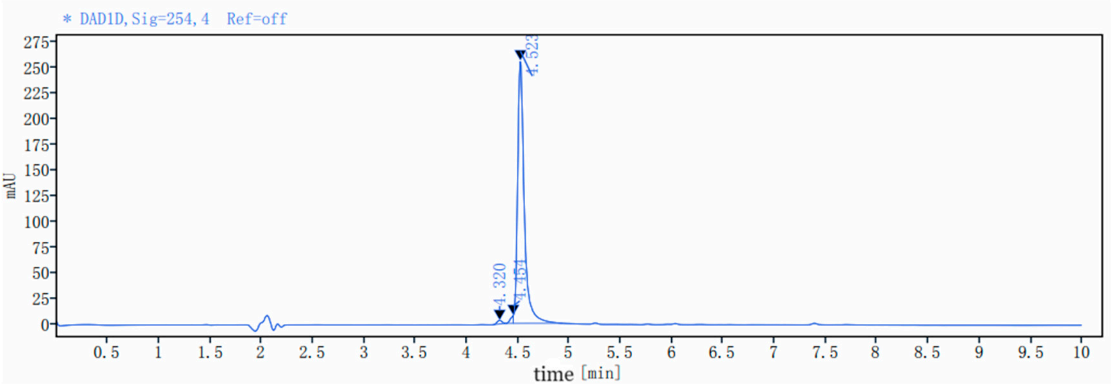

Signal:	* DAD1D, Sig=254, 4 Ref=off				
**Retention time [min]**	**Type**	**Peak width [min]**	**Peak area**	**Height**	**Peak area%**
4. 320	BB	0. 12	11. 42	3. 49	0. 98
4. 454	BM m	0. 03	13. 63	7. 65	1. 17
4. 523	MM m	0. 07	1143. 45	254. 68	97. 86
		Total	1168. 50		

Compound 45

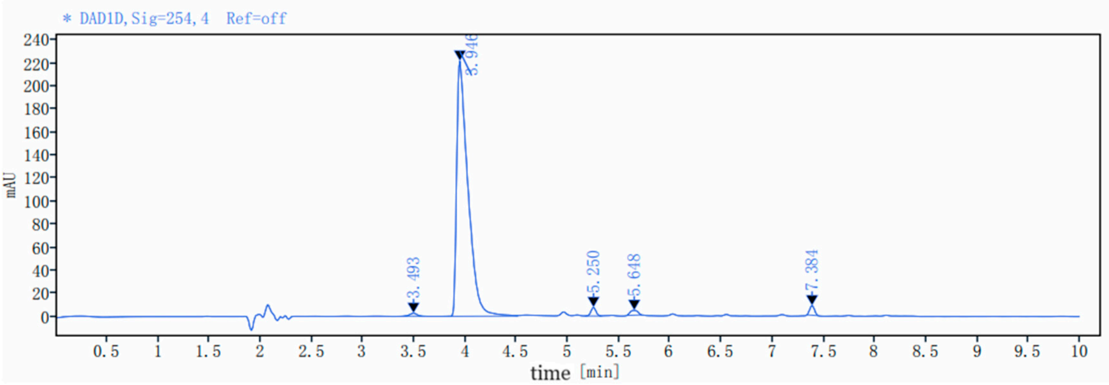

Signal:	* DAD1D, Sig=254, 4 Ref=off				
**Retention time [min]**	**Type**	**Peak width [min]**	**Peak area**	**Height**	**Peak area%**
3. 493	MM m	0. 08	12. 13	2. 50	0. 68
3. 946	BV	0. 73	1694. 36	222. 13	95. 13
5. 250	BV	0. 16	25. 44	7. 35	1. 43
5. 648	MM m	0. 09	22. 05	4. 16	1. 24
7. 384	MM m	0. 05	27. 08	8. 25	1. 52
		Total	1781. 06		
